# Isolation and analysis of the genetic diversity of repertoires of *VSG *expression site containing telomeres from *Trypanosoma brucei gambiense, T. b. brucei *and *T. equiperdum*

**DOI:** 10.1186/1471-2164-9-385

**Published:** 2008-08-12

**Authors:** Rosanna Young, Jesse E Taylor, Ayako Kurioka, Marion Becker, Edward J Louis, Gloria Rudenko

**Affiliations:** 1 Peter Medawar Building for Pathogen Research, University of Oxford, South Parks Road, Oxford, OX1 3SY, UK; 2 Department of Statistics, University of Oxford, 1 South Parks Road, Oxford, OX1 3TG, UK; 3 Institute of Genetics, Queens Medical Centre, University of Nottingham, Nottingham, NG7 2UH, UK

## Abstract

**Background:**

African trypanosomes (including *Trypanosoma brucei*) are unicellular parasites which multiply in the mammalian bloodstream. *T. brucei *has about twenty telomeric bloodstream form Variant Surface Glycoprotein (*VSG*) expression sites (BESs), of which one is expressed at a time in a mutually exclusive fashion. BESs are polycistronic transcription units, containing a variety of families of expression site associated genes (*ESAG*s) in addition to the telomeric *VSG*. These polymorphic *ESAG *families are thought to play a role in parasite-host adaptation, and it has been proposed that ESAG diversity might be related to host range. Analysis of the genetic diversity of these telomeric gene families has been confounded by the underrepresentation of telomeric sequences in standard libraries. We have previously developed a method to selectively isolate sets of trypanosome BES containing telomeres using Transformation associated recombination (TAR) cloning in yeast.

**Results:**

Here we describe the isolation of repertoires of BES containing telomeres from three trypanosome subspecies: *Trypanosoma brucei gambiense *DAL 972 (causative agent of West-African trypanosomiasis), *T. b. brucei *EATRO 2340 (a nonhuman infective strain) and *T. equiperdum *STIB 818 (which causes a sexually transmitted disease in equines). We have sequenced and analysed the genetic diversity at four BES loci (BES promoter region, *ESAG6*, *ESAG5 *and *ESAG2*) from these three trypanosome BES repertoires.

**Conclusion:**

With the exception of ESAG2, the BES sequence repertoires derived from *T. b. gambiense *are both less diverse than and nearly reciprocally monophyletic relative to those from *T. b. brucei *and *T. equiperdum*. Furthermore, although we find evidence for adaptive evolution in all three ESAG repertoires in *T. b. brucei *and *T. equiperdum*, only ESAG2 appears to be under diversifying selection in *T. b. gambiense*. This low level of variation in the *T. b. gambiense *BES sequence repertoires is consistent both with the relatively narrow host range of this subspecies and its apparent long-term clonality. However, our data does not show a clear correlation between size of trypanosome host range and either number of BESs or extent of *ESAG *genetic diversity.

## Background

Telomeres are preferred genomic locations for gene families involved in virulence and pathogenicity in a wide range of microbial pathogens including many unicellular eukaryotic parasites [1,2]. High rates of recombination at telomeres presumably facilitate the generation of genetic diversity [3], as has been postulated for the malaria parasite *Plasmodium*, where antigenically variable *VAR *genes are located predominantly at chromosome ends [4,5]. The prokaryote *Borrelia hermsii*, causative agent of relapsing fever, also has large numbers of variable antigen genes located on linear plasmids which can undergo gene conversion events allowing antigenic variation [6]. Lastly, African trypanosomes have segregated large gene families involved in antigenic variation or host adaptation to the telomeres of a broad range of chromosomes [1,7].

The African trypanosome *Trypanosoma brucei *causes African Sleeping Sickness in humans, which is transmitted by tsetse flies and is endemic to sub-Saharan Africa [8]. West African trypanosomiasis, comprising more than 90% of human cases, is the chronic form of the disease and is caused by *T. b. gambiense *[9]. In contrast, East African trypanosomiasis is an acute infection in humans caused by *T. b. rhodesiense*, with death occurring typically within 6–8 months in the absence of treatment [8]. *T. b. rhodesiense *is considered to be a zoonotic pathogen with a very extensive animal reservoir including a wide range of large game animals [10], while *T. b. gambiense *infects a more restricted range of reservoir hosts [11]. African trypanosomes also cause debilitating disease in livestock. *T. b. brucei *infects a variety of livestock and wildlife species, but is not human infective. Genetically, *T. b. brucei *is extremely similar to *T. b. rhodesiense*, but lacks the serum resistance associated gene *SRA *conferring human serum resistance [12,13]. Another non-human trypanosomiasis is dourine in equines, which is caused by *T. equiperdum*. This trypanosome, is also closely related to *T. brucei *[14], but has lost the ability to cycle through tsetse flies and instead is sexually transmitted [15]. *T. equiperdum *therefore has a much more restricted host range than other vector born *Trypanosoma *species.

Many subspecies of trypanosomes can multiply extracellularly in the mammalian bloodstream, where they are exposed to continuous attack both by antibodies and complement. Key to survival is a dense protective coat of Variant Surface Glycoprotein (VSG) [16], which is essential for the bloodstream form trypanosome even *in vitro *[17]. As trypanosomes can switch between expression of different VSGs, new trypanosome antigenic types can escape host antibodies raised against the old VSG, and can avoid eradication by antibody-mediated lysis allowing a chronic infection to be maintained [18-20]. Although a single trypanosome has up to 1500 *VSG *genes and pseudogenes [21,22], only one *VSG *is expressed at a time from one of about twenty bloodstream form *VSG *expression sites (BESs) [23,24].

BESs are polycistronic transcription units located at telomeres, which contain a variety of Expression Site Associated Gene (*ESAG*) families in addition to the telomeric *VSG *[25,26]. It has been hypothesised that some *ESAG*s encode surface proteins or receptors which play a role in adaptation of the trypanosome to life in different species of mammalian host [27,28]. For example, *ESAG6 *and *ESAG7 *encode polymorphic subunits of a trypanosome encoded receptor for host transferrin, the variable nature of which could affect the ability of the trypanosome to take up transferrin molecules from different species of mammalian host [27,29]. In addition, it has been shown that the *SRA *gene located within some BESs confers resistance to human serum [30]. As the trypanosome switches between different BESs, it expresses different permutations and combinations of polymorphic *ESAG*s [27,28,31]. The telomeric location of *VSG *expression sites could play a role in their control [1,7]. Additionally, high rates of recombination at telomere ends could play a role in generating genetic diversity, as has been proposed for variant antigen genes located at the telomeres of the malaria parasite *Plasmodium falciparum *[2,4].

Although a number of BESs from *T. b. brucei *and *T. b. rhodesiense *have been sequenced [25,26,30,32], relatively little is known about BESs in *T. brucei gambiense*, although they are clearly very similar to those from *T. b. brucei *[33]. We are interested in the genetic diversity of telomeric BESs, particularly with regards to whether BES repertoires derived from trypanosomes with a large host range show evidence for greater genetic diversity compared with BES repertoires isolated from trypanosomes which infect a more restricted number of mammalian species. As telomeric regions are notoriously difficult to isolate and characterise, and are typically very underrepresented in standard libraries, we previously developed a method to isolate entire repertoires of BES containing telomeres in yeast [34]. Here, we present the isolation and analysis of the genetic diversity found within repertoires of BES containing telomeres from three different species or subspecies of trypanosomes.

## Results and Discussion

### Isolation of BES containing telomere libraries from three trypanosomatids

**Figure 1:**
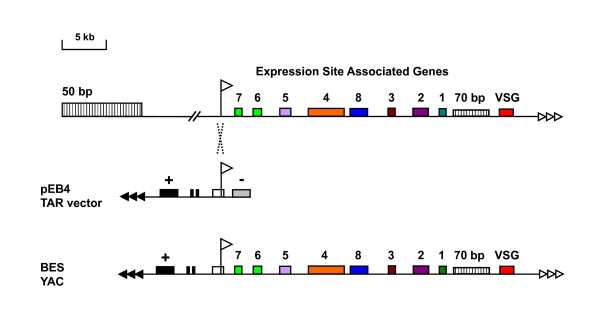
**Isolation of trypanosome BESs in yeast using Transformation Associated Recombination (TAR) cloning as described in**[** 34**]. A schematic of a typical BES is shown above with the promoter indicated with a flag, and different expression site associated genes (ESAGs) indicated with coloured boxes. Characteristic repeat arrays either upstream of the BES promoter (50 bp repeats) or adjacent to the telomeric *VSG *gene (70 bp repeats) are indicated with vertically hatched boxes. Trypanosome telomere repeats are indicated with white arrows. The linearised pEB4 TAR vector is shown below with yeast telomere repeats (black triangles) as well as a positive (+) and a negative (-) selectable marker flanking a BES promoter containing fragment. Recombination between the vector and genomic DNA on the BES promoter fragment results in a yeast artificial chromosome (YAC) which can be stably maintained.

We cloned repertoires of BES containing telomeres from three trypanosome subspecies: *T. b. gambiense *DAL 972 (genome strain currently being sequenced by the Sanger Research Institute), *T. b. brucei *EATRO 2340 and *T. equiperdum *STIB 818. *T. b. brucei *EATRO 2340 was originally thought to be a *T. b*. *rhodesiense *subspecies [35]. However, as we did not find evidence for the presence of *SRA *which is considered diagnostic for *T. b. rhodesiense *[12,13], this strain was tentatively redesignated to be a *T. b. brucei *(see Additional file 1 and Materials). The BES containing telomeres were isolated in yeast using a method relying on Transformation Associated Recombination (TAR) cloning [34,36]. Linearised yeast TAR vector pEB4, containing a BES promoter fragment and a yeast telomere, was cotransformed into yeast spheroplasts together with total trypanosome genomic DNA (Fig. 1). Recombination between the BES promoter within the vector and similar sequences within the trypanosome genomic DNA provides the yeast vector with a second (trypanosome derived) telomere, thereby stabilising the episome as a YAC (yeast artificial chromosome).

As BES sequences are polymorphic, we typed the different BES TAR clones into different BES sets based on the promoter and *ESAG6 *sequence types as previously described for *T. b. brucei *427 [34]. For the BES promoter sequence typing, an approximately 635 bp region immediately downstream of the BES promoter (not overlapping with the target fragment used for the TAR cloning) was amplified from each TAR clone and sequenced (See Additional file 2 for BES promoter sequence alignment). For the *ESAG6 *sequence typing an approximately 760 bp region spanning the *ESAG6 *hypervariable region was amplified and sequenced from each TAR clone (see Additional file 3 for *ESAG6 *sequence alignments). The presence of sequence polymorphisms within these different BES regions allowed the typing of the different TAR clones into different BES sets.

A total of 204 *T. b. gambiense *TAR clones were sorted into 13 putative BES sets (Table 1), 208 *T. b. brucei *clones were sorted into 23 BES sets (Table 2) and 91 *T. equiperdum *clones were sorted into 16 BES sets (Table 3). Two TAR clones were chosen for each BES type and the full length *ESAG6*, *ESAG5 *and *ESAG2 *open reading frames were isolated and sequenced from both of these TAR clones (one representative clone is listed in each Table). These *ESAG*s were chosen for further analysis as they are similar enough between different BESs to allow PCR amplification and sequencing directly from the yeast TAR clones.

**Table 1:** *T. brucei gambiense *DAL 972 BES TAR clone library.

**BES**	**Prom. type**	**ESAG6 type**		**No. TAR clones**	**Represent. TAR clone**	**ESAG5 type**		**ESAG2 type**	
					
		**DNA**	**Protein**			**DNA**	**Protein**	**DNA**	**Protein**
**1**	1	1	1	18	A41	1	1	1	1
**2**	2	2^a^	-	1	2.2–37	2	2	-^b^	-
**3**	2	3	1	27	A120	2	2	-^b^	-
**4**	3	4	1	15	D18	3	3	2^c^	1
**5**	4	5	1	17	B49	4	3	3	2
**6**	5	5	1	35	D30	4	3	4	3
**7**	6	6	2	22	C10	4	3	5	4
**8**	7	7	3	8	D102	5	4	6	5
**9**	8	8	4	3	2.1–9	4	3	7	6
**10**	8	9	5	24	B46	6	5	8	6
**11**	9	10	6	16	C49	4	3	9	7
**12**	10	11	7	17	98	7	6	10	8
**13**	11	11	7	1	A121	7	6	10	8

**Total**	**11**	**11**	**7**	**204**		**7**	**6**	**10**	**8**

Overview of *T. b. gambiense *DAL 972 TAR clone library. A total of 204 *T. b. gambiense *TAR clones were isolated in yeast and typed into 13 different BES sets after sequencing over the promoter and *ESAG6 *regions. The different BES promoter and ESAG6 sequence types (either at the DNA or protein level) are indicated with numbers. Two TAR clones were chosen from each BES type (the name of one representative clone is indicated in the chart), and the full length *ESAG6*, *ESAG5 *and *ESAG2 *ORFs were isolated and sequenced from both of these two clones. The sequence types of the ESAG5 and ESAG2 are indicated.

^a^Partial sequence.

^b^Presence not detected by PCR. See Materials and Methods for diagnostic primers that were used for each *ESAG*.

^c^Approximately 300 bp is single stranded sequence.

**Table 2:** *T. brucei brucei *EATRO 2340 BES TAR clone library.

**BES**	**Prom. type**	**ESAG6 type**		**No. TAR clones**	**Represent. TAR clone**	**ESAG5 type**		**ESAG2 type**	
					
		**DNA**	**Protein**			**DNA**	**Protein**	**DNA**	**Protein**
**1**	1	1	1	11	F44	1	1	1	1
**2**	2	2	2	9	E42	2	2	2	2
**3**	3	3	3	9	G37	3	3	3	3
**4**	4	4^a^	4^a^	3	E16	4	4	-^b^	-
**5**	5	5	5	19	G39	5^c^	-	-^b^	-
**6**	5	6	6	5	K13	-^d^	-	-^b^	-
**7**	6	7	7	1	V6	6	5	4	4
**8**	7	8	8	8	E51	7	6	5	5
**9**	8	9	9	7	S32	8	7	6^e^	6^e^
**10**	9	10	10	4	U19	9	8	7	7
**11**	9	11 and 12^f^	11 and 12^f^	1	S43	10	9	8	8
**12**	10	13	13	16	E23	11	10	9	9
**13**	11	14	14	8	J12	12	11	10	10
**14**	11	15	15	9	F36	13	12	11	11
**15**	12	16	16	17	E12	14	13	12	12
**16**	13	17	17	2	R23	10	9	8	8
**17**	13	11	11	2	E40	10	9	8	8
**18**	13	18	18	4	J4	10	9	13^e^	13^e^
**19**	14	19	19	13	E19	15	14	14	14
**20**	15	20	20	12	F35	16	15	15	15
**21**	16	21	21	16	E48	17	16	16	16
**22**	17	22	22	31	F27	18	17	17	17
**23**	18	11	11	1	E44	-^b^	-	-^b^	-

**Total**	**18**	**22**	**22**	**208**		**18**	**17**	**17**	**17**

Overview of *T. b. brucei *EATRO 2340 TAR clone library. A total of 208 TAR clones were isolated and typed into 23 different BES sets. The sequence typing proceeded further as described for Table 1. As before, two TAR clones were chosen for each BES type (the name of one clone is indicated in the chart), and the full length *ESAG6*, *ESAG5 *and *ESAG2 *ORFs were isolated and sequenced from both of these two clones.

^a^First 1000 bp only.

^b^Presence not detected by PCR. See Materials and Methods for diagnostic primers that were used for each *ESAG*.

^c^Pseudogene.

^d^Present but not sequenced.

^e^One clone.

^f^Two *ESAG6 *in one BES, both assembled.

**Table 3:** *T. equiperdum *STIB 818 BES TAR clone library.

**BES**	**Prom. type**	**ESAG6 type**		**No. TAR clones**	**Represent. TAR clone**	**ESAG5 type**		**ESAG2 type**	
					
		**DNA**	**Protein**			**DNA**	**Protein**	**DNA**	**Protein**
**1**	1	1^a^	-	9	10	1^b^	-	-^c^	-
**2**	2	2	1	1	3	-^c^	-	-^c^	-
**3**	3	3^d^	-	11	D27	2	1	1	1
**4**	4	4^d^	-	2	25	3	2	-^c^	-
**5**	5	5^b^	-	2	17	-^c^	-	2	2
**6**	6	6	2	8	14	4	3	3	3
**7**	6	7^e^	-	7	D44	5	4	4	4
**8**	6	8^b^	-	3	16	6	5	2	2
**9**	7	9	3	7	7	7	6	1	1
**10**	8	9	3	9	1	8	7	1	1
**11**	9	10	4	10	53	9^b^	-	-^c^	-
**12**	10	11	5	8	C23	9^b^	-	2	2
**13**	10	12	6	1	K13	9^b^	-	2	2
**14**	11	13	7	4	G47	6	5	-^c^	-
**15**	11	-^f^	**-**	8	E6	10	8	2	2
**16**	12^g^	-^f^	-	1	J46	11	9	-^c^	-

**Total**	**12**	**13**	**7^h^**	**91**		**11**	**9**	**4**	**4**

Overview of *T. equiperdum *STIB 818 TAR clone library. A total of 91 TAR clones were isolated and typed into 16 different BES sets. The sequence typing was performed as described for Table 1. As done previously, two TAR clones were chosen for each BES type (the name of one clone is indicated in the chart), and the full length *ESAG6*, *ESAG5 *and *ESAG2 *ORFs were isolated and sequenced from both of these two clones.

^a^Double sequence in the centre indicating two sequence types.

^b^Pseudogene.

^c^Presence not detected by PCR. See Materials and Methods for diagnostic primers that were used for each *ESAG*.

^d^Partial sequence.

^e^Double sequence indicating two sequence types within the same BES.

^f ^Sequence failed.

^g^Anti-sense sequence strand only.

^h^Probably an underestimate.

We have previously isolated a repertoire of 17 BES types from *T. b. brucei *427 from a total of 182 TAR clones [34]. This resulted in us cloning all ten BESs known to be functionally active *in vitro *in *T. b. brucei *427 in our laboratory [37]. Given the number of TAR clones analysed here (204 for *T. b. gambiense *and 208 for *T. b. brucei *EATRO 2340) it is likely that we have isolated complete or nearly complete BES sets from these two trypanosome subspecies.

### BES repertoire size and diversity

The overall nucleotide diversity of each repertoire, summarized in Table 4, shows both the percentage of nucleotides in different loci that are polymorphic (% S) as well as the mean pairwise diversity (π). Similar measures of amino acid diversity are summarized in Table 5. Two patterns are apparent: First, in general the nucleotide and amino acid diversity of each BES locus repertoire is greatest in *T. b. brucei*, intermediate in *T. equiperdum*, and least in *T. b. gambiense*. Since this is true of both measures of diversity, these observations cannot be explained by the presence of a few exceptionally divergent sequences or a few exceptionally polymorphic sites in the *T. b. brucei *repertoires. Rather, there are broad-based, sequence-wide differences in the diversity of these repertoires. Second, despite having a smaller number of sequence types identified, the *ESAG2 *repertoire in each subspecies is more polymorphic than the repertoires of either the BES promoter region, *ESAG6 *or *ESAG5*.

**Table 4:** BES nucleotide diversity.

**Locus**	**Strain**	**N° seqs**	**Length**	**Polymorphic sites % S**	**Average nt. Diversity π**	**N° rec. break points**	**Tract length**
**Promoter**	T. b. g.	13	621	5.6	0.015	1	311
	T. b. b.	23		24.3	0.058	2	207
	T. eq.	15		13.8	0.046	2	207
***ESAG6***	T. b. g.	12	1197	6.0	0.012	1	599
	T. b. b.	23		17.3	0.051	5	200
	T. eq.	10		8.4	0.030	3	299
***ESAG5***	T. b. g.	13	1429	1.5	0.002	0	1429
	T. b. b.	21		20.0	0.055	7	179
	T. eq.	14		14.6	0.061	7	179
***ESAG2***	T. b. g.	11	1291	15.3	0.047	7	161
	T. b. b.	19		28.7	0.101	9	129
	T. eq.	10		16.9	0.044	9	129

Nucleotide diversity and recombination in BES sequences from three trypanosomatid protozoa. The BES loci studied are indicated on the left. The strains are *Trypanosoma brucei gambiense *DAL 972 (T.b.g.), *T. b. brucei *EATRO 2340 (T.b.b.) and *T. equiperdum *STIB 818 (T. eq.). N° seqs. = number of nucleotide sequences in alignment; L = number of nucleotides in gap-stripped alignment; %S = percentage of polymorphic sites; π = average pairwise nucleotide diversity; N° rec. break points and tract length = number of recombination breakpoints and mean tract length inferred by GARD analysis.

**Table 5:** BES amino acid diversity.

**Locus**	**Strain**	**N° seqs**	**Codons**	**Polymorphic Sites % S**	**Average a.a. diversity π**	**dN/dS ratio ω > 2**	**Average dN/dS ω (avg)**
**ESAG6**	T. b. g.	12	401	9.2	0.018	15	3.40
	T. b. b.	23		24.7	0.079	24	3.20
	T. eq.	8		9.2	0.040	38	2.15
**ESAG5**	T. b. g.	13	469	2.8	0.005	0	n.a.
	T. b. b.	19		27.5	0.087	48	3.75
	T. eq.	10		19.4	0.087	49	2.29
**ESAG2**	T. b. g.	11	424	19.1	0.059	15	3.38
	T. b. b.	19		37.0	0.130	11	3.98
	T. eq.	10		19.6	0.053	11	4.39

Amino acid diversity and selection in ESAG sequences from three trypanosomatid protozoa. The BES loci studied are indicated on the left. The strains are *Trypanosoma brucei gambiense *DAL 972 (T.b.g.), *T. b. brucei *EATRO 2340 (T.b.r.) and *T. equiperdum *STIB 818 (T. eq.). N° seqs. = number of protein sequences in alignment; codons = number of codons in gap-stripped alignment; % S = percentage of polymorphic sites; π = average pairwise amino acid diversity; dN/dS ratio ω > 2 = number of amino acid residues with dN/dS ratio (ω) estimated to be greater than 2 indicating evidence for positive selection; Average dN/dS ω (avg.) = average ω estimate over residues with ω > 2.

**Figure 2:**
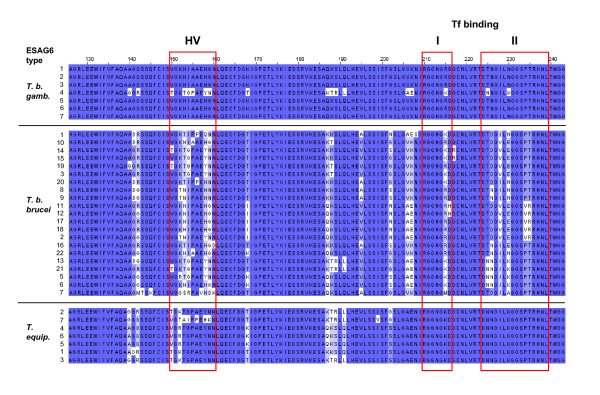
**Sequence alignment over a particularly polymorphic region of ESAG6 shows that *T. b. gambiense *ESAG6 types are significantly less diverse over the hypervariable region than ESAG6 sequences from *T. b. brucei *and *T. equiperdum*.** The ESAG6 protein sequence types as listed in Tables 1-3 are indicated on the left with amino acid residue position above. The ESAG6 hypervariable region (HV) as described in [38,40] as well as boxes I and II of the ESAG6 transferrin binding site (Tf binding) as described in [39] are indicated with red boxes. Residues that are the most dissimilar to the consensus within a given trypanosome subspecies are highlighted in white. Residues that are completely conserved within a given subspecies are indicated in dark blue, while residues with intermediate degrees of sequence conservation are highlighted with intermediate shades of blue.

Sequence repertoires of loci from *T. b. gambiense *were consistently the least genetically diverse. In *T. b. brucei *the transferrin receptor subunit ESAG6 has been shown to be particularly polymorphic over a hypervariable domain, as well as over a domain implicated in binding polymorphic host transferrins [38-40]. It has been proposed that ESAG6 sequence polymorphisms allow the trypanosome to express receptors with differing affinities for the polymorphic transferrin molecules from different species of mammalian host [27,28]. Sequence alignment of these polymorphic regions of ESAG6 shows that ESAG6 sequences from *T. b. brucei *EATRO 2340 have comparable levels of amino acid diversity to that found in other *T. brucei *species [40] (Fig. 2). However, as shown previously, ESAG6 sequences from *T. equiperdum*, while diverse in the ESAG6 hypervariable region, have little amino acid diversity within the transferrin binding region (Fig. 2)[40,41]. Here, we show that there is very little amino acid diversity in ESAG6 in *T. b. gambiense *DAL 972, even over these normally quite polymorphic stretches of the protein (Fig. 2). Surprisingly, six out of the seven *T. b. gambiense *ESAG6 protein types identified were identical over this polymorphic stretch.

### Phylogenetic analyses of BES sequence families

**Figure 3:**
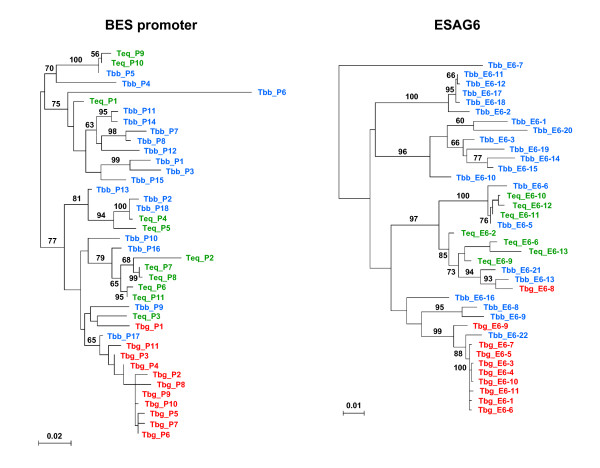
**Maximum likelihood phylogenetic trees show that BES promoter and *ESAG6 *sequences from *T. b. gambiense *972 (Tbg) cluster separately from those from *T. b. brucei *EATRO 2340 (Tbb) and *T. equiperdum *STIB 818 (Teq).** The DNA sequence types are as listed in Tables 1-3. Sequence accession numbers are listed in the Materials and Methods. Alignments of the BES promoter sequences used are shown in the supplementary material (Additional file 2). Bootstrap support values are shown for key nodes only.

**Figure 4:**
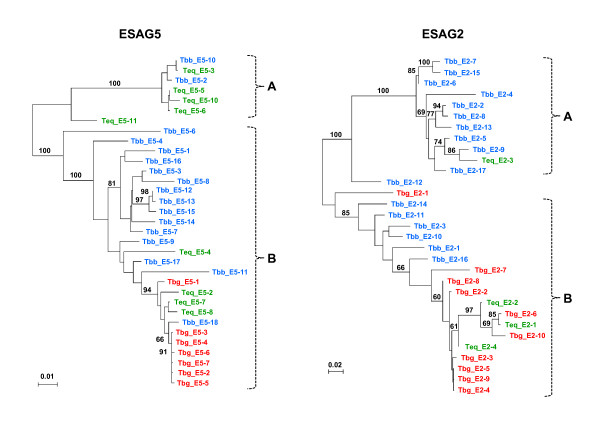
**Maximum likelihood phylogenetic trees of *ESAG5 *and *ESAG2 *show that *ESAG5 *sequences from *T. b. gambiense *972 (Tbg) cluster separately from those from *T. b. brucei *EATRO 2340 (Tbb) and *T. equiperdum *STIB 818 (Teq)**. Most of the *T. b. brucei ESAG2 *sequences also appear to cluster separately. The *ESAG *DNA sequence types are as listed in Tables 1-3. Sequence accession numbers are listed in the Materials and Methods. Bootstrap support values are shown for key nodes only. Type A and Type B sequences referred to in the text are indicated with brackets.

Phylogenetic analyses (Fig. 3 and Fig. 4) show that the relationships between the BES repertoires in the different trypanosome subspecies differ between loci. Within the BES promoter region, as well as at *ESAG6 *and *ESAG5*, most of the sequences obtained from *T. b. gambiense *belong to nearly monophyletic groups (*gambiense*-like) with little nucleotide or amino acid diversity. Nonetheless, at each locus, reciprocal monophyly between the *T. b. gambiense *repertoire and homologous sequences in the other two subspecies is violated, both by the existence of *T. b. gambiense *sequences which fall outside the main *gambiense*-like groups (e.g. Tbg_P1 for the promoter region; Tbg_E6-8 for *ESAG6*) and by the existence of *T. b. brucei *and *T. equiperdum *sequences which lie within these groups (e.g. Tbb_P17 for the promoter region and Teq_E5-8 for *ESAG5*).

In contrast, the *T. b. brucei *and *T. equiperdum *sequences are both collectively more diverse and are mutually paraphyletic. Not only are there several groups containing sequences from both subspecies, but there are several *T. b. brucei *and *T. equiperdum *homologs which are nearly identical (e.g. promoter region: Tbb_P-5 and Teq_P-10, *ESAG6*: Tbb_E6-5 and Teq_E6-11, and *ESAG5*: Tbb_E5-10 and Teq_E5-3) (Fig. 3 and Fig. 4). Furthermore, to the extent that there is local, strain-based structure outside of the *gambiense*-like groups, this mainly consists of groups of *T. b. brucei *sequences which have no closely related sequences in the *T. equiperdum *genome (e.g., the group of *ESAG6 *sequences including Tbb_E6-11, Tbb_E6-10 and 10 other *T. b. brucei *sequences).

The *ESAG2 *phylogenetic tree reveals a rather different set of relationships between the sequences from the different subspecies (Fig. 4). Apart from Tbg_E2-1 and Teq_E2-3, all of the *T. b. gambiense *and *T. equiperdum ESAG2 *sequences belong to a single group which is itself only partially strain-structured. Indeed, one pair of *T. b. gambiense *and *T. equiperdum ESAG2 *homologs is nearly identical (Tbg_E2-6 and Teq_E2-1). Furthermore, most *ESAG2 *sequences can be divided into two relatively divergent subfamilies, one consisting almost exclusively of *T. b. brucei *sequences apart from Teq_E2-3 (*ESAG2 *type A sequences), and the other containing almost all of the *T. b. gambiense *and *T. equiperdum *sequences as well as six *T. b. brucei *sequences (*ESAG2 *type B sequences). Inspection of the ESAG2 amino acid sequence alignments (see Additional file 4) reveals that the ESAG2 consensus type A and type B sequences differ at 123 of 474 residues (25.9%). This alignment also suggests that the most basally-branching ESAG2 sequences may be recombinants between type A and type B sequences. For example, although the Tbb_E2-12 ESAG2 sequence has a type A N-terminal domain (residues 1–54), the remaining residues predominantly agree with the type B consensus sequence. Likewise, the Tbg_E2-1 ESAG2 sequence is most similar to the type A consensus, but contains a tract of type B amino acids between residues 302–359.

The ESAG5 amino acid sequences can also be partitioned into two subfamilies; although the differentiation is less pronounced, with consensus sequences which differ at 72 of 480 residues (15%) (see Additional file 5). The group of six relatively homogeneous *T. equiperdum *and *T. b. brucei *sequences in the *ESAG5 *phylogenetic tree constitute the *ESAG5 *type A subfamily, while the remaining sequences constitute the type B subfamily. As with *ESAG2*, the most basally-branching *ESAG5 *sequence appears to be a type A-type B recombinant. Although this Teq_E5-11 *ESAG5 *sequence is more closely related to the *ESAG5 *type A subfamily, it includes several tracts of type B consensus residues (e.g., residues 59–106).

### Evidence of recombination within BES loci

Having observed putatively recombinant sequences in both the *ESAG2 *and *ESAG5 *families, we used a phylogenetic method to rigorously test each of the sequences from the four BES loci in the three trypanosome subspecies for evidence of recombination. The number of recombination breakpoints was inferred using GARD analysis (Table 4). With the exception of the *T. b. gambiense ESAG5 *alignment, the GARD analysis detected at least one breakpoint in each alignment, with the density of breakpoints increasing towards the telomere. For example, in the *T. b. brucei *sequence alignments, adjacent breakpoints are separated on average by 207 bp in the promoter region, 200 bp in *ESAG6*, 179 bp in *ESAG5*, and 129 bp in *ESAG2*. While comparable numbers of breakpoints are detected in the *T. b. brucei *and *T. equiperdum *alignments, fewer are present in the corresponding *T. b. gambiense *alignments (Table 4).

Although these analyses suggest that allelic recombination rates within the trypanosome BES repertoire might vary between loci and between subspecies, it is important to point out that both trends, that of increasing recombination towards the telomeres, and of reduced recombination within *T. b. gambiense*, are confounded by subspecies and locus-specific differences in genetic variation. Indeed, the mean number of sites between adjacent breakpoints inferred by GARD is significantly negatively correlated with both measures of nucleotide diversity reported in Table 4 (tract length ~%S: r^2 ^= 0.466, P = 0.001; tract length ~π: r^2 ^= 0.462, P = 0.001). This correlation reflects the fact that the power of GARD to infer recombination breakpoints is limited by the amount of polymorphism in the data. In particular, it is possible that the homogeneity of the *T. b. gambiense ESAG5 *repertoire is due to extensive gene conversion.

### Adaptive evolution and amino acid diversity in ESAG repertoires

In order to characterize the role of selection in the molecular evolution of the *ESAG *repertoires of *T. b. brucei*, *T. b. gambiense*, and *T. equiperdum*, we estimated the distribution of the relative ratio of non-synonymous-to-synonymous substitutions (dN/dS ratio or ω) in ESAG6, ESAG5 and ESAG2 from the three trypanosome subspecies. Maximum likelihood (ML) estimates of the parameters of the nearly neutral model M1a and the selection model M2a, as well as the p-values of the likelihood ratio tests comparing the selection model with the nearly neutral model are shown in the table in Additional file 6. These analyses provide evidence for adaptive evolution within the ESAG6, ESAG5, and ESAG2 repertoires of *T. b. brucei *and *T. equiperdum*, and within the ESAG2 repertoire of *T. b. gambiense*. In contrast, there is no statistically significant evidence for diversifying selection within the ESAG6 and ESAG5 repertoires of *T. b. gambiense*.

Because inference of subspecies-specific selection is complicated by the lack of reciprocal monophyly between the homologous ESAG repertoires of the different strains, the contrasting patterns seen in the ESAG6 and ESAG5 repertoires between *T. b. gambiense *and the other two subspecies are particularly striking (Fig. 3 and 4). On the one hand, the paraphyly of the *T. b. gambiense *and *T. equiperdum *ESAG2 repertoires could explain why this locus does not follow the same pattern (e.g. the amino acid replacements in ESAG2 could predate the origin of *T. b. gambiense*). Alternatively, diversifying selection on the ESAG2 sequences in the *T. b. gambiense *genome may have prevented the extensive homogenisation that has occurred within the ESAG5 and ESAG6 families. The methods employed here cannot distinguish between these possibilities.

In order to look in more detail at the patterns of molecular evolution within individual ESAGs, relative nonsynonymous substitution rates at individual codons were estimated using the prior distributions determined by the ML estimates of the model M2a parameters. The estimated rates are listed in the table in Additional file 7 and plotted against residue number in Additional file 8 (details in the table in Additional file 7). Consistent with earlier surveys of ESAG6 diversity [40,41], several residues in the hypervariable region of ESAG6 appear to be under diversifying selection in all three trypanosome subspecies. Adaptive evolution within the two transferrin-binding regions (box 1 and box 2) is evident in the *T. b. brucei *repertoire but not in *T. b. gambiense *or in *T. equiperdum*. The *T. equiperdum *data is consistent with data published in [40,41]. These functional elements of ESAG6 are annotated in the table in Additional file 7.

One unexpected finding is that several residues within the signal peptide of each ESAG appear to be under diversifying selection, despite the fact that these regions are cleaved from the nascent protein [42]. There are three such residues in ESAG6, five in ESAG5, and four in ESAG2. However, there are many other residues under diversifying selection in these ESAGs which have no known structural or functional association. Several of these residues are inferred to be evolving adaptively in all three subspecies, including residues 186 in ESAG6 and 81 and 434 in ESAG2.

Table 5 shows the numbers of amino acid residues within each alignment at which the non-synonymous substitution rate is more than twice as large as the synonymous substitution rate (ω > 2). Also shown are the average estimates of ω at such residues, which is a measure of the strength of diversifying selection. As can be seen in Additional file 8, there are notable differences in the patterns of adaptive evolution in the three ESAG families. While the absolute number and density of diversifying residues in the *T. b. brucei *and *T. equiperdum *repertoires is greatest in ESAG5 and least in ESAG2, the apparent strength of diversifying selection at adaptively evolving residues is greatest in ESAG2.

Curiously, almost the opposite pattern is evident in the *T. b. gambiense *ESAG repertoires, with comparable numbers of diversifying codons in ESAG6 and ESAG2, but almost no amino acid variation in the ESAG5 sequences. It is also surprising that the smaller and less diverse *T. equiperdum *ESAG repertoires contain at least as many apparently adaptively-evolving sites as the corresponding *T. b. brucei *repertoires. This difference is most pronounced in ESAG6, where there are about 50% more diversifying residues in *T. equiperdum *than in *T. b. brucei*, despite the apparently stronger selection at such residues in the latter subspecies due to the larger size of its host range. Of course, as remarked above, all inter-strain comparisons are confounded by the paraphyletic relationships between these repertoires.

### BES sequence diversity and trypanosome biology

The three trypanosome subspecies analysed here are highly similar at the DNA sequence level, yet cause diseases with very different pathologies. Whereas *T. b. gambiense *infection produces a chronic form of human disease, *T. b. brucei *is not human infective. Similarly, these trypanosome subspecies have different susceptibilities to drugs, whereby *T. b. gambiense *is very susceptible to the drug DFMO unlike *T. b. brucei *[43]. *T. equiperdum*, although closely related to *T. b. brucei *[14], infects different environments within the mammalian host. Rather than multiplying extracellularly in the mammalian bloodstream like *T. b. brucei *and *T. b. gambiense*, *T. equiperdum *is a tissue parasite primarily localised in the mucous membranes of the urogenital tract, and is rarely observed in the bloodstream [15]. The challenge will come in trying to understand how the limited genetic differences between these subspecies translate into their very different pathologies.

If ESAGs mediate host-parasite interactions, one could expect the diversity of individual BES repertoires to be positively correlated with host range size. This is because trypanosomes infecting multiple host species may be under selection to evolve and then maintain distinct ESAG alleles that are adapted to the different environments encountered in different species of mammalian host. In fact, our data are not completely consistent with this prediction. Of the three trypanosomes analysed here, host range is greatest in *T. b. brucei *[10], intermediate in *T. b. gambiense *[11], and smallest in *T. equiperdum *[15]. However, although ESAG diversity is consistently higher in *T. b. brucei *than in the other two subspecies, the *T. b. gambiense *BES repertoires are actually less diverse than those of *T. equiperdum*. Likewise, although we find evidence for adaptive evolution in all three of the ESAG repertoires sequenced in *T. b. brucei *and *T. equiperdum*, only the ESAG2 repertoire of *T. b. gambiense *appears to be under diversifying selection. In addition, we find no correlation between the number of BESs found in a particular trypanosome subspecies and the host range size. Lastly, we find evidence for adaptive evolution of the ESAG6 repertoire of *T. equiperdum*, where it would be expected to be minimal or absent due to the restricted size of the host range of this trypanosome.

There are several reasons why ESAG diversity and host range size might not be positively correlated. The hypothesis that diverse ESAG repertoires could facilitate trypanosome infection of diverse mammalian hosts was first proposed for ESAG6 [27], where different sequence polymorphisms affect binding affinity of the transferrin receptor to variable transferrin molecules [39]. This proposal has subsequently proved controversial. It has more recently been argued that *in vivo *concentrations of transferrin are high enough that expression of even low-affinity transferrin receptors is more than adequate to provide a trypanosome with sufficient transferrin in a range of different mammalian hosts [44,45]. These results would imply that ESAG6 sequence diversity within a given trypanosome subspecies need not be correlated with size of host range.

Alternatively, even if host range is an important determinant of the selection pressures on ESAG repertoires, observed levels of ESAG diversity within individual genomes are probably influenced by a variety of processes that can create or remove variation. Sequence variation can be both generated and removed through DNA recombination. One possibility is that *T. b. gambiense *has unusually high rates of telomeric DNA rearrangements, resulting in sequence homogenisation of *ESAG *repertoires through allelic gene conversion or gene duplication and deletion. Another confounding factor is that genetic exchange between ESAGs and their genome-internal paralogs could influence the polymorphism observed within the BES repertoires. However, we can not easily reconcile why high rates of gene conversion in *T. b. gambiense *would lead to the relative homogenisation of the *ESAG5 *gene family, but not of the ESAG2 repertoire. We will only be able to understand the selection pressures maintaining *ESAG *sequence diversity within a given trypanosome subspecies once we know more about the function of these various *ESAG *gene families.

Another factor that might influence the generation and maintenance of ESAG sequence diversity is the degree of genetic exchange that any given trypanosome subspecies undergoes. Different *T. b. brucei *and *T. b. rhodesiense *strains have been shown to undergo sex in the laboratory after passage through tsetse flies [46-48], although the frequency with which this occurs in the field is unclear [49]. This genetic exchange could allow the continuous flow of new ESAG sequences into a strain, facilitating the accumulation of genetic diversity. However in the Type I group of *T. b. gambiense *trypanosomes including *T. b. gambiense *DAL972 analysed here, there is no evidence for significant genetic exchange occurring [50]. Population structure analyses indicate that *T. b. gambiense *Type I populations form a highly homogeneous group of trypanosomes which appear to have expanded clonally [51,52], and could have lost the ability to undergo meiosis [48]. Lack of or extremely infrequent genetic exchange together with significant rates of telomeric gene conversion would be expected to facilitate the homogenisation of repetitive gene families in the absence of a strong selection pressure maintaining diversity

Why then are the ESAG sequences from *T. b. gambiense *more homogeneous than those from *T. equiperdum*, which could have lost the opportunity to undergo meiosis altogether when it ceased to be transmitted via tsetse flies? The diversity within these ESAG repertoires is presumably not at equilibrium. *T. equiperdum *is likely to have arisen from *T. b. brucei *relatively recently (as proposed in [14]), and thus may not have had time to completely lose ancestral ESAG sequence variation that is not actively being selected for in the equine host. In contrast, *T. b. gambiense *(Type I) is thought to be much more distantly related to *T. b. brucei *[52]. This is consistent with the genealogical relationships of the ESAG6, ESAG5 and BES promoter repertoires in these three subspecies, i.e., the *T. b. brucei *and *T. equiperdum *repertoires are paraphyletic, while the *T. b. gambiense *repertoires are nearly reciprocally monophyletic, but does not explain the very different relationships seen at ESAG2.

A point to remember is that BESs are dynamic groups of genes. Each of the BES repertoires cloned presents a snapshot of what is going on within a population of trypanosomes, and each repertoire will contain a subset of ESAG 'alleles' present within the given trypanosome population. Further population structure analysis will allow us to determine if what we see in these analysed subspecies is a consequence of sampling rather than due to selection or consequences of loss of parts of the life-cycle. While these preliminary analyses are not the end of the story, these BES libraries will be a useful resource for further analyses of the processes influencing genetic variation in the telomeres of African trypanosomes.

## Methods

### Trypanosome strains and DNA isolation and analysis

The genomic DNA used in the *T. b. gambiense *TAR BES telomere library produced in this study was isolated from the insect form *Trypanosoma brucei gambiense *DAL972 genome strain (received from Bill Wickstead, Keith Gull and Wendy Gibson) according to [53]. DNA from bloodstream form *T. b. brucei *EATRO 2340 was a gift of Keith Matthews (University of Edinburgh) and was isolated according to [54]. Note that in the literature this strain is referred to as *T. b. rhodesiense *EATRO 2340 [35]. However, as we did not find evidence for the presence of *SRA *which is considered diagnostic for *T. b. rhodesiense *using *SRA *specific primers (Additional file 1) [13,55,56], this strain was provisionally redesignated *T. brucei brucei *EATRO 2340. *T. equiperdum *STIB 818 (gift of Wendy Gibson, University of Bristol) [40,57,58] was amplified in mice, and after purification of trypanosomes from whole blood using DEAE columns, genomic DNA was isolated according to [54]. All animal experiments were subject to ethical review by the University of Oxford, and were conducted according to the conditions of a Home Office project licence.

### TAR cloning of *Trypanosoma sp*. BES telomeres

Transformation Associated Recombination (TAR) cloning was performed essentially according to [34] using the *S. cerevisiae *strain TYC1 as recipient (*MAT*α, *ura3-52*, *leu2Δ1*, *cyh*2^r^). The *T. brucei *BES-specific TAR vector pEB4 contains the yeast positive selectable marker *URA3*, the negative selectable marker *CYH2*, as well as a yeast centromere, origin of replication, and one yeast telomere. The *T. brucei *specific TAR target within the pEB4 vector is a 560 bp fragment with a region of the *T. brucei *BES core promoter that is particularly conserved over a range of different *T. brucei *strains [34]. Recombination in yeast between the pEB4 TAR vector linearised with *Cla*I and the cotransfected *T. brucei *genomic DNA, results in the production of stable "half-YAC" containing a *T. brucei *BES as well as one yeast telomere and one trypanosome telomere.

Yeast colonies were screened for the presence of *T. brucei *BES TAR clones with primers specific for *ESAG6*/*ESAG7 *corresponding to sequences located within particularly conserved regions of these genes. *ESAG6 *and *ESAG7 *are conserved elements within trypanosome BESs [26], and their presence was considered diagnostic for the presence of a *T. brucei *BES. For initial *T. b. gambiense *TAR clone screening *ESAG6/7 *screening primers ESAG7a (sense and anti-sense) were used (sequences in [34]). For screening the rest of the *T. b. gambiense *TAR clones and all of the *T. b. brucei *and *T. equiperdum *TAR clones, screening primers ESAG6/7-311s and ESAG6-809as were used. See the table in Additional file 9 for all primer sequences used.

### TAR clone analysis and sequencing

In most cases, positive yeast transformants were streaked out for single colonies, these were expanded on quarter petri dishes, and yeast genomic DNA was isolated [34]. PCR products were generated by amplification for 35 cycles at 94°C for 30 seconds, 50°C for 30 seconds, 72°C for 1 minute for products smaller than 1 kb, and 1 minute 30 seconds for products longer than 1 kb. Fragments were precipitated and sequenced using BigDye terminator (Applied Biosystems). Sequence analysis was performed with modifications of the procedure described in [34]. Sequences of all primers used in this study are presented in the table in Additional file 9.

TAR clones were typed into BES sets by sequencing the promoter region and an approximately 700 bp central stretch of *ESAG6*. Promoter sequence was trimmed to delete sequence corresponding to the region of the BES promoter present in the pEB4 TAR vector target fragment. Identification of sequence polymorphisms within both the BES promoter and *ESAG6 *allowed the categorisation of the different TAR clones into different sets corresponding to different BESs.

For typing of the different TAR clones using ES promoter sequence, primers ESP S1/S2 (sense and anti-sense) (sequence in [34]) were used for both the PCR and the sequencing step. The sequence was trimmed to delete the 5' region corresponding to the TAR vector target fragment. For *ESAG6 *sequence typing primers ESAG6-287s and ESAG6-1045as were used for both the PCR and the sequencing step.

After initial typing of the TAR clones into different BES sets, the full length *ESAG6*, *ESAG5 *and *ESAG2 *genes were amplified and sequenced from two different TAR clones from each BES set. The strategy followed was:

#### ESAG6

The 5' end was amplified and sequenced using ESAG6-UPSs and ESAG6-652as. The 3' end was amplified and sequenced using ESAG6-796s and ESAG6-DNas.

#### ESAG5

Initial PCR to amplify the coding region was performed with ESAG5-UPSs and ESAG5-DNas. If these failed, two separate reactions using ESAG5-240s and ESAG5-DNas and ESAG5-UPSs and ESAG5-578as were tried. The above products were sequenced with ESAG5-UPSs, ESAG5-DNas, ESAG5-240s, ESAG5-827s, ESAG5-578as and ESAG5-1007as.

#### ESAG2

Initial PCR was performed with ESAG2-UPSs and ESAG2-DN1as. If this failed, primers ESAG2-UPS2s and ESAG2-DN3as were used. The PCR product was cleaned and sequenced with ESAG2-UPS1s or ESAG2-UPS2s, ESAG2-DN1as or ESAG2-DN3as, ESAG2-371s, ESAG2-871s, ESAG2-505as, ESAG2-900as, ESAG2-970as or ESAG2-1088as.

Particular *ESAG*s were deemed absent from a given TAR clone if the amplification strategy detailed above was unsuccessful and if all additional primer pairs failed to give rise to an amplified product. For *ESAG5 *the additional primer pairs were ESAG5-1 (sense and anti-sense), ESAG5-2 (sense and anti-sense) and ESAG5-3 (sense and anti-sense) (sequences in [34]). For *ESAG2 *the additional primer pairs were ESAG2-1 (sense and anti-sense), ESAG2-2 (sense and anti-sense) and ESAG2-3 (sense and anti-sense) (sequences in [34]) and ESAG2-457s and ESAG2-900as (sequences in Additional file 9). The sequences of all primers used for PCR or sequencing are indicated in the table in Additional file 9.

The GenBank database accession numbers for sequences from *Trypanosoma brucei gambiense *DAL972 are: BES promoter types 1–11 [EU726336–EU726346], *ESAG6 *type 1 [EU726347] and *ESAG6 *types 3–11 [EU726348–EU726356], *ESAG5 *types 1–7 [EU726357–EU726363] and *ESAG2 *types 1–10 [EU726364–EU726373]. The GenBank database accession numbers for sequences from *Trypanosoma brucei brucei *EATRO 2340 are: BES promoter types 1–18 [EU726409–EU726426], *ESAG6 *types 1–22 [EU726427–EU726448], *ESAG5 *types 1–18 [EU726449–EU726466] and *ESAG2 *types 1–17 [EU726467–EU726483]. The GenBank database accession numbers for sequences from *Trypanosoma equiperdum *STIB 818 are: BES promoter types 1–11 [EU726374–EU726384], *ESAG6 *type 2 [EU726385], *ESAG6 *type 5 [EU726386], *ESAG6 *type 6 [EU726387], *ESAG6 *types 8–13 [EU726388–EU726393], *ESAG5 *types 1–11 [EU726394–EU726404] and *ESAG2 *types 1–4 [EU726405–EU726408].

### Sequence analysis

For the phylogenetic analyses sequence alignments were generated using ClustalX [59], and then manually edited and gap-stripped. Ambiguously-aligned regions were removed. Phylogenetic analysis of each alignment was then performed with PhyML [60], assuming a general time reversible (GTR) nucleotide substitution model with gamma-distributed rate variation among sites and a class of invariant sites. Rate parameters and initial trees were estimated from the data. Bootstrap values were estimated from 100 bootstrap samples. To summarize the overall diversity of these repertoires, we calculated the proportion of segregating sites (%S) and the mean pairwise diversity (π) of each of the 12 nucleotide alignments and 9 amino acid alignments.

Each alignment was tested for evidence of recombination using the likelihood-based method implemented in the program (GARD [61]; ). At each locus, the nucleotide substitution model used in the subsequent GARD analysis was chosen using the model selection tool provided with the HyPhy software package [62]; , and rate variation was modelled with a discrete distribution with up to three distinct rate classes. Analyses were run until incorporation of additional breakpoints did not lead to a decrease in the corrected Akaike information criterion (c-AIC). When the initial GARD analysis failed to converge in the time allocated, the alignment was divided into two subregions at the breakpoint with the highest c-AIC-support in the initial analysis, and each subregion was then separately analysed with GARD.

In order to determine the adaptive evolution of ESAG sequences, we used the method described in [63] to characterize the relative rates of non-synonymous and synonymous substitutions within the ESAG6, ESAG5 and ESAG2 gene families from each of the three trypanosome subspecies. This method extends existing likelihood-based approaches for detecting adaptive evolution of protein-coding sequences to recombinant sequences by allowing the genealogy to vary across the alignment while sharing the parameters of the codon substitution model between tracts [64,65]. Each ESAG family was codon-aligned (with pseudogenes excluded and ambiguously-aligned regions stripped), and the breakpoints identified by GARD analysis were adjusted by up to two bases to coincide with codon boundaries. The neighbour joining trees inferred during GARD analysis were used as estimates of the genealogies of the putative recombination tracts.

To assess the evidence for adaptive evolution, HyPhy was used to obtain maximum likelihood estimates of the branch lengths, the transition-to-transversion rate ratio (κ), and the parameters of two models of the distribution of the non-synonymous/synonymous rate ratio (ω). Under the nearly neutral model (M1a), ω is drawn from a discrete distribution with two classes, ω_0 _< 1 and ω_1 _= 1, with relative weights p_0 _and p_1_. The selection model (M2a) allows for a third category, ω_2 _> 1, of adaptively-evolving sites with relative weight p_2_. Because the neutral model is nested within the selection model (setting p2 = 0 in M2a recovers M1a), a likelihood ratio test for positive selection can be performed by comparing twice the log-likelihood difference between M1a and M2a with a χ^2^-distribution on two degrees of freedom [66]. The relative nonsynonymous substitution rates at individual codons were estimated using the empirical Bayes method described in [66]: model M2a, with the maximum likelihood estimated parameters, was taken as a prior distribution for ω, and Bayes' formula was used to calculate the posterior distribution of ω at each codon, given the sequence data. All calculations were carried out using a HyPhy batch language script written by Konrad Scheffler and included in the current distribution of the HyPhy software package.

## Authors' contributions

RY carried out most of the experimental work including most of the TAR cloning of the *T. brucei *telomere sets, and most of the sequence analysis. In addition, RY performed some of the bioinformatic analysis. JET carried out bioinformatic analysis on the data and helped write the manuscript. A.K. helped with TAR cloning, sequencing and bioinformatic analysis. MB performed TAR cloning of some of the *T. b. gambiense *telomere set. EJL helped with design of the experimental approach and data analysis. GR helped with the design and coordination of the project, helped with data analysis and drafted the manuscript. All authors have read and approved the final manuscript.

## Supplementary Material

Additional file 1 **Sup. Figure 1**. Multiplex PCR typing of trypanosome DNA to establish the presence or absence of the Serum Resistance Associated gene *SRA*. Multiplex PCR was performed using the primer sets and conditions of Picozzi et al [13]. Lanes indicate PCR reactions using no genomic DNA (lane 1) or genomic DNA from *Trypanosoma brucei brucei *427 (lane 2), *T. b. brucei *TREU 927/4 (lane 3), *T. b. gambiense *DAL 972 (lane 4), *T. equiperdum *STIB 818 (lane 5), *T. b. brucei *EATRO 2340 (lane 6), *T. b. rhodesiense *LVH 108 (lane 7) or *T. b. rhodesiense *WB58 (lane 8). PCR products amplifying the GPI-PLC gene (PLC), the SRA gene (SRA) or the SRA-like VSG (VSG SRA) are indicated on the right with arrows. A DNA ladder is on the left with sizes indicated in base pairs (bp). Click here for file

Additional file 2 **Sup. Figure 2**. Sequence alignment of BES promoter sequences analysed in this study. BES promoter sequences isolated from *T. b. gambiense *DAL 972, *T. b. brucei *EATRO 2340 or *T. equiperdum *were aligned using Vector NTI. Sequence types as indicated in Tables 1, 2, 3 are indicated on the left. Click here for file

Additional file 3 **Sup. Figure 3**. Sequence alignments of ESAG6 sequences analysed in this manuscript. Click here for file

Additional file 4 **Sup. Figure 4**. Sequence alignments of ESAG2 sequences analysed in this manuscript. Click here for file

Additional file 5 **Sup. Figure 5**. Sequence alignments of ESAG5 sequences analysed in this manuscript. Click here for file

Additional file 6 **Supplementary Table 2**. Non-synonymous substitution rates for different genes located within trypanosome BESs isolated from *Trypanosoma brucei gambiense *DAL 972, *T. b. brucei *EATRO 2340, or *T. equiperdum *STIB 818. Click here for file

Additional file 7 **Supplementary Table 3**. Table with the ratio of nonsynonymous-to-synonymous substitutions (ω) along ESAG6, ESAG5 and ESAG2. Click here for file

Additional file 8 **Sup. Figure 6**. The dN/dS ratio (ω) of ESAG6, ESAG5 or ESAG2 calculated from sequence repertoires from *T. b. gambiense*, *T. b. brucei *and *T. equiperdum *plotted against codon number. Click here for file

Additional file 9 **Supplementary Table 1**. Table of primers used. Click here for file
