# Supplementary figures and images for: Isolation and analysis of the genetic diversity of repertoires of VSG expression site containing telomeres from Trypanosoma brucei gambiense, T. b. brucei and T. equiperdum
Source: BMC Genomics. 2008 Aug 12;9:385. doi: 10.1186/1471-2164-9-385 (PMC2533676; doi:10.1186/1471-2164-9-385)

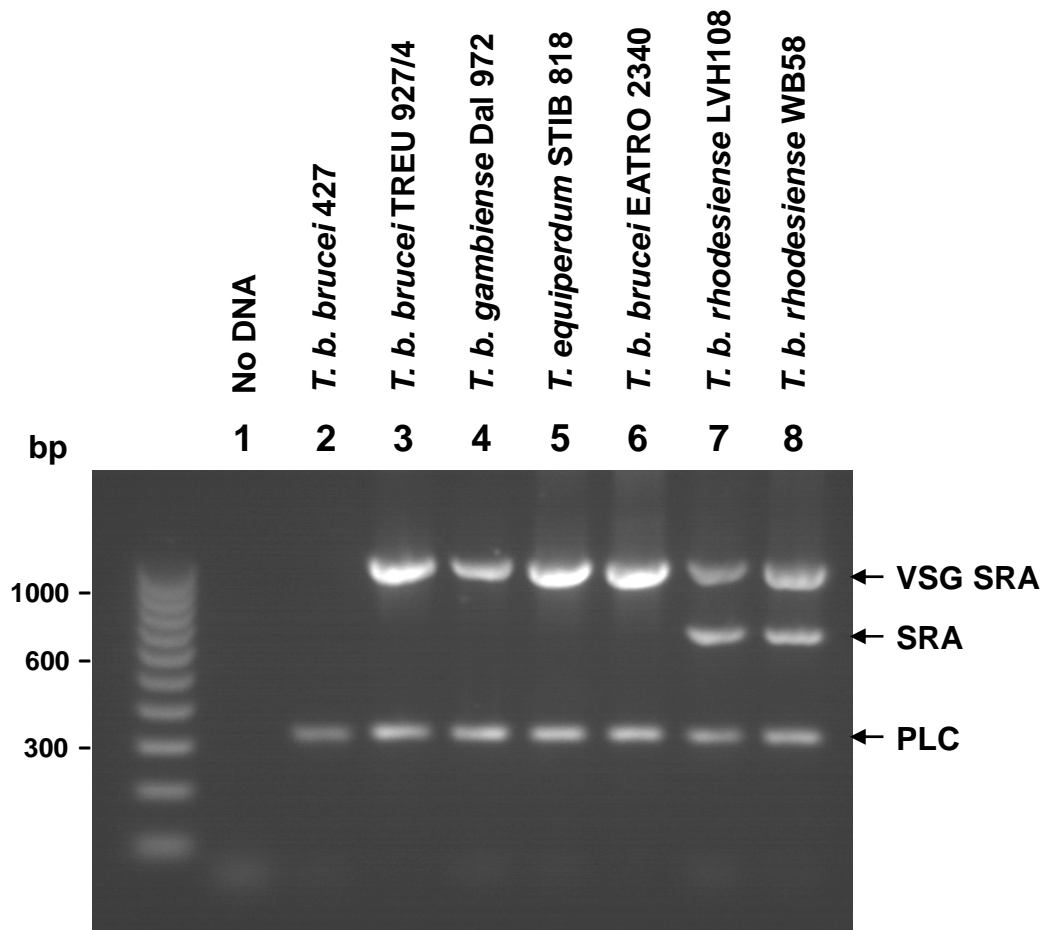

Supplement: Additional file 1 — Sup. Figure 1. Multiplex PCR typing of trypanosome DNA to establish the presence or absence of the Serum Resistance Associated gene SRA. Multiplex PCR was performed using the primer sets and conditions of Picozzi et al [13]. Lanes indicate PCR reactions using no genomic DNA (lane 1) or genomic DNA from Trypanosoma brucei brucei 427 (lane 2), T. b. brucei TREU 927/4 (lane 3), T. b. gambiense DAL 972 (lane 4), T. equiperdum STIB 818 (lane 5), T. b. brucei EATRO 2340 (lane 6), T. b. rhodesiense LVH 108 (lane 7) or T. b. rhodesiense WB58 (lane 8). PCR products amplifying the GPI-PLC gene (PLC), the SRA gene (SRA) or the SRA-like VSG (VSG SRA) are indicated on the right with arrows. A DNA ladder is on the left with sizes indicated in base pairs (bp). [file 1471-2164-9-385-S1.pdf]
