# Supplementary material for: Isolation and analysis of the genetic diversity of repertoires of VSG expression site containing telomeres from Trypanosoma brucei gambiense, T. b. brucei and T. equiperdum
Source: BMC Genomics. 2008 Aug 12;9:385. doi: 10.1186/1471-2164-9-385 (PMC2533676; doi:10.1186/1471-2164-9-385)
Supplement: Additional file 2 — Sup. Figure 2. Sequence alignment of BES promoter sequences analysed in this study. BES promoter sequences isolated from T. b. gambiense DAL 972, T. b. brucei EATRO 2340 or T. equiperdum were aligned using Vector NTI. Sequence types as indicated in Tables 1, 2, 3 are indicated on the left. [file 1471-2164-9-385-S2.pdf]

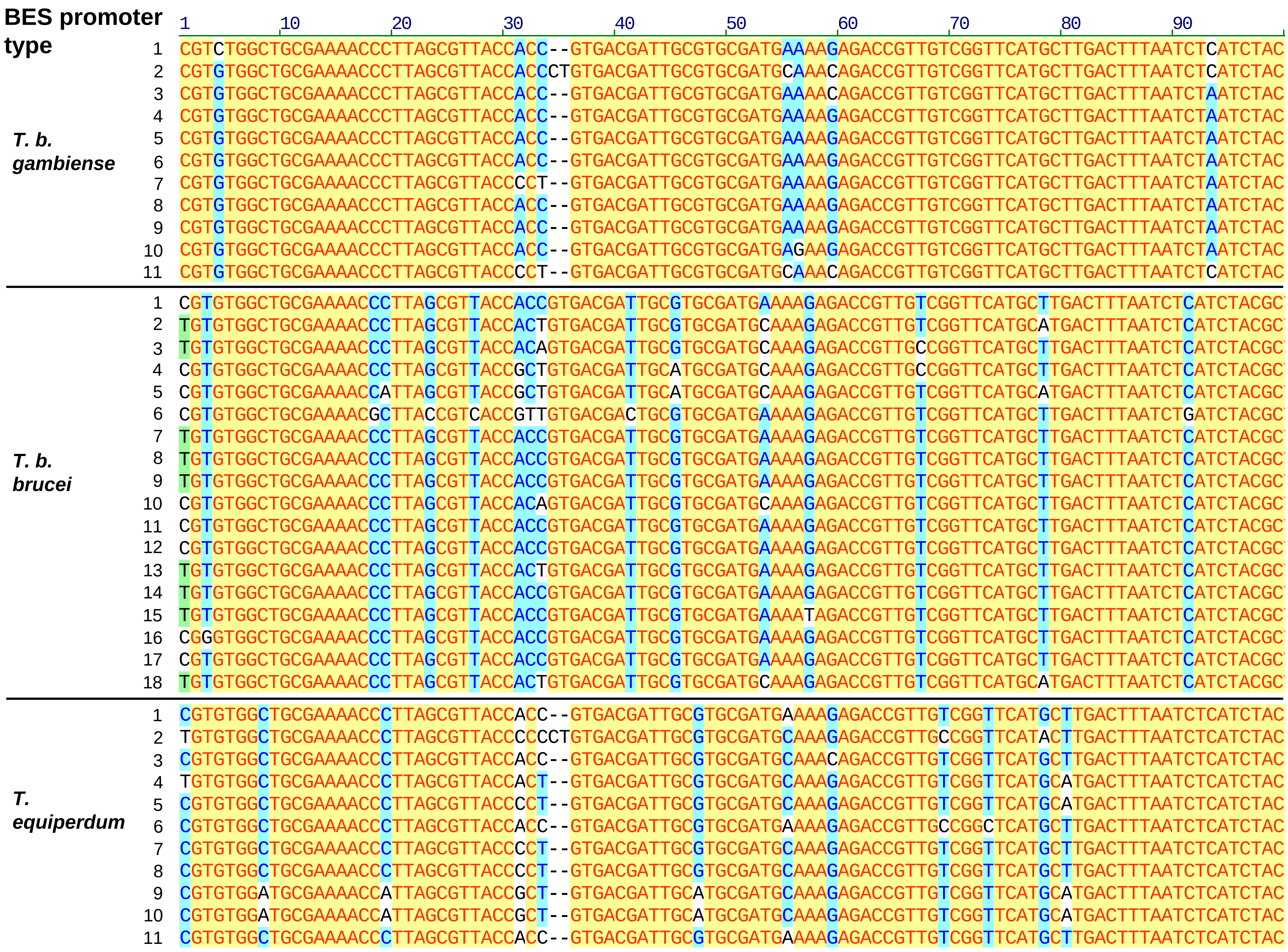
BES promoter type
1 10 20 30 40 50 60 70 80 90
T. b. gambiense
1 CGTCTGGCTGCGAAAACCCTTAGCGTTACCACC--GTGACGATTGCGTGCGATGAAAAGAGACCGTTGTCGGTTCATGCTTGACTTTAATCTCATCTAC
2 CGTGTGGCTGCGAAAACCCTTAGCGTTACCACCCTGTGACGATTGCGTGCGATGCAAACAGACCGTTGTCGGTTCATGCTTGACTTTAATCTCATCTAC
3 CGTGTGGCTGCGAAAACCCTTAGCGTTACCACC--GTGACGATTGCGTGCGATGAAAACAGACCGTTGTCGGTTCATGCTTGACTTTAATCTAATCTAC
4 CGTGTGGCTGCGAAAACCCTTAGCGTTACCACC--GTGACGATTGCGTGCGATGAAAAGAGACCGTTGTCGGTTCATGCTTGACTTTAATCTAATCTAC
5 CGTGTGGCTGCGAAAACCCTTAGCGTTACCACC--GTGACGATTGCGTGCGATGAAAAGAGACCGTTGTCGGTTCATGCTTGACTTTAATCTAATCTAC
6 CGTGTGGCTGCGAAAACCCTTAGCGTTACCACC--GTGACGATTGCGTGCGATGAAAAGAGACCGTTGTCGGTTCATGCTTGACTTTAATCTAATCTAC
7 CGTGTGGCTGCGAAAACCCTTAGCGTTACCCCT--GTGACGATTGCGTGCGATGAAAAGAGACCGTTGTCGGTTCATGCTTGACTTTAATCTAATCTAC
8 CGTGTGGCTGCGAAAACCCTTAGCGTTACCACC--GTGACGATTGCGTGCGATGAAAAGAGACCGTTGTCGGTTCATGCTTGACTTTAATCTAATCTAC
9 CGTGTGGCTGCGAAAACCCTTAGCGTTACCACC--GTGACGATTGCGTGCGATGAAAAGAGACCGTTGTCGGTTCATGCTTGACTTTAATCTAATCTAC
10 CGTGTGGCTGCGAAAACCCTTAGCGTTACCACC--GTGACGATTGCGTGCGATGAGAAGAGACCGTTGTCGGTTCATGCTTGACTTTAATCTAATCTAC
11 CGTGTGGCTGCGAAAACCCTTAGCGTTACCCCT--GTGACGATTGCGTGCGATGCAAACAGACCGTTGTCGGTTCATGCTTGACTTTAATCTCATCTAC
T. b. brucei
1 CGTGTGGCTGCGAAAACCCTTAGCGTTACCACCGTGACGATTGCGTGCGATGAAAAGAGACCGTTGTCGGTTCATGCTTGACTTTAATCTCATCTACGC
2 TGTGTGGCTGCGAAAACCCTTAGCGTTACCACTGTGACGATTGCGTGCGATGCAAAGAGACCGTTGTCGGTTCATGCATGACTTTAATCTCATCTACGC
3 TGTGTGGCTGCGAAAACCCTTAGCGTTACCACAGTGACGATTGCGTGCGATGCAAAGAGACCGTTGCCGGTTCATGCTTGACTTTAATCTCATCTACGC
4 CGTGTGGCTGCGAAAACCCTTAGCGTTACCGCTGTGACGATTGCATGCGATGCAAAGAGACCGTTGCCGGTTCATGCTTGACTTTAATCTCATCTACGC
5 CGTGTGGCTGCGAAAACCATTAGCGTTACCGCTGTGACGATTGCATGCGATGCAAAGAGACCGTTGTCGGTTCATGCATGACTTTAATCTCATCTACGC
6 CGTGTGGCTGCGAAAACGCTTACCGTCACCGTTGTGACGACTGCGTGCGATGAAAAGAGACCGTTGTCGGTTCATGCTTGACTTTAATCTGATCTACGC
7 TGTGTGGCTGCGAAAACCCTTAGCGTTACCACCGTGACGATTGCGTGCGATGAAAAGAGACCGTTGTCGGTTCATGCTTGACTTTAATCTCATCTACGC
8 TGTGTGGCTGCGAAAACCCTTAGCGTTACCACCGTGACGATTGCGTGCGATGAAAAGAGACCGTTGTCGGTTCATGCTTGACTTTAATCTCATCTACGC
9 TGTGTGGCTGCGAAAACCCTTAGCGTTACCACCGTGACGATTGCGTGCGATGAAAAGAGACCGTTGTCGGTTCATGCTTGACTTTAATCTCATCTACGC
10 CGTGTGGCTGCGAAAACCCTTAGCGTTACCACAGTGACGATTGCGTGCGATGCAAAGAGACCGTTGTCGGTTCATGCTTGACTTTAATCTCATCTACGC
11 CGTGTGGCTGCGAAAACCCTTAGCGTTACCACCGTGACGATTGCGTGCGATGAAAAGAGACCGTTGTCGGTTCATGCTTGACTTTAATCTCATCTACGC
12 CGTGTGGCTGCGAAAACCCTTAGCGTTACCACCGTGACGATTGCGTGCGATGAAAAGAGACCGTTGTCGGTTCATGCTTGACTTTAATCTCATCTACGC
13 TGTGTGGCTGCGAAAACCCTTAGCGTTACCACTGTGACGATTGCGTGCGATGAAAAGAGACCGTTGTCGGTTCATGCTTGACTTTAATCTCATCTACGC
14 TGTGTGGCTGCGAAAACCCTTAGCGTTACCACCGTGACGATTGCGTGCGATGAAAAGAGACCGTTGTCGGTTCATGCTTGACTTTAATCTCATCTACGC
15 TGTGTGGCTGCGAAAACCCTTAGCGTTACCACCGTGACGATTGCGTGCGATGAAAATAGACCGTTGTCGGTTCATGCTTGACTTTAATCTCATCTACGC
16 CGGGTGGCTGCGAAAACCCTTAGCGTTACCACCGTGACGATTGCGTGCGATGAAAAGAGACCGTTGTCGGTTCATGCTTGACTTTAATCTCATCTACGC
17 CGTGTGGCTGCGAAAACCCTTAGCGTTACCACCGTGACGATTGCGTGCGATGAAAAGAGACCGTTGTCGGTTCATGCTTGACTTTAATCTCATCTACGC
18 TGTGTGGCTGCGAAAACCCTTAGCGTTACCACTGTGACGATTGCGTGCGATGCAAAGAGACCGTTGTCGGTTCATGCATGACTTTAATCTCATCTACGC
T. equiperdum
1 CGTGTGGCTGCGAAAACCCTTAGCGTTACCACC--GTGACGATTGCGTGCGATGAAAAGAGACCGTTGTCGGTTCATGCTTGACTTTAATCTCATCTAC
2 TGTGTGGCTGCGAAAACCCTTAGCGTTACCCCCTGTGACGATTGCGTGCGATGCAAAGAGACCGTTGCCGGTTCATACTTGACTTTAATCTCATCTAC
3 CGTGTGGCTGCGAAAACCCTTAGCGTTACCACC--GTGACGATTGCGTGCGATGCAAACAGACCGTTGTCGGTTCATGCTTGACTTTAATCTCATCTAC
4 TGTGTGGCTGCGAAAACCCTTAGCGTTACCACT--GTGACGATTGCGTGCGATGCAAAGAGACCGTTGTCGGTTCATGCATGACTTTAATCTCATCTAC
5 CGTGTGGCTGCGAAAACCCTTAGCGTTACCCCT--GTGACGATTGCGTGCGATGCAAAGAGACCGTTGTCGGTTCATGCATGACTTTAATCTCATCTAC
6 CGTGTGGCTGCGAAAACCCTTAGCGTTACCACC--GTGACGATTGCGTGCGATGAAAAGAGACCGTTGCCGGCTCATGCTTGACTTTAATCTCATCTAC
7 CGTGTGGCTGCGAAAACCCTTAGCGTTACCCCT--GTGACGATTGCGTGCGATGCAAAGAGACCGTTGTCGGTTCATGCTTGACTTTAATCTCATCTAC
8 CGTGTGGCTGCGAAAACCCTTAGCGTTACCCCT--GTGACGATTGCGTGCGATGCAAAGAGACCGTTGTCGGTTCATGCTTGACTTTAATCTCATCTAC
9 CGTGTGGATGCGAAAACCATTAGCGTTACCGCT--GTGACGATTGCATGCGATGCAAAGAGACCGTTGTCGGTTCATGCATGACTTTAATCTCATCTAC
10 CGTGTGGATGCGAAAACCATTAGCGTTACCGCT--GTGACGATTGCATGCGATGCAAAGAGACCGTTGTCGGTTCATGCATGACTTTAATCTCATCTAC
11 CGTGTGGCTGCGAAAACCCTTAGCGTTACCACC--GTGACGATTGCGTGCGATGAAAAGAGACCGTTGTCGGTTCATGCTTGACTTTAATCTCATCTAC

BES promoter type		100 … 190
T. b. gambiense	1	GCTTTATCCCGTGGCTCCTTCGTCCATTGAGGTTTCTCTCTGGGGCAGGCTGAGCGTATTTTAGAGGGCTGGTGTGTTGTTTTGCATGCGAGGTGA-GGG
	2	GCTTTATCCCGTGGCTCCTTCGTCCATTGAGGTTTCTCTCTGGGGCAGGCTGAGCGTATTTTAGAGGCCTGGTGTGTTGCTTTGCAGGCGAGGTGAAGGG
	3	GCTTTATCCCGTGGCTCCTTCGTCCATTGAGGTTTCTCTCTGGGGCAGGCTGAGCGTATTTTAGAGGCCTGGTGTGTTGCTTTGCAGGCGAGGTGAAGGG
	4	GCTTTATCCCGTGGCTCCTTCGTCCATTGAGGTTTCTCTCTGGGGCAGGCTGAGCGTATTTTAGAGGCCTGGTGTGTTGCTTTGCAGGCGAGGTGAAGGG
	5	GCTTTATCCCGTGGCTCCTTCGTCCATTGAGGTTTCTCTCAGGGGCAGGCTGAGCGTATTTTAGAGGGCTGGTGTGTTGCTTTGCAGGCGAGGTGAAGGG
	6	GCTTTATCCCGTGGCTCCTTCGTCCATTGAGGTTTCTCTCTGGGGCAGGCTGAGCGTATTTTAGAGGGCTGGTGTGTTGCTTTGCAGGCGAGGTGAAGGG
	7	GCTTTATCCCGTGGCTCCTTCGTCCATTGAGGTTTCTCTCTGGGGCAGGCTGAGCGTATTTTAGAGGGCTGGTGTGTTGCTTTGCAGGCGAGGTGAAGGG
	8	GCTTTATCCCGTGGCTCCTTCGTCCATTGAGGTTTCTCTCTGGGGCAGGCTGAGCGTATTTTAGAGGCCTGGTGTGTTGCTTTGCAGGCGAGGTGAAGGG
	9	GCTTTATCCCGTGGCTCCTTCGTCCATTGAGGTTTCTCTCTGGGGCAGGCTGAGCGTATTTTAGAGGCCTGGTGTGTTGCTTTGCAGGCGAGGTGAAGGG
	10	GCTTTATCCCGTGGCTCCTTCGTCCATTGAGGTTTCTCTCTGGGGCAGGCTGAGCGTATTTTAGAGGCCTGGTGTGTTGCTTTGCAGGCGAGGTGAAGGG
	11	GCTTTATCCCGTGGCTCCTTCGTCCATTGAGGTTTCTCTCTGGGGCAGGCTGGGCGTATTTTAGAGGGCTGGTGTGTTGCTTTGCAGGCGAGGTGAAGGG
T. b. brucei	1	TTTATCCCGTGCATACTGCGTCCATTGAGGTTTCTCTCTGGGGCAGGCTGAGCGTATTTTAGAGGGCTGGTGTGTTGCTTTGCATGCGAAGTGAAGCGGC
	2	TTTATCCCGTGGTTCCTTTGTCCATTGAGGTTTCTCTCTGGGACGGGCTGAGCGTATTTTAGAGGGCTGGTGTGTTGTTTTGCATGCGAAGAGCAGGGG-
	3	TTTATCCCGTGGCTCCTTCGTCCATTGAGGTTTCTCTCTGGGGCAGGCTGAGCGTATTTTAGAGGGCTGGTGTGTTGCTTTGCATGCGAAGTGAAGCGGC
	4	TTTATCCCGTGGCTCCTCCGTCCATTGAGGTTTCTCTCTGGGACGGGCTGAGCGTATTTTAGAGGGCTGGTGTGTTGTTTTTCATGCGACGGAAGGGG-
	5	TTTATCCCGTGGCTCCTTCGTCCATTGAGGTTTCTCTCTGGGGCAGGCTTAGCGTATTTTAGAGGGCTGGTGTGTTGCTTTGCATGCGAGGTGAAGGGGC
	6	TTCATCCCGTGGCTCCTTCGTCCATTGAGGTTTCTCTCTGGGGCAGGCTGAGCGTATTTAAGGGGGCTGGTGTGTTATTTTTCATGCGACGGAAGGGG-
	7	TTTATCCCGTGGCTCCTTCGTCCATTGAGGTTTCTCTCTGGGGCAGGCTGAGCGTATTTTAGAGGGCTGGTGTGTTGTTTTTCATGCGACGGAAGGGGT
	8	TTTATCCCGTGGCTCCTTCGTCCATTGAGGTTTCTCTCTGGGGCAGGCTGAGCGTATTTTAGAGGGCTGGTGTGTTGTTTTTCATGCGACGGAAGGGGT
	9	TTTATCCCGTGGCTCCTTCGTCCATTGAGGTTTCTCTCTGGGGCAGGCTGAGCGTATTTTAGAGGGCTGGTGTGTTGTTTTTCATGCGACGGAAGGGG-
	10	TTTATCCCGTGGCTCCTTCGTCCATTGAGGTTTCTCTCTGGGGCAGGCTGAGCGTATTTTAGAGGGCTGGTGTGTTGCTTTGCATGCGAAGTGAAGCAGC
	11	TTTATCCCGTGGCTCCTTCGTCCATTGAGGTTTTTCTCTGGGGCAGGCTGAGCGTATTTTAGAGGGCTGGTGTGTTGCTTTGCATGCGAGGTGAAGGGGT
	12	TTTATCCCGTGGCTCCTTCGTCCATTGAGGTTTCTCTCTGGGGCAGGCTGAGCGTATTTTAGAGGGCTGGTGTGTTGATTTGCATGCGAAGTGAAGCGGC
	13	TTTATCCCGTGGCTCCTTCGTCCATTGAGGTTTCTCTCTGGGGCAGGCTGAGCGTATTTTAGAGGGCTGGTGTGTTGCTTTGCAGGCGAGGTGAAGGGGC
	14	TTTATCCCGTGGCTCCTTCGTCCATTGAGGTTTTTCTCTGGGGCAGGCTGAGCGTATTTTAGAGGGCTGGTGTGTTGCTTTGCATGCGAAGTGAAGCGGC
	15	TTCATCCCGTGGCTCCTTCGTCCATTGAGGTTTCTCTCTGGGGCAGGCTGAGCGTATTTTAGAGGGCTGGTGTGTTGCTTTGCATGCGAAGTGAAGGGGC
	16	TTTATCCCGTGGCTCCTTCGTCCATTGAGGTTTCTCTCTGGGGCAGGCTGAGCGTATTTTAGAGGGCTGGTGTGTTGCTTTGCATGCGAAGTGAAGCGGC
	17	TTTATCCCGTGGCTCCTTCGTCCATTGAGGTTTCTCTCTGGGGCAGGCTGAGCGTATTTTAGAGGGCTGGTGTGTTGCTTTGCAGGCGAGGTGAAGGGGC
	18	TTTATCCCGTGGTTCCTTTGTCCATTGAGGTTTCTCTCTGGGACGGGCTGAGCGTATTTTAGAGGGCTGGTGTGTTGTTTTGCATGCGAAGAGCAGGGG-
T. equiperdum	1	GCTTTATCCCGTGGCTCCTTCGTCCATTGAGGTTTCTCTCTGGGGCAGGCTGAGCGTATTTTAGAGGGCTGGTGTGTTGCTTTGCATGCGAGGTGAAGGG
	2	GCTTTATCCCGTGGCTCCTTTGTCCATTGAGGTTTCTCTCTGGGACGGGCTGAGCGTATTTTAGAGGGCTGGTGTGTTGTTTTGCATGCGAAGAGCAGGG
	3	GCTTTATCCCGTGGCTCCTTTGTCCATTGAGGTTTCTCTCTGGGACGGGCTGAGCGTATTTTAGAGGGCTGGTGTGTTGTTTTGCATGCGAGGTG-AGGG
	4	GCTTTATCCCGTGGTTCCTTTGTCCATTGAGGTTTCTCTCTGGGACGGGCTGAGCGTATTTTAGAGGGCTGGTGTGTTGTTTTGCATGCGAAGAGCAGGG
	5	GCTTCATCCCGTTGCTCCTTCGTCCATTGAGGTTTCTCTCTGGGACGGGCTGAGCGTATTTTAGAGGGCTGGTGTGTTGTTTTGCATGCGAAGAGCAGGG
	6	GCTTTATCCCGTGGCTCCTTCGTCCATTGAGGTTTCTCTCTGGGGCAGGCTGAGCGTATTTTAGAGGGCTGGTGTGTTGCTTTGCATGCGAAGTGAAGCG
	7	GCTTTATCCCGTGGCTCCTTCGTCCATTGAGGTTTCTCTCTGGGGCAGGCTGAGCGTATTTTAGAGGGCTGGTGTGTTGCTTTGCATGCGAGGTGAAGGG
	8	GCTTTATCCCGTGGCTCCTTCGTCCATTGAGGTTTCTCTCTGGGGCAGGCTGAGCGTATTTTAGAGGGCTGGTGTGTTGCTTTGCATGCGAGGTGAAGGG
	9	GCTTTATCCCGTGGCTCCTTCGTCCATTGAGGTTTCTCTCTGGGGCAGGCTGAGCGTATTTTAGAGGGCTGGTGTGTTGCTTTGCATGCGAAGTGAAGCG
	10	GCTTTATCCCGTGGCTCCTTCGTCCATTGAGGTTTCTCTCTGGGGCAGGCTTAGCGTATTTTAGAGGGCTGGTGTGTTGCTTTGCATGCGAGGTGAAGGG
	11	GCTTTATCCCGTGGCTCCTTCGTCCATTGAGGTTTCTCTCTGGGGCAGGCTGAGCGTATTTTAGAGGGCTGGTGTGTTGCTTTGCATGCGAAGTGAAGCG

BES promoter type		200 210 220 230 240 250 260 270 280 290
T. b. gambiense	1	GCTGCAACGGAAGAGTGAAGGGGTTTGAGGAAATAGTGGAATTAGCATGATTGGAGATTGTGAGGGTTAGGAAAAACGTGGCGAGGGAAACGGTAACTGG
	2	GCTGCAACGGAAGAGTGAAGGGGTTTGAGGAAATAGTGGAATTAGCATGATTGGAGATTGTGAGGGTTAGGAAAAGCGTGGCGAGGGAAACGGTAACTGG
	3	GCTGCAACGGAAGAATGAAGGGGTTTGAGGAAATAGTGGGATTAGCATGATTGGAGATTGTGAGGGTTAGGAAAAGCGTGGCGAGGGAAATGGTAACTGG
	4	GCTGCAACGGAAGAGTGAAGGGGTTTGAGGAAATAGTGGAATTAGCATGATTGGAGATTGTGAGGGTTAGGAAAAGCGTGGCGAGGGAAATGGTAACTGG
	5	GCTGCAACGGAAGAATGAAGGGGTTTGAGGAAATAGTGGAATTAGCATGATTGGAGATTGTGAGGGTTAGGAAAAGCGTGGCGAGGGAAACGGTAACTGG
	6	GCTGCAACGGAAGAGTGAAGGGGTTTGAGGAAATAGTGGAATTAGCATGATTGGAGATTGTGAGGGTTAGGAAAAGCGTGGCGAGGGAAACGGTAACTGG
	7	GCTGCAACGGAAGAGTGAAGGGGTTTGAGGAAATAGTGGAATTAGCATGATTGGAGATTGTGAGGGTTAGGAAAAGCGTGGCGAGGGAAACGGTAACTGG
	8	GCTGCAACGGAAGAGTGAAGGGGTTTGAGGAAATAGTGGAATTAGCATGATTGGAGATTGTGAGGGTTAGGAAAAGCGTGGCGAGGGAAACGGTAACTGG
	9	GCTGCAACGGAAGAGTGAAGGGGTTTGAGGAAATAGTGGAATTAGCATGATTGGAGATTGTGAGGGTTAGGAAAAGCGTGGCGAGGGAAACGGTAACTGG
	10	GCTGCAACGGAAGAGTGAAGGGGTTTGAGGAAATAGTGGAATTAGCATGATTGGAGATTGTGAGGGTTAGGAAAAGCGTGGCGAGGGAAACGGTAACTGG
	11	GCTGCAACGGAAGAATGAAGGGGTTTGAGGAAATAGTGGGATTAGCATGATTGGAGATTGTGAGGGTTAGGAAAAGCGTGGCGAGGGAAATGGTAACTGG
T. b. brucei	1	TGCAACGGAAGAGTGAAGGGGTTTGAGGAAATAGTGGAATTAGCATGATTGGAGATTGTGAGGGTTAGGAAAAACGTGGCGAGGGAAATGGTAACTGGAC
	2	TGCAACGGAAGAGTGAAAGGATTAGAGGAAATGGTGGAATTAGCATGATCGGAGATTGTGAGGGTTAGGAAAAACGTGAAGAGGGAAATGGTAACTGGAC
	3	TGCAACGGAAGAGTGAAGGGGGTTGAGGAAATAGTGGAATTAGCAGGACCGGAGATTGTGAGGGTTAGGAAAAACGTGGCGAGGGAAACGGTAACTGGAC
	4	TGCAACGGAAGAGTGAAGGGGTTTGAGGAAATAGTGGAATTAGCATGATTGGAGATTGTGATGGTTAGGAAAAACGTGGCGAGGGAAACGGTAACTGGAC
	5	TGCAACGGAAGAGTGAAGGGGTTTGAGGAAATAGTGGGATTAGCATGATTGGAGATTGTGAGGGTTAGGAAAAACGTGGCGAGGGAAATTGTAACTGGAC
	6	TGCAACGGAAGAGTGAAAGGGTTTGAGGAAATAGTGGAATTAGCATGATTGGAGATTGTGA-GGATAGGAAAAACATGGCGAGGGAAACGGTAATTGGAC
	7	G-CGGCGGAAGAGTGAAGGGGGTTGAGGAAATAGTGGAATTAGCAGGACCGGAGATTGTGAGGGTTAGGAAAAGCGTGGCGAGGGAAACGGTAACTGGAC
	8	G-CGGCGGAAGAGTGAAGGGGGTTGAGGAAATAGTGGAATTAGCATGATTGGAGATTGTGAGGGTTAGGAAAAACGTGGCGAGGGAAATGGTAACTGGAC
	9	TGCGGCGGAAGAGTGAAGGGGGTTGAGGAAATAGTGGAATTAGCATGATTGGAGATTGTGAGGGTTAGGAAAAACGTGGCGAGGGAAATGGTAACTGGAC
	10	TGCAACGGAAGAGTGAAGGGGTTTGAGGAAATAGTGGAATTAGCATGATTGGAGATTTTGAGGGTTAGGAAAAACGTGGCGAGGGAAACGGTAACTGGAC
	11	T-CAACGGAAGAGTGAAGGGGTTTGAGGAAATAGTGGAATTAGCATGATTGGAGATTGTGGGGGTTAGGAAAAACGTGGCGAGGGAAACGGTAACTGGAC
	12	TGCAACGGAAGATTGAAGGGGTTTGAGGAAATAGTGGAATTAGCATGATTGGAGATTGTGAGGGTTAGGAAGAGCGTGGCGAGGGAAACGGTAACTGGAC
	13	TGCAACGGAAGAGTGAAGGGGTTTGAGGAAATAGTGGAATTAGCATGATTGGAGATTGTGAGGGTTAGGAAAAACGTGACGAGGGAAATGGTAACTGGAC
	14	TGCAACGGAAGAGTGAAGGGGTTTGAGGAAATAGTGGAATTAGCATGATTGGAGATTGTGGGGGTTAGGAAAAACGTGGCGAGGGAAACGGTAACTGGAC
	15	TGCAACGGAAGAGTGAAGGGGTTTGAGGAAATAGTGGAATTAGCATGATTGGAGATTGTGAGGGTTAGGAAAAACGTGGCGAGGGAAACGGTAACTGGAC
	16	TGCAACGGAAGAGTGAAGGGGTTTGAGGAAATAGTGGAATTAGCATGATTGGAGATTGTGAGGGTTAGGAAAAACGTGGCGAGGGAAATGGTAACTGGAC
	17	TGCAACGGAAGAGTGAAGGGGTTTGAGGAAATAGTGGGATTAGCATGATTGGAGATTGTGAGGGTTAGGAAAAACGTGGCGAGGGAAATGGTAACTGGAC
	18	TGCAACGGAAGAGTGAAAGGATTAGAGGAAATAGTGGAATTAGCATGATTGGAGATTGTGAGGGTTAGGAAAAACGTGACGAGGGAAATGGTAACTGGAC
T. equiperdum	1	G-TTCAACGGAAGAGTGAAGGGGTTTGAGGAAATAGTGGAATTAGCATGATTGGAGATTGTGAGGGTTAGGAAAAACGTGGCGAGGGAAACGGTAACTGG
	2	G-TGCAACGGAAGAGTGAAGGGGTTTGAGGAAATGGTGGAATTAGCATGATTGGAGATTGTGAGGGTTAGGAAAAACGTGACGAAGGAAATGGTAACTGG
	3	GCTGCAACGGAAGAGTGAAGGGGTTTGAGGAAATAGTGGAATTAGCATGATTGGAGATTGTGAGGGTTAGGAAAAACGTGGCGAGGGAAATGGTAACTGG
	4	G-TGCAACGGAAGAGTGAAAGGTTTAGAGGAAATAGTGGAATTAGCATGATTGGAGATTGTGAGGGTTAGGAAAAACGTGACGAGGGAAATGGTAACTGG
	5	G-TGCAACGGAAGAGTGAAGGGGTTTGAGGAAATAGTGGAATTAGCATGATTGGAGATTGTGAGGGTTAGGAAAAACGTGACGAGGGAAATGGTAACTGG
	6	GCTGCAACGGAAGAGTGAAGGGGTTTGAGGAAATAGTGGAATTAGCATGATTGGAGATTGTGAGGGTTAGGAAAAACGTGGCGAGGGAAATGGTAACTGG
	7	GCTGCAACGGAAGAGTGAAGGGGTTTGAGGAAATAGTGGAATTAGCATGATTGGAGATTGTGAGGGTTAGGAAAAACGTGGCGAGGGAAATGGTAACTGG
	8	GCTGCAACGGAAGAGTGAAGGGGTTTGAGGAAATAGTGGAATTAGCATGATTGGAGATTGTGAGGGTTAGGAAAAACGTGGCGAGGGAAATGGTAACTGG
	9	GCTGCAACGGAAGAGTGAAGGGGTTTGAGGAAATAGTGGGATTAGCATGATTGGAGATTGTGAGGGTTAGGAAAAACGTGGCGAGGGAAATTGTAACTGG
	10	GCTGCAACGGAAGAGTGAAGGGGTTTGAGGAAATAGTGGGATTAGCATGATTGGAGATTGTGAGGGTTAGGAAAAACGTGGCGAGGGAAATTGTAACTGG
	11	GCTGCAACGGAAGAGTGAAGGGGTTTGAGGAAATAGTGGAATTAGCATGATTGGAGATTGTGAGGGTTAGGAAAAACGTGGCGAGGGAAATGGTAACTGG

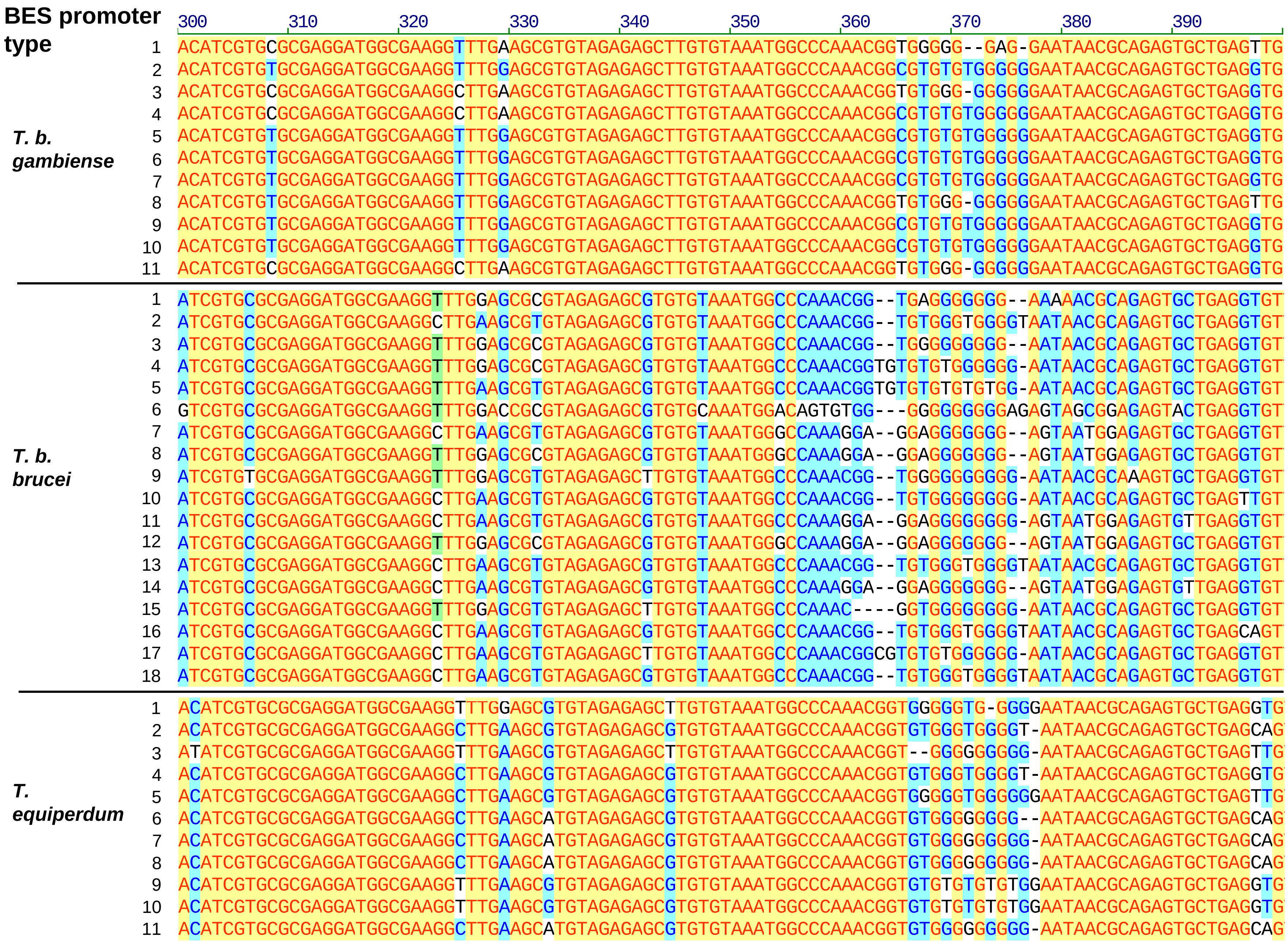

BES promoter type
300
310
320
330
340
350
360
370
380
390
T. b. gambiense
T. b. brucei
T. equiperdum

BES promoter type		400 410 420 430 440 450 460 470 480 490
T. b. gambiense	1	TTTGATTGGCAAGTGCCCGCGAGGGTAGCTGGGGAGGAGGAAGCAAGACAGTAAAATCGTCGTGAGACGGTGGCTGTGCTCGGGAATCTGTACAAAATA
	2	TTT-ATTGGCAAGTGCCCGCGAGGGGTGCTGGGGAGGAGGAAGCAAGACAGTAAAATCGTCGTGAGACGGTGGCTGTGCTCGGGAATCTCTACAAAATA
	3	TTT-ATTGGCAAGTGCCCGCGAGGGGTGCTGGGGAGGAGGAAGCAAGACAGTAAAATCGTCGTGAGACGGTGGCTGTGCTCGGGAATCTCTACAAAATA
	4	TTT-ATTGGCAAGTGCCCGCGAGGGGTGCTGGGGAGGAGGAAGCAAGACAGTAAAATCGTCGTGAGACGGTGGCTGTGCTCGGGAATCTCTACAAAATA
	5	TTT-ATTGGCAAGTGCCCGCGAGGGGTGCTGGGGAGGAGGAAGCAAGACAGTAAAATCGTCGTGAGACGGTGGCTGTGCTCGGGAATCTCTACAAAATA
	6	TTT-ATTGGCAAGTGCCCGCGAGGGGTGCTGGGGAGGAGGAAGCAAGACAGTAAAATCGTCGTGAGACGGTGGCTGTGCTCGGGAATCTCTACAAAATA
	7	TTT-ATTGGCAAGTGCCCGCGAGGGGTGCTGGGGAGGAGGAAGCAAGACAGTAAAATCGTCGTGAGACGGTGGCTGTGCTCGGGAATCTCTACAAAATA
	8	TTTGATTGGCAAGTGCCCGCGAGGGGTGCTGGGGAGGAGGAAGCAAGACAGTAAAATCGTCGTGAGACGGTGGCTGTGCTCGGGAATCTGTACAAAATA
	9	TTT-ATTGGCAAGTGCCCGCGAGGGGTGCTGGGGAGGAGGAAGCAAGACAGTAAAATCGTCGTGAGACGGTGGCTGTGCTCGGGAATCTCTACAAAATA
	10	TTT-ATTGGCAAGTGCCCGCGAGGGGTGCTGGGGAGGAGGAAGCAAGACAGTAAAATCGTCGTGAGACGGTGGCTGTGCTCGGGAATCTCTACAAAATA
	11	TTT-ATTGGCAAGTGCCCGCGAGGGGTGCTGGGGAGGAGGAAGCAAGACAGTAAAATCGTCGTGAGACGGTGGCTGTGCTCGGGAATCTCTACAAAATA
T. b. brucei	1	TT-ATTGGCAAGTGCCCGCGAGGGATGCTGAGCAGGAGGAAGCGAGACAGTAAAAAT-TGGTGAGACGGTGGCTGCGCTCGGCAATCTGTACAAAAA-TA
	2	TT----GACAAGTGCCCGCGAGGGGTGCTGGGGAGGAGGAAGCAAGACAGTAAAATCGTCGTGAGACGGTGGCTGTGCTCGGGAATCCGTACAAATA-TA
	3	TTGATTGGCAAGTGCCCGCGAGGGATGCTGAGCAGGAGGAAGCGAGACAGTAAAAAT-TGGTGAGACGTTGGCTGCGCTCGGCAATCTGTACAAAAA-TA
	4	TTGATTAGCATGTGCCCGCGAGGGGTGCTGAGGAGGAGGAAGCAAGACAGTAAAATCGTCGTGAGACTGTGGCTGTGCTCGGGAATCCGTACAAAAA-TA
	5	TTGATTGGCAGGTGCCCGCGAGGGGTGCTGAGGAGGAGGAAGCAAGACAGTAAAATCGTCGTGAGACGGTGGCTGCACTCGGAAATCCGTACAAAAA-TA
	6	TTGATTGGCAAGTGCCAGCGAGGGATGCTGAGCAGGAGGAAGGGAGACAGTAAAATCGTCGTGAGACGGTGGCTGCACTCGGGAATCCGTACAAAAC-TA
	7	TTGATTGGCAAGTGCCCGCGAGGGGTGCTGAGCAGGAGGAAGCGAGACAGTAAAAAT-TGGTGAGACGGTGGCTGCACTCGGGAATCCGTACAAAAAATA
	8	TTGATTGGCAAGTGCCCGCGAGGGGTGCTGAGCAGGAGGAAGCGAGACAGTAAAAAT-TGGTGAGACGGTGGCTGCACTCGGGAATCCGTACAAAAAATA
	9	TT-ATTGGCAAGTGCCCGCGAGGGGTTCTGGGGAGGAGGAAGCAAGACAGTAAAATCGTCGTGAGACGGTGGCTGTGCTCGGGAATCTGTACAAAAA-TA
	10	TTGATTGGCAAGTGCCCGCGAGGGGTGCTGGGGAGGAGGAAGCAAGACAGTAAAATCGTCGTGAGACGGTGGCTGTGCTCGGGAATCTGTACAAAAA-TA
	11	TTGATTGGCAAGTGCCCGCGAGGGGTGCTGAGCAGGAGGAAGCGAGACAGTAAAAAT-TGGTGAGACGGTGGCTGCACTCGGGAATCCGTACAAAAAATA
	12	TTGATTGGCAAGTGCCAGCGAGGGGTGCTGAGCAGGAGGAAGCGAGACAGTAAAAAT-TGGTGAGACGGTGGCTGCACTCGGGAATCCGTACAAAAAATA
	13	TT----GACAAGTGCCCGCGAGGGGTGCTGGGGAGGAGGAAGCAAGACAGTAAAATCGTCGTGAGACGGTGGCTGTGCTCGGGAATCCGTACAAATA-TA
	14	TTGATTGGCAAGTGCCCGCGAGGGGTGCTGAGCAGGAGGAAGCGAGACAGTAAAAAT-TGGTGAGACGGTGGCTGCACTCGGGAATCCGTACAAAAAATA
	15	TTGATTGGCAAGTGCCCGCGAGGGATGCTGAGCAGGAGGAAGCGAGATAGTAAAAAT-TGGTGAGACGGTGGCTGCACTCGGGAATCCGTACAAAAAATA
	16	TT-ATTGGCAAGTGCCCGCGAGGGGTGCTGGGGAGGAGGAAGCAAGAAAGTAAAATCGTCGTGAGAGGGTGGCTGTGCTCGGGAATCCGTACAAAAA-TA
	17	TT-ATTGGCAAGTGCCCGCGAGGGGTGCTGGGGAGGAGGAAGCAAGACAGTAAAATCGTCGTGAGACGGTGGCTGTGATCGGGAATCTCTACAAAAA-TA
	18	TT----GACAAGTGCCCGCGAGGGGTGCTGGGGAGGAGGAAGCAAGACAGTAAAATCGTCGTGAGACGGTGGCTGTGCTCGGGAATCCGTACAAATA-TA
T. equiperdum	1	TTTGATTGGCAAGTGCCCGCGAGGGGTGCTGAGCAGGAGGAAGCGAGACAGTAAAATCGTCGTGAGACGGTGGCTGCACTCGGGAATCCGTACAAAAAAT
	2	TTT-ATTGGAAAGTGCCCGCGAGGGGTGCTGGGGAGGAGGAAGCAAGAAAGTAAAATCGTCGTGAGAGGGTGGCTGCACTCGGGAATCCGTACAAAAA-
	3	TTTGATTGGCAAGTGCCCGCGAGGGGTGCTGGGGAGGAGGAAGCAAGACAGTAAAATCGTCGTGAGACGGTGGCTGTGCTCGGGAATCTGTACAAAAA-
	4	TTTGA----CAAGTGCCCGCGAGGGGTGCTGGGGAGGAGGAAGCAAGACAGTAAAATCGTCGTGAGACGGTGGCTGTGCTCGGGAATCCGTACAAATA-
	5	TTTGATTGGCAAGTGCCCGCGAGGGGTGCTGAGGAGGAGGAAGCAAGACAGTAAAATCGTCGTGAGACGTTGGCTGTGCTCGGGAATCCGTACAAATA-
	6	TTT-ATTGGAAAGTGCCCGCGAGGGGTGCTTGGGAGGAGGAAGCAAGAAAGTAAAATCGTCGTGAGAGGGTGGCTGTGCTCGGGAATCCGTACAAAAA-
	7	TTT-ATTGGAAAGTGCCCGCGAGGGGTGCTGGGGAGGAGGAAGCAAGAAAGTAAAATCGTCGTGAGAGGGTGGCTGTGCTAGGGAATCCGTACAAAAA-
	8	TTT-ATTGGAAAGTGCCCGCGAGGGGTGCTGGGGAGGAGGAAGCAAGAAAGTAAAATCGTCGTGAGAGGGTGGCTGTGCTAGGGAATCCGTACAAAAA-
	9	TTTGATTGGCAGGTGCCCGCGAGGGGTGCTGAGGAGGAGGAAGCAAGACAGTAAAATCGTCGTGAGACGGTGGCTGCACTCGGAAATCCGTACAAAAA-
	10	TTTGATTGGCAGGTGCCCGCGAGGGGTGCTGAGGAGGAGGAAGCAAGACAGTAAAATCGTCGTGAGACGGTGGCTGCACTCGGAAATCCGTACAAAAA-
	11	TTT-ATTGGAAAGTGCCCGCGAGGGGTGCTTGGGAGGAGGAAGCAAGAAAGTAAAATCGTCGTGAGAGGGTGGCTGTGCTCGGGAATCCGTACAAAAA-

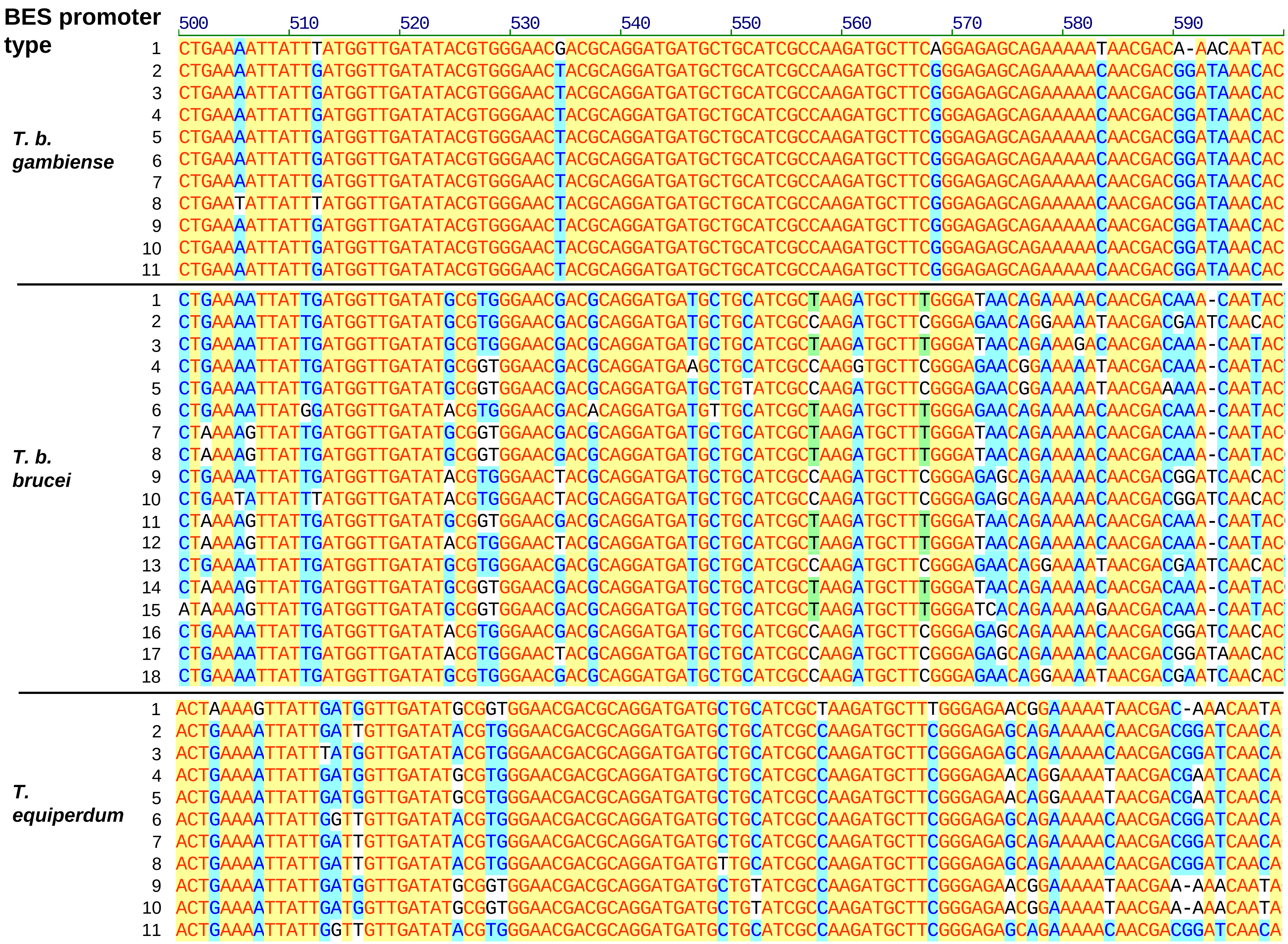
BES promoter type
500 510 520 530 540 550 560 570 580 590
T. b. gambiense
1 2 3 4 5 6 7 8 9 10 11
T. b. brucei
1 2 3 4 5 6 7 8 9 10 11 12 13 14 15 16 17 18
T. equiperdum
1 2 3 4 5 6 7 8 9 10 11

BES promoter type		600 610 620 630 642
T. b. gambiense	1	CGGTGAGTTGATACTATAAAAATCACGCGGGCGTTGCTGCTTC
	2	TGGTGAGTTGATACTATAAAAATCACGCGGGCGTTGCTGCTTC
	3	TGGTGAGTTGATACTATAAAAATCACGCGGGCGTTGCTGCTTC
	4	TGGTGAGTTGATACTATAAAAATCACGCGGGCGTTGCTGCTTC
	5	TGGTGAGTTGATACTATAAAAATCACGCGGGCGTTGCTGCTTC
	6	TGGTGAGTTGATACTATAAAAATCACGCGGGCGTTGCTGCTTC
	7	TGGTGAGTTGATACTATAAAAATCACGCGGGCGTTGCTGCTTC
	8	TGGTGAGTTGATACTATGAA--TCACGCGGGCGTTGCTGCTTC
	9	TGGTGAGTTGATACTATAAAAATCACGCGGGCGTTGCTGCTTC
	10	TGGTGAGTTGATACTATAAAAATCACGCGGGCGTTGCTGCTTC
	11	TGGTGAGTTGATACTATAAAAATCACGCGGGCGTTGCTGCTTC
T. b. brucei	1	TGGTGAGTTGATACTATAAAAAT-CGCGTGGGCGTTTCTGCTTC
	2	TGGTGAGTTGATACTATAAAAAT-CGCGTGGGCTTTGCTGCTTC
	3	TGGTGAGTTGATACTATAAAAAT-CGCGTGGGCGTTTCTGCTTC
	4	TGGTGAGTTGATACTA--AAAAT-CACGTGGGCGTTGCTGCTTC
	5	TGGTGAGTTGATACTATAAAAAT-CACGCTGGCGCTGCTGCTTC
	6	TGGTGAGTTGATACTATAAAGGC-CACATGGACGTTGCTGTTTC
	7	CGGTGAGTTGATACTATAAAAAT-CACGCGGGCGTTGCTGCTTC
	8	CGGTGAGTTGATACTATAAAAAT-CACGCGGGCGTTGCTGCTTC
	9	TGGTGAGTTGATACTATAAAAAT-CACGCGGGCGTTGCTGCTTC
	10	TGGTGAGTTGATACTAAGAAT---CACGCGGGCGTTGCTGCTTC
	11	CGGTGAGTTGATACTATAAAAAT-CACGCGGGCGTTGCTGCTTC
	12	CGGTGAGTTGATACTATAAAAAT-CATGCGGGCGTTGCTGCTTC
	13	TGGTGAGTTGATACTATAAAAAT-CGCGTGGGCTTTGCTGCTTC
	14	CGGTGAGTTGATACTATAAAAAT-CACGCGGGCGTTGCTGCTTC
	15	TGGTGAGTTGATACTATAAAAAT-CGCGTGGGCGTTTCTGCTTC
	16	TGGTGAGTTATTACTATAAAGGC-CACGCGGGCGTTGCTGCTTC
	17	TGGTGAGTTGATACTATAAAAAT-CACGCGGGCGTTGCTGCTTC
	18	TGGTGAGTTGATACTATAAAAATTCGCGTGGGCTTTGCTGCTTC
T. equiperdum	1	CCGGTGAGTTGATACTATAAAAATCACGCGGGCGTTGCTGCTTC
	2	CTGGTGAGTTGATACTATAAAGGCCACGCGGGCGTTGCTGCTTC
	3	CTGGTGAGTTGATACTATAAAGGCCACGCGGGCGTTGCTGCTTC
	4	CTGGTGAGTTGATACTATAAAAATCGCGTGGGCTTTGCTGCTTC
	5	CTGGTGAGTTGATACTATAAAAATCGCGTGGGCTTTGCTGCTTC
	6	CTGGTGAGTTGATACTATAAAGGCCACGCGGGCGTTGCTGCTTC
	7	CTGGTGAGTTGATACTATAAAGGCCACGCGGGCGTTGCTGCTTC
	8	CTGGTGAGTTGATACTATAAAGGCCACGCGGGCGTTGCTGCTTC
	9	CTGGTGAGTTGATACTATAAAAATCACGCTGGCGCTGCTGCTTC
	10	CTGGTGAGTTGATACTATAAAAATCACGCTGGCGCTGCTGCTTC
	11	CTGGTGAGTTGATACTATAAAGGCCACGCGGGCGTTGCTGCTTC
