# Supplementary material for: Isolation and analysis of the genetic diversity of repertoires of VSG expression site containing telomeres from Trypanosoma brucei gambiense, T. b. brucei and T. equiperdum
Source: BMC Genomics. 2008 Aug 12;9:385. doi: 10.1186/1471-2164-9-385 (PMC2533676; doi:10.1186/1471-2164-9-385)
Supplement: Additional file 3 — Sup. Figure 3. Sequence alignments of ESAG6 sequences analysed in this manuscript. [file 1471-2164-9-385-S3.pdf]

	ESAG6p type	
T. b. gambiense	1	MMRFWFVLLALLGKETYA--YENERNALNATAANKVCGLSTYLKGIAHRVNSESAVVTEKLSDLKMRSIQLQLSVMRNRVPSGEKDCKDIRTLLKTVLR
	2	MMRFWFVLLALLGKETYA--YENERNALNATAANKVCGLSTYLKGIAHRVNSESAVVTEKLSDLKMRSIQLQLSVMRNRVPSGEKDCKDIRTLLKTVLR
	3	MMRFWFVLLALLGKETYA--YENERNALNATAANKVCGLSTYLKGIAHRVNSESAVVTEKLSDLKMRSIQLQLSVMRNRVPSGEKDCKDIRTLLKTVLR
	4	-MRFLFVLLAVLGKETYANYYENERNALNATAANKVCALSTYLKGIAHRVNSESAVVTEKLSDLKMRSIQLQLSVMRNRVPSGEQDCKDIRTLLKTVLR
	5	-MRFLFVLLAVLGKETYANYYENERNALNATAANKVCALSTYLKGIAHRVNSESAVVTEKLSDLKMRSIQLQLSVMRNRVPSGEKDCKDIRTLLKTVLR
	6	MMRFWFVLLALLGKETYA--YENERNALNATAANKVCGLSTYLKGIAHRVNSESAVVTEKLSDLKLRSIQLQLSVMRNRVPSGEKDCKDIRTLLKTVLR
	7	MMRFWFVLLALLGKETYA--YENERNALNATAANKVCGLSTYLKGIAHRVNSESAVVTEKLSDLKMRSIQLQLSVMRNRVPSGEKDCKDIRTLLKTVLR
T. b. brucei	1	-MRFWFVLLALLGKEIYAYE--NERNALNATAANKVCGLSTYLKGIAHRVNSESAVVTEKLSDLKMRSIQLQLSVMRNRVPSGEKDCKDIRTLLKTVLR
	2	MMKFWFVLLALLGKETHAYYE-NKRNALNATAANKVCGLSTYLKGIAHRVNSESAVVTEKLSDLKMRSIQLQLSVMRNRVPSGEQDCKDIRTLLKTVLR
	3	-MRFWFVLLALLGKETYAYYE-NERNALNATAANKVCGLSTYLKGIAHRVNSESAVVTEKLSDLKMRSIQLQLSVMRNRVPSGEKDCKDIRTLLKTVLR
	5	-MRFLFVLLALLGKETHANYYENERNALNATAANKVCGLSTYLKGIAHRVNSESAVVTEKLSDLKMRSIQLQLSVMRNRVPSGEKDCKDIRTLLKTVLR
	6	MMRFLFVLLALLRKETHANYYENERNALNATAANKVCGLSTYLKGIAHRVNSESAVVTEKLSDLKMRSIQLQLSVMRNRVPSGEKDCKDIRTLLKTVLR
	7	-MRFWLVLLALLGKETYANYYENKRNALNATAANKVCRLSTYLKGIAHRVSSESAVVTEKLSDLKMKSIQLQLSILRNRVPSGEKDCKDIRTLLKTVLR
	8	-MRFLFVLLALLGKKTHAYYE-NERNALNATAANKVCGLSTYLKGIAHRVNSESAVVTEKLSDLKMRSIQLQLSVMRNRVPSGEQDCKDIRTLLKTVLR
	9	-MRFLFVLLALLGKKTHAYYE-NERNALNATAANKVCGLSTYLKGIAHRVNSESAVVTEKLSDLKMRSIQLQLSVMRNRVPSGEQDCKDIRTLLKTVLR
	10	-MRFLFVLLALLGKETYAYYE-NERNALNATAANKVCGLSTYLKGIAHRVNSESAVVTEKLSDLKMRSIQLQLSVMRNRVPSGEKDCKDIRTLLKTVLR
	11	MMKFWFVLLALLGKETHAYYE-NKRNALNATAANKVCGLSTYLKGIAHRVNSESAVVTEKLSDLKMRSIQLQLSVMRNRVPSGEQDCKDIRTLLKTVLR
	12	MMKFWFVLLALLGKETHAYYE-NKRNALNATAANKVCGLSTYLKGIAHRVNSESAVVTEKLSDLKMRSIQLQLSVMRNRVPSGEQDCKDIRTLLKTVLR
	13	-MRFLFVLLAVLGKETYA-YYENERNALNATAANKVCALSTYLKGIAHRVNSESAVVTEKLSDLKMRSIQLQLSVMRNRVPSGEQDCKDIRTLLKTVLR
	14	-MRFLFVLLALLGKEIYAYE--NERNALNATAANKVCGLSTYLKGIAHRVNSESAVVTEKLSDLKMRSIQLQLSIMRNRVPSGEKDCKDIRTLLKTVLR
	15	-MRFLFVLLALLGKKTHAYYE-NERNALNATAANKVCGLSTYLKGIAHRVNSESAVVTEKLSDLKMRSIQLQLSIMRNRVPSGEKDCKDIRTLLKTVLR
	16	-MRFLFVLLALLGKKTHAYYK-NERNALNATAANKVCALSTYLKGIAHRVNSESAVVTEKLSDLKMRSIQLQLSVMRNRVPSGEQDCKDISTLLKTVLR
	17	MMKFWFVLLALLGKETHAYYE-NKRNALNATAANKVCGLSTYLKGIAHRVNSESAVVTEKLSDLKMRSIQLQLSVMRNRVPSGEQDCKDIRTLLKTVLR
	18	MMKFWFVLLALLGKETHAYYE-NKRNALNATAANKVCGLSTYLKGIAHRVNSESAVVTEKLSDLKMRSIQLQLSVMRNRVPSGEQDCKDIRTLLKTVLR
	19	-MRFWFVLLALLGKEIYAYE--NERNALNATAANKVCGLSTYLKGIAHRVNSESAVVTEKLSDLKMRSIQLQLSVMRNRVPSGEQDCKDIRTLLKTVLR
	20	-MRFLFLLLVLLGKKTHANYYENKRNALNATAANKVCGLSTYLKGIAHRVNGESAVVTEKLSDLKIRSIQLQLSVMRNRVPSGEQDCKDIRTLLKTVLR
	21	-MRFWFVLLAVLGKETYA-YYENERNALNATAANKVCALSTYLKGIAHRVNSESAVVTEKLSDLKMRSIQLQLSVMRNRVPSGEKDCKDIRTLLKTVLR
	22	MMRFWFVLLALLGKETYAYE--NERNALNATAANKVCGLSTYLKGIAHRVNSESAVVTEKLSDLKMRSIQLQLSVMRNRVPSGEKDCKDIRTLLKTVLR
T. equiperdum	1	RMRFLFVLLALLGKETHANYYENERNALNATAANKVCALSTYLKGIAHRVNSESAVVTEKLSDLKMRSIQLQLSIMRNRVPSGEKDCKDIRTLLKTVLR
	2	RMRFWFVLLALLGKETHAYYYENKRNALNATAANKVCALSTYLKGIAHRINSESAVFTEKLSDLKMRSIQLQLSTMRNRVPSGEKDCKDIRTLLKTVLR
	3	RMRFLFVLLALLGKETHANYYENERNALNATAANKVCALSTYLKGIAHRVNSESAVVTEKLSDLKMRSIQLQLSIMRNRVPSGEKDCKDIRTLLKTVLR
	4	MMRFWFVLLALLGKETYA-YYENERNALNATAANKVCGLSTYLKGIAHRVNSESAVVTEKLSDLKMRSIQLQLSVMRNRVPSGEKDCKDIRTLLKTVLR
	5	RMRFLFVLLALLGKETHANYYENERNALNATAANKVCGLSTYLKGIAHRVNSESAVVTEKLSDLKMRSIQLQLSVMRNRVPSGEKDCKDIRTLLKTVLR
	6	MMRFWFVLLALLGKETYA-YYENERNALNATAANKVCGLSTYLKGIAHRVNSESAVVTEKLSDLKMRSIQLQLSVMRNRVPSGEKDCKDIRTLLKTVLR
	7	RMRFWFVLLALLGKETHANYYENERNALNATAANKVCALSTYLKGIAHRVNSESAVVTEKLSDLKMRSIQLQLSTMRNRVPSGEKDCKDIRTLLKTVLR

ESAG6p type

		100 110 120 130 140 150 160 170 180 190
T. b. gambiense	1	NEFTFQQELEEMRNASALAAAAAGLAAGRLEEWIFVFAQAAGGSSQFCISVGKHIAAEHGNLQECFDGKIGPETLYKIEDSRVKESAQKSLQLHEVLSSI
	2	NEFTFQQELEEMRNASALAAAAAGLAAGRLEEWIFVFAQAAGGSSQFCISVGKHIAAEHGNLQECFDGKIGPETLYKIEDSRVKESAQKSLQLHEVLSSI
	3	NEFTFQQELEEMRNASALAAAAAGLAAGRLEEWIFVFAQAAGGSSQFCISVGKHIAAEHGNLQECFDGKIGPETLYKIEDSRVKESAQKSLQLHEVLSSI
	4	NEFTFQQELEEMRNASALAAAAAGLAAGRLEEWIFVFAQAGGRSSQFCISTGKTGPAEYNNLQECFDGTIGPETLYKIEDSRVKESAKTRLLLHEVLSSI
	5	NEFTFQQELEEMRNASALAAAAAGLAAGRLEEWIFVFAQAAGGSSQFCISVGKHIAAEHGNLQECFDGKIGPETLYKIEDSRVKESAQKSLQLHEVLSSI
	6	NEFTFQQELEEMRNASALAAAAAGLAAGRLEEWIFVFAQAAGGSSQFCISVGKHIAAEHGNLQECFDGKIGPETLYKIEDSRVKESAQKSLQLHEVLSSI
	7	NEFTFQQELEEMRNASALAAAAAGLAAGRLEEWIFVFAQAAGGSSQFCISVGKHIAAEHGNLQECFDGKIGPETLYKIEDSRVKESAQKSLQLHEVLSSI
T. b. brucei	1	NEFTFQQELEEMRNASALAAAAAGLAAGRLEEWIFVFAQAADRSSQFCISVGKTIPPEQNNLQECFDGTIGPETLYKIEDSRVKESAKKSLQLHEALSSI
	2	NEFTFQQELEEMRNASALAAAAAGIAAGRLEEWIFVFAQAAGGSSQFCISVGTNIPAEYNNLQECFDGIIGPETLYKIEDSRVKESAQKSLQLHEVLSSI
	3	NEFTFQQELEEMRNTSALAAAAAGIAAGRLEEWIFVFAQAAGRSSQFCISVGKTGPAEYNNLQECFDGTIGPETLYKIEDSRVKESAKTSLQLHEVLSSI
	5	NEFTFQQELEEMRNASALAAAAAGLAAGRLEEWIFVFAQAAGGSSQFCISVGRTGPAEYNNLQECFDGKIGPETLYKIEDSRVKESAQKSLQLHEVLSSI
	6	NEFTFQQELEEMRNASALAAAAAGLAAGRLEEWIFVFAQAAGGSSQFCISVGKNIPAEHKNLQECFDGKIGPETLYKIEDSRVKESAQKSLQLHEVLSSI
	7	NEFTFQQELEEMRNASALAAAAAGIAAGRLEEWIFVFAQAAGMTSKFCISVGGSRPAVHDKLQECFDGTIGPETLYKIEDSRVKESAQKSLQLHEALSSI
	8	NEFTFQQELEEMRNASALAAAAAGLAAGRLEEWIFVFAQAADGSSQFCISVGTNIPAEHNNLQECFDGTIGPETLYKIEDSRVKESAKKSLQLHEALSSI
	9	NEFTFQQELEEMRNASALAAAAAGIAAGRLEEWIFVFAQAADGSSQFCISVGTNIPAEHNNLQECFDGTIGPETLYKIEDSRVKESAKKSLQLHEALSSI
	10	NEFTFQQELEEMRNTSALAAAAAGLAAGRLEEWIFVFAQAADRSSQFCISVGKHIAAEHGNLQECFDGTIGPETLYKIEDSRVKESAKTSLQLHEVLSSI
	11	NEFTFQQELEEMRNASALAAAAAGIAAGRLEEWIFVFAQAAGRSSQFCISVGKHIPAEHGNLQECFDGIIGPETLYKIEDSRVKESAQKSLQLHEVLSSI
	12	NEFTFQQELEEMRNASALAAAAAGIAAGRLEEWIFVFAQAAGGSSQFCISVGKHIPAEHGNLQECFDGIIGPETLYKIEDSRVKESAQKSLQLHEVLSSI
	13	NEFTFQQELEEMRNASALAAAAAGLAAGRLEEWIFVFAQAADGSSQFCISVGKTGPAEYNNLQECFDGTIGPETLYKIEDSRVKESAKTRLLLHEVLSSI
	14	NEFTFQQELEEMRNASALAAAAAGLAAGRLEEWIFVFAQAAGRSSQFCISTGKTGPAEYNNLQECFDGTIGPETLYKIEDSRVKESAKKSLQLHEVLSSI
	15	NEFTFQQELEEMRNASALAAAAAGIAAGRLEEWIFVFAQAAGRSSQFCISVGKTGPAEYNNLQECFDGTIGPETLYKIEDSRVKESAKKSLQLHEVLSSI
	16	NEFTFQQELEEMRNASALAAAAAGLAAGRLEEWIFVFAQAAGRSSQFCISVGKTIPAEHGDLQECFDGTIGPETLYKIEDSRVKESAKKSLQLHEALSSI
	17	NEFTFQQELEEMRNASALAAAAAGIAAGRLEEWIFVFAQAAGRSSQFCISVGKHIPAEHGNLQECFDGIIGPETLYKIEDSRVKESAQKSLQLHEVLSSI
	18	NEFTFQQELEEMRNASALAAAAAGIAAGRLEEWIFVFAQAAGRSSQFCISVGKHIPAEHGNLQECFDGIIGPETLYKIEDSRVKESAQKSLQLHEVLSSI
	19	NEFTFQQELEEMRNASALAAAAAGIAAGRLEEWIFVFAQAGGRSSQFCISTGKTGPAEYNNLQECFDGTIGPETLYKIEDSRVKESAKTSLQLHEVLSSI
	20	NEFTFQQELEEMRNTSALAAAAAGIAAGRLEEWIFVFAQAADRSSQFCISVGKTIPPEHNNLQECFDGTIGPETLYKIEDSRVKESAKKSLQLHEALSSI
	21	NEFTFQQELEEMRNASALAAAAAGIAAGRLEEWIFVFAQAAGRSSQFCISTGKTGPAEYNNLQECFDGTIGPETLYKIEDSRVKESAKTRLLLHEVLSSI
	22	NEFTFQQELEEMRNASALAAAAAGLAAGRLEEWIFVFAQAAGGSSQFCISVGKHIAAEHGNLQECFDGKIGPETLYKIEDSRVKESAQKSLQLHEVLSSI
T. equiperdum	1	NEFTFQQELEEVRNASALAAAAAGLAAGRLEEWIFVFAQAADRSSQFCISTGKTGPAEYNNLQECFDGTIGPETLYKIEDSRVKESAKKSLQLHEVLSSI
	2	NEFTFQQELEEMRNASALAAAAAGLAAGRLEEWIFVFAQAGGRSSQFCISTGKTGPAEYNNLQECFDGTIGPETLYKIEDSRVKESAKTRLLLHEVLSSI
	3	NEFTFQQELKEMRNASALAAAAAGLAAGRLEEWIFVFAQAGGRSSQFCISTGKTGPAEYNNLQECFDGTIGPETLYKIEDSRVKESAKTRLLLHEVLSSI
	4	NEFTFQQELEEMRNASALAAAAAGLAAGRLEEWIFVFAQAAGGSSQFCISVGRTGPAEYNNLQECFDGKIGPETLYKIEDSRVKESAQKSLQLHEVLSSI
	5	NEFTFQQELEEMRNASALAAAAAGLAAGRLEEWIFVFAQAAGGSSQFCISVGRTGPAEYNNLQECFDGKIGPETLYKIEDSRVKESAQKSLQLHEVLSSI
	6	NEFTFQQELEEMRNASALAAAAAGIAAGRLEEWIFVFAQAAGGSSQFCISVGRTGPAEYNNLQECFDGKIGPETLYKIEDSRVKESAQKSLQLHEVLSSI
	7	NEFTFQQELEEMRNASALAAAAAGLAAGRLEEWIFVFAQAGGGSSQFCISVGTAIPPEHKNLQECFDGTIGPETLYKIEDSRVKESAKTRLLLHEVLSSV

ESAG6p type		200 210 220 230 240 250 260 270 280 290
T. b. gambiense	1	SFGSLGVKNIRGGNGRDGCNLVRTDTNGILNGGSPTRHNLTWGGGVMNFGSYQNGSMYVEGGEYGDATEYGAVRWTEDPSKVSIFEDVIRLFARFQEAKN
	2	SFGSLGVKNIRGGNGRDGCNLVRTDTNGILNGGSPTRHNLTWGGGVMNFGSYQNGSMYVEGGEYGDATEYGAVRWTEDPSKVSIFEDVIRLLARFQEAKN
	3	SFGSLGVKNIRGGNGRDGCNLVRTDTNGILNGGSPTRHNLTWGGGVMNFGSYQNGSMYVEGGEYGDATEYGAVRWTEDPSKVSIFEDVIRLFARFQEAKN
	4	SFGSLGAENIRGGNGKDGCNLVRTDNNGILKGGSPTRHNLTWGGGVMNFGSYQNGSMYVEGGEYGDATEYGAVRWTEDPSKVSIFKDVIRLFARFKEAKN
	5	SFGSLGVKNIRGGNGRDGCNLVRTDTNGILNGGSPTRHNLTWGGGVMNFGSYQNGSMYVEGGEYGDATEYGAVRWTEDPSKVSIFEDVIRLFARFQEAKN
	6	SFGSLGVKNIRGGNGRDGCNLVRTDTNGILNGGSPTRHNLTWGGGVMNFGSYQNGSMYVEGGEYGDATEYGAVRWTEDPSKVSIFEDVIRLFARFQEAKN
	7	SFGSLGVKNIRGGNGRDGCNLVRTDTNGILNGGSPTRHNLTWGGGVMNFGSYQNGSMYVEGGEYGDATEYGAVRWTEDPSKVSIFEDVIRLFARFQEAKN
T. b. brucei	1	SFNSLGAESIRGGNGKDGCNLVRTDTDGILNGGSPTRHNLTWGGGVMNFGSYQNGSMYVEGGEYGDATEYGAVRWTEDPSKVSIFKDVIRLFARFQEAKN
	2	SFNSLGAENIRGGNGRDGCNLVRTDTDGVLEGGSVRRHNLTWGGGVMNFGSYQNGSMYVEGGEYGDATEYGAVRWTEDPSKVSIFKDVIRLFARFQEAKN
	3	SFGSLGVKNIRGGNGKDGCNLVRTDTDGVLEGGSPTRHNLTWGGGVMNFGSYQNGSMYVEGGEYGDATEYGAVRWTDDPSKVSIFKDVIRLFARFQEAKN
	5	SFSSLGAENIRGGNGKDGCNLVRTDNNGILKGGSPTRHNLTWGGGVMNFGSYQNGSMYVEGGEYGDATPHGTVRWTEDPNKVSIFKDVIRLFARFKEAKN
	6	SFSSLGAENIRGGNGKDGCNLVRTDNNGILKGGSPTRHNLTWGGGVMNFGSYQNGSMYVEGGEYGDATPHGTVRWTEDPNKVSIFKDVIRLFARFKEAKN
	7	SFGSLGVKNIRGGNGMDGCNLVRTDTDGILAGGSPTRHNLTWGGGVMNFGSYQNGSMYVEGGEYGDATEYGAVRWTKDPSKVSIFKDVIRLFARFQEAKN
	8	SFSSLGVKNIRGGNGRDGCNLVRTDTNGILNGGSPTRHNLTWGGGVMNFGSYQNGSMYVEGGEYGDATEYGAVRWTEDPSKVSIFKDVIRLFARFQEAKN
	9	SFSSLGVKNIRGGNGRDGCNLVRTDTNGILNGGSPTRHNLTWGGGVMNFGSYQNGSMYVEGGEYGDATEYGAVRWTEDPSKVSIFEDLIRLFARFQEAKN
	10	SFGSLGVKNIRGGNGRDGCNLVRTDTDGVLEGGSPTRHNLTWGGGVMNFGSYQNGSMYVEGGEYGDATEYGAVRWTEDPSKVSIFKDVIRLFARFQEAKN
	11	SFNSLGAENIRGGNGRHGCNLVRTDTDGVLEGGSVRRHNLTWGGGVMNFGSYQNGSMYVEGGEYGDATEYGAVRWTEDPSKVSIFKDVIRLFARFQEAKN
	12	SFNSLGAENIRGGNGRHGCNLVRTDTDGVLEGGSVRRHNLTWGGGVMNFGSYQNGSMYVEGGEYGDATEYGAVRWTEDPSKVSIFKDVIRLFARFQEAKN
	13	SFGSLGAENIRGGNGKDGCNLVRTDNNGILKGGSPTRHNLTWGGGVMNFGSYQNGSMYVEGGEYGDATEYGAVRWTEDPSKVSIFKDVIRLFARFKEAKN
	14	SFSSLGVKNIRGGNGKDRCNLVRTDTDGVLEGGSPTRHNLTWGGGVMNFGSYQNGSMYVEGGEYGDATEYGAVRWTEDPSKVSIFKDVIRLFARFQEAKN
	15	SFGSLGVKNIRGGNGKDRCNLVRTDTDGVLEGGSPTRHNLTWGGGVMNFGSYQNGSMYVEGGEYGDATEYGAVRWTEDPSKVSIFKDVIRLFARFQEAKN
	16	SFSSLGVKNIRGGNGRDGCNLVRTDTNGILEGGSPTRHNLTWGGGVMNFGSYQNGSMYVEGGEYGDATEYGAVRWTEDPSKVSIFEDVIRLFARFQEAKN
	17	SFNSLGAENIRGGNGRDGCNLVRTDTDGVLEGGSVRRHNLTWGGGVMNFGSYQNGSMYVEGGEYGDATEYGAVRWTEDPSKVSIFKDVIRLFARFQEAKN
	18	SFNSLGAENIRGGNGRDGCNLVRTDTDGVLEGGSVRRHNLTWGGGVMNFGSYQNGSMYVEGGEYGDATEYGAVRWTEDPSKVSIFKDVIRLFARFQEAKN
	19	SFGSLGVKNIRGGNGRDGCNLVRTDTDGVLEGGSPTRHNLTWGGGVMNFGSYQNGSMYVEGGEYGDATEYGAVRWTEDPSKVSIFKDVIRLFARFQEAKN
	20	SFGSLGVKNIRGGNGKDGCNLVRTDTDGILNGGSPTRHNLTWGGGVMNFGSYQNGSMYVEGGEYGDATEYGAVRWTEDPSKVSIFKDVIRLFARFQEAKN
	21	SFGSLGAENIRGGNGKDGCNLVRTDNNGILKGGSPTRHNLTWGGGVMNFGSYQNGSMYVEGGEYGDATEYGAVRWTEDPSKVSIFKDVIRLFARFQEAKN
	22	SFGSLGVKNIRGGNGRDGCNLVRTDTNGILNGGSPTRHNLTWGGGVMNFGSYQNGSMYVEGGEYGDATEYGAVRWTEDPSKVSIFEDVIRLFARFQEAKN
T. equiperdum	1	SFSSLGAENIRGGNGKDGCNLVRTDNNGILKGGSPTRHNLTWGGGVMNFGSYQNGSMYVEGGEYGDATEYGAVRWTEDPSKVSIFKDVIRLFARFQEAKN
	2	SFGSLGAENIRGGNGKDGCNLVRTDNNGILKGGSPTRHNLTWGGGVMNFGSYQNGSMYVEGGEYGDATEYGAVRWTEDPSKVSIFKDVIRLFARFQEAKN
	3	SFSSLGAENIRGGNGKDGCNLVRTDNNGILKGGSPTRHNLTWGGGVMNFGSYQNGSMYVEGGEYGDATEYGAVRWTEDPSKVSIFKDVIRLFALFQEAKN
	4	SFSSLGAENIRGGNGKDGCNLVRTDNNGILKGGSPTRHNLTWGGGVMNFGSYQNGSMYVEGGEYGDATPHGTVRWTEDPNKVSIFKDVIRLFARFKEAKN
	5	SFSSLGAENIRGGNGKDGCNLVRTDNNGILKGGSPTRHNLTWGGGVMNFGSYQNGSMYVEGGEYGDATPHGTVRWTEDPNKVSIFKDVIRLFARFKEAKN
	6	SFSSLGAENIRGGNGKDGCNLVRTDNNGILKGGSPTRHNLTWGGGVMNFGSYQNGSMYVEGGEYGDATPHGTVRWTEDPNKVSIFKDVIRLFARFKEAKN
	7	SFGSLGAENIRGGNGKDGCNLVRTDNNGILKGGSPTRHNLTWGGGVMNFGSYQNGSMYVEGGEYGDATEYGAVRWTEDPSKVSIFKDVIRLFARFQEAKN

ESAG6p type

```
                 300       310       320       330       340       350       360       370       380       390         402
T. b. gambiense
 1  AVMRRIKTTVDELTKCIGQKEAELTNDQIYEEFIWETIHRLELSKRVSEQPSLGEEEETILKSNYTAEPVRGPFTGAGSNTVAVHLSVSTAALCCSVLLLGVL
 2  AVMRRIKTTVDELTKCIGQKEAELTNDQIYEEFIWETIHRLELSKRVSEQPSLGEEEETILKSNYTAEPVRGPFTGAGSNTVAVHLSVSTAALCCSVLLLGVL
 3  AVMRRIKTTVDELTKCIGQKEAELTNDQIYEEFIWETIHRLELSKRVSEQPSLGEEEETILKSNYTAESVRGPFTGAGSNTVAVHLSVSTAALCCSVLLLGVL
 4  AVMTKIKTIVDELTKCIGQKEAELTNDQIYEEFIWETISRLELSKRVSEQPSLGEEEETIVKSNYTAEPVRGPFTGAGANTVAVQSSVSTAALCCSVLLLGVL
 5  AVMTKIKTTVDELTKCIGQKEAELTNDQLYEEFIWETIHRLELSKRVSEQPSLGEEEETILKSNYTAEPVRGPFTGAGSNTVAVHLSVSTAALCCSVLLLGVL
 6  AVMRRIKTTVDELTKCIGQKEAELTNDQIYEEFIWETIHRLELSKRVSEQPSLGEEEETILKSNYTAEPVRGPFTGAGSNTVAVHLSVSTAALCCSVLLLGVL
 7  AVVRRIKTTVDELTKCIGQKEAELTNDQIYEEFIWETIHRLELSKRVSEQPSLGEEEETILKSNYTAEPVRGPFTGAGSNTVAVHLSVSTAALCCSVLLLGVL

T. b. brucei
 1  AVMKKIKTTVDELTKCIGQKEAELTNDQLYEEFIWETINRLELSKRVSEQSAFGEEEETIVKFNYTAEPVRGPFTVAGANAAAIHLSVSTAALCRSALLLGVL
 2  EVMNKIKTTVDELAKCIGQKEVELTDDQLYEEFIWETIHRLELSKRVSEQLSLGEEEETILKSNYTAEPVRGPFTVAGSNTVAVHLSVSTAALCFSVLLLGVL
 3  AVMKKIKTTVDELTKCVGQKEAELTNDQLYEEFIWETINRLELSKRVSEQSAFGEEEETIVKFNYTAEPVRGPFTVAGANAAAIHLSVSTAALCRSALLLGVL
 5  AVMTKIKTTVDELTKCIGQKEAELTNDQLYEEFIWETINRLELSKRVSEQPSLGEEEETILKSNYTAEPVRGPFTGAGANTVAVQSSVSTAALCCSVLLFGVL
 6  AVMTKIKTTVDELTKCIGQKEAELTNDQLYEEFIWETINRLELSKRVSEQPSLGEEEETILKSNYTAEPVRGPFTGAGANTVAVQSSVSTAALCCSVLLFGVL
 7  AVMNKIKTTVDELTKCIGHKEAELTDYQLYEEFIWETIHRLELSKRVSEQPSLGEQEETILKSNYTAEPVRGPFTGAGADAAAIQSSVSTAALCCLALLLGVM
 8  AVMRRIKTTVDELTKCIGQKEAELTNDQIYEEFIWETINRLELSKRMSEQPTLGEEEETILKSNYTAEPVRGPFTGAGANTVALHLSVSTAALCCSVLLLGVL
 9  AVMKKIKTTVDELTKCIGQKEAELTNDQIYEEFIWETINRLELSKRMSEQSAFGEEEETILKSNYTAEPVRGPFTGAGSNTVAVHLSFSTAALCCSVLLLGVL
10  AVMTKIKTTVDELTKCIGQKEAELTNDQLYEEFIWETINRLELSKRVSEQSAFGEEEETIVKFNYTAEPVRGPFTVAGANAAAIHLSVSTAALCRSALLLGVL
11  EVMNKIKTTVDELAKCIGQKEVELTDDQLYEEFIWETIHRLELSKRVSEQLSLGEEEETILKSNYTAEPVRGPFTVAGSNAAAVHLSVSTAALCFSVLLLGVL
12  EVMNKIKTTVDELAKCIGQKEVELTDDQLYEEFIWETIHRLELSKRVSEQLSLGEEEETILKSNYTAEPVRGPFTVAGSNAAAVHLSVSTAALCFSVLLLGVL
13  AVMTKIKTTVDELTKCIGQKEAELTNDQIYEEFIWETINRLELSKRVSEQPSLGEEEETILKSNYTAEPVRGPFTGAGANTVAVQSSVSTAALCCSVLLLGVL
14  AVMKKIKTTVDELTKCIGQKEAELTNDQLYEEFIWETINRLELSKRVSEQSAFGEEEETIVKFNYTAEPVRGPFTVAGANAAAIHLSVSTAALCRSALLLGVL
15  AVMKKIKTTVDELTKCIGQKEAELTNDQLYEEFIWETINRLELSKRVSEQSAFGEEEETIVKFNYTAEPVRGPFTVAGANAAAIHLSVSTAALCRSALLLGVL
16  EVMNKIKTTVDELAKCIGQKEVELTDDQLYEEFIWETIHRLELSKRVSEQPSLGEEEETILKSNYTAEPVRGPFTGAGSNTVAVHLSVSTAALCRSALLLGVL
17  EVMNKIKTTVDELAKCIGQKEVELTDDQLYEEFIWETIHRLELSKRVSEQLSLGEEEETILKSNYTAEPVRGPFTVAGSNAAAVHLSVSTAALCFSVLLLGVL
18  EVMNKIKTTVDELAKCIGQKEVELTDDQLYEEFIWETIHRLELSKRVSEQLSLGEEEETILKSNYTAEPVRGHFTVAGSNAAAVHLSVSTAALCFSVLLLGVL
19  AVMTKIKTTVDELTKCIGHKEAELTNDQLYEEFIWETINRLELSKRVSEQSAFGEEEETIVKFNYTAEPVRGPFTVAGANAAAIHLSVSTAALCRSALLLGVL
20  AVMKKIKTTVDELTKCTGQKEAELTNDQLYEEFIWETINRLELSKRVSEQSAFGEEEETILKSNYTAEPVRGPFTVAGSNAVAIHLSVSTAALCRSALLLGVL
21  AVMKKIKTTVDELTKCIGQKEAELTNDQIYEEFIWETINRLELSKRVSEQPSLGEEEETILKSNYTAEPVRGPFTGAGANTVAVQSSVFTAALCCSALLLGVL
22  AVMRRIKTTVDELTKCIGQKEAELTNDQIYEEFIWETIHRLELSKRVSEQPSLGEEEETILKSNYTAEPVRGPFTGAGSNTVAVHLSVSTAALCCLALLLGVL

T. equiperdum
 1  AVMTKIKTTVDELTKCIGQKEAELTNDQLYEEFIWETINRLELSKRVSEQPSLGEEEETILKSNYTAEPVRGPFTGAGANTVAVQSSVSTAALCCSALLLGVL
 2  AVMTKIKTTVDELTKCIGQKEAELTNDQVYEEFIWETINRLELSKRVSEQPSLGEEEETILKSNYTAEPVRGPFTGAGANTVAVQSSVSTAALCCSVLLFGVL
 3  AVMTKIKTTVDELTKCIGQKEAELTNDQLYEEFIWETINRLELSKRVSEQPSLGEEEETILKSNYTAEPVRGPFTGAGANTVAVQSSVSTAALCCSVLLLGVL
 4  AVMTKIKTTVDELTKCIGQKEAELTNDQLYEEFIWETINGLELSKRVSEQPSLGEEEETILKSNYTAEPVRGPFTGAGANTVAVQSSVSTAALCCSVLLFGVL
 5  AVMTKIKTTVDELTKCIGQKEAELTNDQLYEEFIWETINRLELSKRVSEQPSLGEEEETILKSNYTAEPVRGPFTGAGANTVAVQSSVSTAALCCSVLLFGVL
 6  AVMTKIKTTVDELTKCIGQKEAELTNDQLYEEFIWETINRLELSKRVSEQPSLGEEEETILKSNYTAEPVRGPFTGAGANTVAVQSSVSTAALCCSVLLFGVL
 7  AVMTKIKTTVDELTKCIGQKEAELTNDQVYEEFIWETINRLELSKRVSEQPSLGEEEETILKSNYTAEPVRGPFTGAGANTVAVQSSVSTAALCCSVLLFGVL
```
