# Supplementary material for: Isolation and analysis of the genetic diversity of repertoires of VSG expression site containing telomeres from Trypanosoma brucei gambiense, T. b. brucei and T. equiperdum
Source: BMC Genomics. 2008 Aug 12;9:385. doi: 10.1186/1471-2164-9-385 (PMC2533676; doi:10.1186/1471-2164-9-385)
Supplement: Additional file 4 — Sup. Figure 4. Sequence alignments of ESAG2 sequences analysed in this manuscript. [file 1471-2164-9-385-S4.pdf]

ESAG2p type

Species	No.	Sequence (positions 1–99)
T. b. gambiense	1	MRYEIVIAFGVFAAVLSPSFQSDLGSFKWSTELIDWSKVTEYIDESYKHHNAGEFESLCKIYRITQAEAPQPSFKNREKEREIMSKLEEMVREVEAAGG
	2	MRYKIVFTVGLFAAVLSPSFQSDLGSFKWSTELIDWSKVTEYIDESYKHHNAGEFETLCKIYRITQAEAPQPSFENREKENEILSKLEEMVREVEAAGG
	3	MRYKIVFTVGLFAAVLSPSFQSDLGSFKWSTELIDWSKVTEYIDESYKHHNAGEFETLCKIYRITQAEAPQPSFENREKENEILSKLEEMVREVEAAGG
	4	MRYKIVFTVGLFAAVLSPSFQSDLGSFKWSTELIDWSKVTEYIDESYKHHNAGEFETLCKIYRITQAEAPQPSFENREKENEILSKLEEMVREVEAAGG
	5	MRYKIVIAVGVFVAVLSPSFQSDLGSFKWSTELIDWSKVTEYIDESYKHHNAGEFETLCKIYRITQAEAPQPSFENREREREIMSKLEEMVREVEAAGG
	6	MRYKIVIAVGVFVAVLSPSFQSDLGSFKWSTELIDWSKVTEYIDESYKHHNAGEFETLCKIYRITQAEAPQPSFKNREKEREIMSKLEEMVTETEAVGG
	7	MRYKIVFTVGLFAAVLSPSFQSDLGSFKWSTELIDWSKVTEYIDESYKHHNAGEFETLCKIYRITQAEAPQPSFENREKENEILSKLEEMVREVEAAGG
	8	MRYKIVFTVGLFAAVLSPSFQSDLGSFKWSTELIDWSKVTEYIDESYKHHNAGEFETLCKIYRITQAEAPQPSFENREKENEILSKLEEMVREVEAAGG
T. b. brucei	1	MRCEIVIAVGVFVAVLSPS-----FQSDLGSFKWSTELIDWSKITDYIGDGYKHHNAGEFETLCKIYRITQAEAPQPSFENREKENEILNKLEEMVRET
	2	MRYEIVIAVGLFAAVLYSSSEEAVDDGCTRQARVTNGRPDWDTITECVSGDYMYHNTGDFDVLCKIYRITQAELPQPSFKNREKEGEIMSKLEEMVREV
	3	MRYETVFTVGLFVAVLSPS-----FQSDLGSFKWSTELIDWSKVTEYIDESYKHHNAGEFETLCKIYRITQAEAPQPFFENREKENEILNKLEEMVRET
	4	MRCETVLTIGLFVAVLSHCDGEYIDDECTRVARVTSDRADWETIRECVSGDYMYHNTVDFDVLCKIYRITQAELPQPSFKNRDKEGEIMNKLEEMVREV
	5	MRYEIVIAVGVFVAVLCSSSEEAVDDECTRDARVTNGTPDWDTITECVSGDYMYHNTGDFDVLCKIYRITQAELPQPSFKNREKEGEIMSKLEEMVREV
	6	MRYEIVFVVGLFAAVLYSSSEEAVDDECTRDARVTNGTPDWDTITECVSGDYMYHNTGDFDVLCKIYRITQAELPQPSFKNREKEGEIMSKLEEMVREV
	7	MRCEIVFVVGLFAAVLYSSSEEYMDDECTRDARVTNGTPDSDTITECVSGDYLYHNTGDFDVLCKIYRITQAELPQPSFKNREKEGEIMSKLEEMVREV
	8	MRYEIVIAVGLFAAVLYSSSEEAVHDECTREARVTNGRPDWDTITECVSGDYMYHNTGDFDVLCKIYRITQAELPQPSFKNREKEGEIMSKLEEMVREV
	9	MRYEIVIAVGLFAAVLYSSSEEAVHDECSRDARVTNDTPDSDTITECVSGDYLYHNTGDFDVLCKIYRITQAELPQPSFKNREKEREIMSKLEEMVREV
	10	MRCEIVFVVGLFAAVLSPS-----FQSDLGSFKWSTELIDWSKVTEYIDESYKHHNAGEFETLCKIYRITQAEAPQPSFKNREKEGEIMSKLEEMVRET
	11	MRCEIVFVVGLFAAVLSPS-----FQSDLGSFKWSTELIDWSKVTEYIDESYKHHNAGEFETLCKIYRITQAEAPQPSFKNREKEGEIMSKLEEMVRET
	12	MRYKIVIAVGVFVAVLSPS-----FQSDLGSFKWSTELIDWSKVTEYIDESYKHHNTGDFDVLCKIYRITQAEAPQPSFENREKENEILNKLEEMVRET
	13	MRYEIVIAVGVFVAVLCSSSEEAVHDECTREARVTNGRPDWETITECVSGDYMYHNTGDFDVLCKIYRITQAELPQPSFKNREKEGEIMSKLEEMVREV
	14	MRYEIVFTVGLFAAVLSPS-----FQSDLRSFKWSTELIDWSKITDYIGDGYKHHNAGEFETLCKIYRITQAEAPQPSFENREKEGEIMSKLEEMVREV
	15	MRCEIVFVVGLFAAVLYSSSEEAVDDECTRDARVTNGTPDSDTITECVSGDYLYHNTGDFDVLCKIYRITQAELPQPSFKNREKEGEIMSKLEEMVREV
	16	MRYETVFTVGLFAAVLSPS-----FQSDLGSFKWSTELIDWSKVTEYIDESYKHHNAGEFETLCKIYRITQAEAPQPSFENREKENEILNKLEEMVRET
	17	MRYEIVIAVGLFAAVLYSSSEEAVDDECTRQARVTNGRPDWDTITECVSGDYMYHNTGDFDVLCKIYRITQAELPQPSFKNREKEGEIMSKLEEMVREV
T. equiperdum	1	MRYKIVIAVGVFVAVLSPS-----FQSDLGSFKWSTELIDWSKVTEYIDESYKHHNAGEFETLCKIYRITQAEAPQPSFENREREREIMSKLEEMVREV
	2	MRYKIVFTVGLFAAVLSPS-----FQSDLGSFKWSTELIDWSKVTEYIDESYKHHNAGEFETLCKIYRITQAEAPQPSFENREKENEIMNKLEEMVREV
	3	MRYEIVIAVGLFAAVLCSSSEEAVDDECLRDARVTNDTPDSDTITECVSGDYLYHNTGDFDVLCKIYRITQAELPQPSFKNREKEGEIMSKLEEMVREV
	4	MRYKIVFTVGLFAAVLSPS-----FQSDLGSFKWSTELIDWSKVTEYIDESYKHHNAGEFETLCKIYRITQAEAPQPSFENREKENEIMNKLEEMVREV

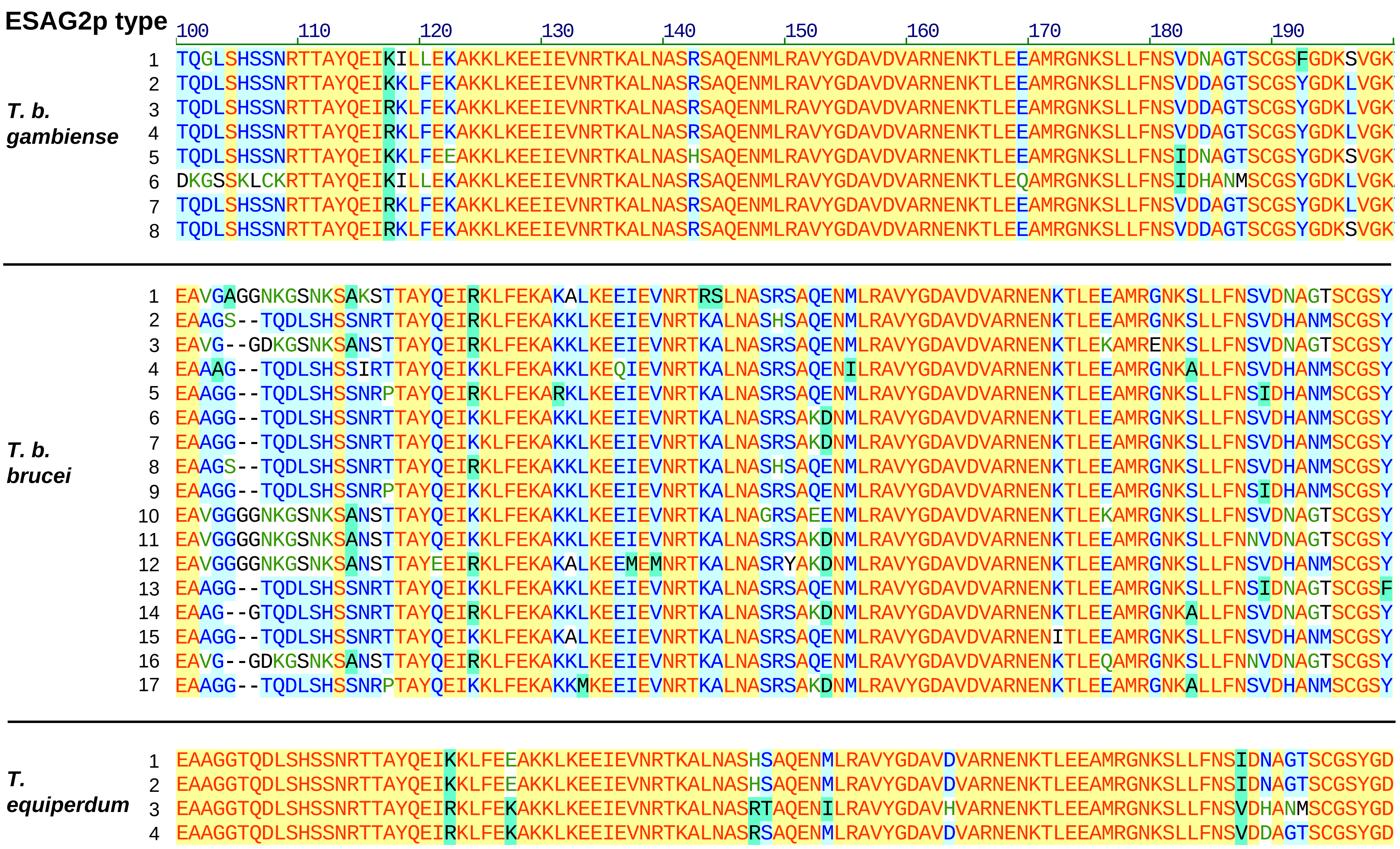
ESAG2p type
100 110 120 130 140 150 160 170 180 190
T. b. gambiense
1 TQGLSHSSNRTTAYQEIKILLEKAKKLKEEIEVNRTKALNASRSAQENMLRAVYGDAVDVARNENKTLEEAMRGNKSLLFNSVDNAGTSCGSFGDKSVGK
2 TQDLSHSSNRTTAYQEIKKLFEKAKKLKEEIEVNRTKALNASRSAQENMLRAVYGDAVDVARNENKTLEEAMRGNKSLLFNSVDDAGTSCGSYGDKLVGK
3 TQDLSHSSNRTTAYQEIRKLFEKAKKLKEEIEVNRTKALNASRSAQENMLRAVYGDAVDVARNENKTLEEAMRGNKSLLFNSVDDAGTSCGSYGDKLVGK
4 TQDLSHSSNRTTAYQEIRKLFEKAKKLKEEIEVNRTKALNASRSAQENMLRAVYGDAVDVARNENKTLEEAMRGNKSLLFNSVDDAGTSCGSYGDKLVGK
5 TQDLSHSSNRTTAYQEIKKLFEEAKKLKEEIEVNRTKALNASHSAQENMLRAVYGDAVDVARNENKTLEEAMRGNKSLLFNSIDNAGTSCGSYGDKSVGK
6 DKGSSKLCKRTTAYQEIKILLEKAKKLKEEIEVNRTKALNASRSAQENMLRAVYGDAVDVARNENKTLEQAMRGNKSLLFNSIDHANMSCGSYGDKLVGK
7 TQDLSHSSNRTTAYQEIRKLFEKAKKLKEEIEVNRTKALNASRSAQENMLRAVYGDAVDVARNENKTLEEAMRGNKSLLFNSVDDAGTSCGSYGDKLVGK
8 TQDLSHSSNRTTAYQEIRKLFEKAKKLKEEIEVNRTKALNASRSAQENMLRAVYGDAVDVARNENKTLEEAMRGNKSLLFNSVDDAGTSCGSYGDKSVGK
T. b. brucei
1 EAVGAGGNKGSNKSAKSTTAYQEIRKLFEKAKALKEEIEVNRTRSLNASRSAQENMLRAVYGDAVDVARNENKTLEEAMRGNKSLLFNSVDNAGTSCGSY
2 EAAGS--TQDLSHSSNRTTAYQEIRKLFEKAKKLKEEIEVNRTKALNASHSAQENMLRAVYGDAVDVARNENKTLEEAMRGNKSLLFNSVDHANMSCGSY
3 EAVG--GDKGSNKSANSTTAYQEIRKLFEKAKKLKEEIEVNRTKALNASRSAQENMLRAVYGDAVDVARNENKTLEKAMRENKSLLFNSVDNAGTSCGSY
4 EAAAG--TQDLSHSSIRTTAYQEIKKLFEKAKKLKEQIEVNRTKALNASRSAQENILRAVYGDAVDVARNENKTLEEAMRGNKALLFNSVDHANMSCGSY
5 EAAGG--TQDLSHSSNRPTAYQEIRKLFEKARKLKEEIEVNRTKALNASRSAQENMLRAVYGDAVDVARNENKTLEEAMRGNKSLLFNSIDHANMSCGSY
6 EAAGG--TQDLSHSSNRTTAYQEIKKLFEKAKKLKEEIEVNRTKALNASRSAKDNMLRAVYGDAVDVARNENKTLEEAMRGNKSLLFNSVDHANMSCGSY
7 EAAGG--TQDLSHSSNRTTAYQEIKKLFEKAKKLKEEIEVNRTKALNASRSAKDNMLRAVYGDAVDVARNENKTLEEAMRGNKSLLFNSVDHANMSCGSY
8 EAAGS--TQDLSHSSNRTTAYQEIRKLFEKAKKLKEEIEVNRTKALNASHSAQENMLRAVYGDAVDVARNENKTLEEAMRGNKSLLFNSVDHANMSCGSY
9 EAAGG--TQDLSHSSNRPTAYQEIKKLFEKAKKLKEEIEVNRTKALNASRSAQENMLRAVYGDAVDVARNENKTLEEAMRGNKSLLFNSIDHANMSCGSY
10 EAVGGGGNKGSNKSANSTTAYQEIKKLFEKAKKLKEEIEVNRTKALNAGRSAEENMLRAVYGDAVDVARNENKTLEKAMRGNKSLLFNSVDNAGTSCGSY
11 EAVGGGGNKGSNKSANSTTAYQEIKKLFEKAKKLKEEIEVNRTKALNASRSAKDNMLRAVYGDAVDVARNENKTLEEAMRGNKSLLFNNVDNAGTSCGSY
12 EAVGGGGNKGSNKSANSTTAYEEIRKLFEKAKALKEEMEMNRTKALNASRYAKDNMLRAVYGDAVDVARNENKTLEEAMRGNKSLLFNSVDHANMSCGSY
13 EAAGG--TQDLSHSSNRTTAYQEIKKLFEKAKKLKEEIEVNRTKALNASRSAQENMLRAVYGDAVDVARNENKTLEEAMRGNKSLLFNSIDNAGTSCGSF
14 EAAG--GTQDLSHSSNRTTAYQEIRKLFEKAKKLKEEIEVNRTKALNASRSAKDNMLRAVYGDAVDVARNENKTLEEAMRGNKALLFNSVDNAGTSCGSY
15 EAAGG--TQDLSHSSNRTTAYQEIKKLFEKAKALKEEIEVNRTKALNASRSAQENMLRAVYGDAVDVARNENITLEEAMRGNKSLLFNSVDHANMSCGSY
16 EAVG--GDKGSNKSANSTTAYQEIRKLFEKAKKLKEEIEVNRTKALNASRSAQENMLRAVYGDAVDVARNENKTLEQAMRGNKSLLFNNVDNAGTSCGSY
17 EAAGG--TQDLSHSSNRPTAYQEIKKLFEKAKKMKEEIEVNRTKALNASRSAKDNMLRAVYGDAVDVARNENKTLEEAMRGNKALLFNSVDHANMSCGSY
T. equiperdum
1 EAAGGTQDLSHSSNRTTAYQEIKKLFEEAKKLKEEIEVNRTKALNASHSAQENMLRAVYGDAVDVARNENKTLEEAMRGNKSLLFNSIDNAGTSCGSYGD
2 EAAGGTQDLSHSSNRTTAYQEIKKLFEEAKKLKEEIEVNRTKALNASHSAQENMLRAVYGDAVDVARNENKTLEEAMRGNKSLLFNSIDNAGTSCGSYGD
3 EAAGGTQDLSHSSNRTTAYQEIRKLFEKAKKLKEEIEVNRTKALNASRTAQENILRAVYGDAVHVARNENKTLEEAMRGNKSLLFNSVDHANMSCGSYGD
4 EAAGGTQDLSHSSNRTTAYQEIRKLFEKAKKLKEEIEVNRTKALNASRSAQENMLRAVYGDAVDVARNENKTLEEAMRGNKSLLFNSVDDAGTSCGSYGD

```
ESAG2p type  200       210       220       230       240       250       260       270       280       290

T. b. gambiense
 1  TLINDFFCLCVGEPIVITEEIK------RKKNLEGDGDNNNIYNGFNCPCRDEIRRPENGNWTMMAESCEGDSQGYCDPAKIKYNHTEAWDVISKACVYK
 2  TLINDFFCLCVGEAINVTEDMR--QKKEKEEEKEKGKYSDSVYNGFNCPCRDAIRRPRSGSWTMMAEYCEGNSS-PCNPDNIKYNYTDAWDVISKACVYK
 3  TLINDFFCLCVGETINVTGEIIKEEKKKEEKEKEEGKYSDSVYNGYNCPCRDAIRRPRSGSWTMMAEYCEGNSS-PCNPDNIKYNYTDAWDVISKACVYK
 4  TLINDFFCLCVGEAINVTGEIIKEEKKKEEKEKEEGKYSDSVYNGYNCPCRDAIRRPRSGSWTMMAEYCEGNSS-PCNPDNIKYNYTDAWDVISKACVYK
 5  TLINDFFCLCVGEAINVTGEIIKEEKKKEEKEKEEGKYSDSVYNGYNCPCRDAIRRPRSGSWTMMAEYCEGNSS-PCYPEDIKYNYTEAWDVISEACVYK
 6  ALINDFFCLCVGEAHKVQTGQS-------APDGDYNLTIIEIYNRLNCPCKDAIRRPRSGSWTMMAEYCEGNSS-PCNPDNIKYNYTDAWDVISKACVYK
 7  TLINDFFCLCVGEAINVTGEIIKEEKKKGEKEKEKGKYSDSVYNGYNCPCRDAIRRPRSGSWTMMAEYCEGNSS-PCNPDNIKYNYTDAWDVISKACVYK
 8  TLINDFFCLCVGEAINVTEDMR--QKKEKEEEKEKGKYSDSVYNGFNCPCRDAIRRPRSGSWTMMAEYCEGNSS-PCYPEDIKYNYTEAWDVISEACVYK

T. b. brucei
 1  GEKLVGMTLINDFFCLCVGEAINVTDEMRQ--------------------------------------KKEEEEKKEGGKYSDSVYNGFNCPCRDEIRRP
 2  GDKLVGKTLINDFFCLCVGEAD-------------------------------------------KVQTGQSATDGDYKLTLIEIYNRLNCPCKDAIRRP
 3  GDKLVGKTLINDFFCLCVGEAINVTEDMK---------------------------------------QKKEEKKKEEGKYSDSVYNGFNCPCRDGIRRP
 4  GDKLVGKTLINDFFCLCVGEAINVTGEIIKEEKKKEEKKKE------------EKEKEEKEKEEGVLKIGPNATDGDYNLTMIEIYNRLNCPCKDAIRRP
 5  GDKLVGKTLINDFFCLCVGEAHKVQTG-------------------------------------------QSATDGDDNLTLIEIYNRLNCPCKDAIRRP
 6  GDKLVGKTLINDFFCLCVGEAISVTGEIIKEEKKKEEKEKEVKEEEVKEEEEEEKEEKEKKKEVEEVQSVQSATDGDYNLTIIEIYNRLNCPCKDAIRRP
 7  GDKLVGKTLINDFFCLCVGEAH-------------------------------------------KVQTGQSGTDGDYNLTIIEIYNRLNCPCKDAIRRP
 8  GDKLVGKTLINDFFCLCVGEAISVTGDMKEEKEEEEE----------------EKKEEEKEKEEEEVQSVQSATAGDYKLTLIEIYNRLNCPCKDAIRRP
 9  GDKLVGKTLINDFFCLCVGEAHKVQTG-------------------------------------------QSATDGDDNLTLIEIYNRLNCPCKDAIRRP
10  GDKLVGKTLINDFFCLCVGEAINVTEDMGQ--------------------------------------KKKEEQRRKEGKYSDSVYNGFNCPCRDAIRRP
11  GDKLVGKTLINDFFCLCVGEAINVTEEMK---------------------------------------KKKEEEEKGGGKYSDSVYNGFNCPCRDEIRRP
12  GDKLVGKTLINDFFCLCVGEAISVTGEIIKEEKKKEE-----------------E-----EVKEEEVQSVQSATDGDYNLTMIEIYNRLNCPCKDAIRRP
13  GDKLVGKTLINDFFCLCVGEAH-------------------------------------------KVQTGQSAADGDYKLTMIEIYNRLNCPCKDAIRRP
14  GDKSVGKTLINDFFCLCVGEAINVTDEMRQ--------------------------------------KKKEEEKKEKGKYSDSVYNGFNCPCRDAIRRP
15  GDKLVGKTLINDFFCLCVGEAH-------------------------------------------KVQTGQSGTDGDYNLTIIEIYNRLNCPCKDAIRRP
16  GEKFVGKTLINDFFCLCVGEAINVTGEIIKE------------------------------------EKKKEEKEKEEGKYSDSVYNGYNCPCRDAIRRP
17  GDKLVGKTLINDFFCLCVGEAIDIVKKEERRKEE---------------------------KEEEEVQSVQSATAGDYNLTMIEIYNRLNCPCKDAIRRP

T. equiperdum
 1  KSVGKTLINDFFCLCVGEAINVTEDMR--QKKEKEEEKEKGKYSDSVYNGFNCPCRDAIRRPRSGSWTMMAEYCEGNSSPCYPEDIKYNYTEAWDVISEA
 2  KSVGKTLINDFFCLCVGEAINVTGEIIKEEKKKEEKEKEEGKYSDSVYNGYNCPCRDAIRRPRSGSWTMMAEYCEGNSSPCNPDNIKYNYTEAWDVISEA
 3  KLVGKTLINDFFCLCVGEAHKVQTGQS-------ATDGDDNLTMIEIYNRLNCPCKDAIRRPKSGSWTMMAEHCDGGERICDPENVTYGHTEAWDVISEA
 4  KLVGKTLINDFFCLCVGEAINVTGEIIKEEKKKEEKEKEEGKYSDSVYNGYNCPCRDAIRRPRSGSWTMMAEYCEGNSSPCNPDNIKYNYTDAWDVISKA
```

ESAG2p type

```
             300       310       320       330       340       350       360       370       380       390
```

T. b. gambiense

```
1  NIGSNVKTLKSALAEFDDLVNLEQDKYQVKGILGYVRTDHNKNHACTGHTAGFTCVSYKHTLENGGIPWYNHLTNATEQLQEMTKYAKEADSHLHELEEY
2  NVASNVKTLKSVLAQFNALVNLEQDKYQVKGIFGYVKTENNDNRTCTGHTAGFTCVSYSYTLENGGIPWYNRLSNATEQLQEMDKYAKEADSHLHELEEY
3  NVASNVKTLKSVLAQFNALVNLEQDKYQVKGIFGYVKTENNDNRTCTGHTAGFTCVSYSYTLENGGIPWYNRLSNATEQLQEMDKYAKEADSHLHELEEY
4  NVASNVKTLKSVLAQFNALVNLEQDKYQVKGIFGYVKTENNDNRTCTGHTAGFTCVSYSYTLENGGIPWYNRLSNATEQLQEMDKYAKEADSHLHELEEY
5  NIASNVKTLKSALAQFDSLVNLEQDKYQVRGIFGYVKTEHNDNRTCTGHTAGFTCVSYSYTLENGGIPWYNHLTNATEQLQEMDKYAKEADSHLHELEEY
6  NVASNVKTLKSALAEFDDLVNLEQDKYQVKGILGYVKTENNDNRTCTGHTAGFTCVSYSYTLENGGIPWYNRLSNATEQLQEMDMYAKEADSHLHELEEY
7  NVASNVKTLKSVLAQFNALVNLEQDKYQVKGIFGYVKTENNDNRTCTGHTAGFTYVSYSYTLENGGIPWYNRLSNATEQLQEMDKYAKEADSHLHELEEY
8  NIASNVKTLKSALAQFDSLVNLEQDKYQVRGIFGYVKTEHNDNRTCTGHTAGFTCVSYSYTLENGGIPWYNRLSNATEQLQEMDKYAKEADSHLHELEEY
```

T. b. brucei

```
1  KNGSWTMMAEYCEGNSSPCNPEDIKYNHTEAWDVISKACVYKSVASNVKTLKSALAEFDDLVNLEQDKYQVRGIFGYVKTENNDNRTCTGHTAGFTCVSY
2  KSGSWTMMAEHCDGDGRLCDPENVTYGHTEAWDVISKACVYKNVASNVKTLKSALAQFDALVNLEQDEYQVKGVLGYVRTDHNKNHACTGHTAGFTCVSY
3  KNGSWTMMANFCEGNSSPCNPEDIKYNHTEAWDVISKTCVYKNIGSNVKTLKSALAEFDDLVNLEQDKYQVKGILGYVKTENNDNRTCTGHTAGFTCVSY
4  KSGSWTMMAEYCDGDGRLCDPENVTYGHTEAWDVISEACVYKNIGSNVKTLKSALAQFDDLVNLEQDKYQVKGILGYVRTDHNKNHACTGHTAGFTCVSY
5  KSGSWTMMAEHCDGDGRICDPENVTYGHTEAWDVISEACVYKNIGSNVKTLKSALAEFDDLVNLEQDKYQVKGIFGYVRTDHNKNHACTGHTAGFTCVSY
6  KSGSWTMMAEHCDGDGRLCDPENVTYGHTEAWDVISKTCVYKNIGSNVKTLKSALAEFDDLVNLEQDKYQVKGILGYVRTDHNKNHACTGHTAGFTCVSY
7  KSGSWTMMAEYCKGGEDICNPENVTYGHTEAWDVISEACVYKNIGSNVKTLKSALAEFDDLVNLEQDKYQVKGILGYVRTDHNKNHACTGHTAGFTCVSY
8  KSGSWTMMAEHCDGDGRLCDPENVTYGHTEAWDVISKACVYKNVASNVKTLKSALAQFDALVNLEQDEYQVKGILGYVRTDHNKNHACTGHTAGFTCVSY
9  KSGSWTMMAEHCDGVERICDPENVTYGHTEAWDVISKTCVYKNVASNVKTLKSALAEFDDLVNLEQDKYQVKGILGYVRTDHNKNHACTGHTAGFTCVSY
10 KNGSWTMMANYCEGNSSPCNPEDIKYNYTEAWDVISKACVYKNIGSNVKTLKSALAEFDDLVNLEQDKYQVKGIFGYVKTENNDNRTCTGHTAGFTCVSY
11 KNGSWTMMAEYCEGNSSPCNPEDIKYNYTEAWDVISKACVYKNIGSNVKTLKSALAEFDDLVNLEQDKYQVKGIFGYVKTENNDNRTCTGHTAGFTCVSY
12 KSGSWTMMAEHCDGDGRICDPENVTYGHTEAWDVISKACVYKNVASNVKTLKSALAEFDDLVNLEQDKYQVKGILGYVRTDHNKNHACTSHTAGFTCVSY
13 KSGSWTMMAEHCDGDGRLCDPENVTYGHTEAWDVISKACVYKNVASNVKTLKSALAQFDALVNLEQDEYQVKGILGYVRTDHNKNHACTGHTAGFTCVSY
14 KNGSWTMMANYCEGNSNPCSPDNIKYNYTDAWDVISKTCVYKNIGSNVKTLKSALAEFDDLVNLEQDKYQVKGILGYVKTENNDNRTCTGHTAGFTCVSY
15 KSGSWTMMAEYCKGGEDICNPENVTYGHTEAWDVISKTCVYKNIGSNVKTLKSALAEFDDLVNLEQDKYQVKGILGYVRTDHNKNHACTGHTAGFTCVSY
16 RSGSWTMMAEYCEGNSSPCNPDNIKYNYTDAWDVISKACVYKNVASNVKTLKSVLAQFNALVNLEQDKYQVKGIFGYVKTENNDNRTCTGHTAGFTCVSY
17 KSGSWTMMAEHCDGDGRMCDPENVTYGHTEAWDVISKTCVYKNIGSNVKTLKSALAEFDDLVNLEQDKYQVKGILGYVRSDHNKNHACTGHTAGFTCVSY
```

T. equiperdum

```
1  CVYKNIASNVKTLKSALAQFDSLVNLEQDKYQVRGIFGYVKTEHNDNRTCTGHTAGFTCVSYSYTLENGGIPWYNHLTNATEQLQEMDKYAKEADSHLHE
2  CVYKNIASNVKTLKSALAQFDSLVNLEQDKYQVRGIFGYVKTENNDNRTCTGHTAGFTCVSYSYTLENGGIPWYNHLTNATEQLQEMDKYAKEADSHLHE
3  CVYKNIGSNVKTLKSALAEFDDLVNLEQDKYQVKGILGYVRTDHNKNHACTGHTAGFTCVSYSYTLENGGIPWYNRLSNATEQLQEMDKYAKEADSHLHE
4  CVYKNVASNVKTLKSVLAQFNALVNLEQDKYQVKGIFGYVKTENNDNRTCTGHTAGFTCVSYSYTLENGGIPWYNHLTNATEQLQEMDKYAKEADSHLHE
```

ESAG2p type

```
     400       410       420       430       440       450       460       470       480       490        503
```

T. b. gambiense

```
1  EDEAEEIFLEAKLGGDAELWKSSQGKGYSGGDDTVVNNDGLTHLNMETGGFTLLVLSLICTL
2  EDEAEEIFLEAKLGGDAELWKSSQGKGDGEGDDSRVDNDGLTHLNIATGGFTLLVLSLICTL
3  EDEAEEIFLEVKLGGDAELWKSSQGKGDGEGDDSRVDNDGLTHLNIATGGFTLLVLSLICTL
4  EDEAEEIFLEVKLGGDAELWKSSQGKGDGEGDDSRVDNDGLTHLNIATGGFTLLVLSLICTL
5  EDEAEEIFLEAKLGGDAELWKSSQGKGDGEGDDSRVDNDGLTHLNIATGGFTLLVLSLICTL
6  EDEAEEIFLEVKLGGDAELWKSSQGK----GDNTVVDNDGLTHLNIATGGFTLLVLSLICTL
7  EDEAEEIFLEVKLGGDAELWKSSQGKGYSEGDDSRVDNDGLTHLNIATGGFTLLVLSLICTL
8  EDEAEEIFLEAKLGGDAELWKSSQGKGDGEGDDARVDNDGLTHLNIATGGFTLLVLSLICTL
```

T. b. brucei

```
1  KHTLENGGIPWYNRLSNATEQLQEMDMYAKEADSHLHELEEYEDEAEEIFLEVKLGGDVELWKSSQGK----GDDARVDNDGLTHLNIATGVAMLLIISLFCTL
2  KHTLENGGIPWYNRLSNATEQLQEMDMYAKEADSHIHELEEYEDEAEEIFLEVKLGGDAELLKSSQGK----GDDARLDNDGLTHLNIATGVAMLLIISLICTL
3  NYTLENGGIPWYNHLTNATEQLQEMDMYAKEADSHIHELEEYEDEAEEIFLEVKLGGDAELWKSSQGKGDGEGDNTVVDNDGLTHLNIATGVAMLLVLSLFCML
4  KHTLENGGIPWYNRLSNATEQLQEMDKYAKEADSHIHELEDYQHEAEEIFLEVKLGGDVELWKNSRGRDDVEGAGTDVNNDGLTHLNIATGVAMLLIIPLFCTL
5  KHTLENGGIPWYNRLSNATEQLQEMDMYAKEADSHIHELEEYEDEAEEIFLEVKLGGDAELWKSSQGK----GDDARVDNDGLTHLNIATGGFTLLVLSLICTL
6  KHTLENGGIPWYNHLTNATEQLQEMDMYAKEADSHIHELEEYEDEAEEIFLEVKLGGDAELWKSSQGK----GDDARVDNDGLTHLNIATGVAMLLIISLICTL
7  KHTLENGGIPWYNHLTNATEQLQEMDMYAKEADSHIHELEEYEDEAEEIFLEVKLGGDAELWKSSQGK----GDNTVVDNDGLTHLNIATGVAMLLIISLICTL
8  KHTLENGGIPWYNRLSNATEQLQEMDMYAKEADSHIHELEEYEDEAEEIFLEVKLGGDAELWKSSQGKGDGEGDDSRVDNDGLTHLNIATGVVMLLVLSLFCTL
9  SYTLENGGIPWYNRLSNATEQLQEMDKYAKEADSHLHELEEYEDEAEEIFLEVKLGGDAELWKSSQGK----GDNTVVDNDGLTHLNIATGGFTLLVLSLICTL
10 NYTLENGGIPWYNHLTNATAQLQEMDMYAKEADSHIHELEEYEDEAEEIFLEVKLGGDAELWKSSQGK----GDDARVDNDGLTHLNIATGVAMLLIISLICTL
11 NHTLENGGIPWYNHLTNATEQLQEMDMYAKEADSHIHELEEYEDEAEEIFLEAKLGGDAELWKSSQGKGYSGGDDTVVNNDGLTHLNIATGVAMLLIISLICTL
12 KHTLENGGIPWYNHLTNATEQLQEMDMYAKEADSHIHELEEYEDEAEEIFFEVKLGGDAELWKSSQGK----GDDARVDNDGLTHLNIATGVAMLLIISLICTL
13 KHTLENGGIPWYNRLSNATEQLQEMDMYAKEADSHIHELEEYEDEAEEIFLEVKLGGDAELWKSSQGK----GDNTVVDNDGLTHLNIATGVAMLLIISLICTL
14 NYTLENGGIPWYNHLTNATEQLQEMDMYAKEADSHIHELEEYEDEAEEIFLEAKLGGDAELWKSSQGKGYSGGDDTVVNNDGLTHLNVATGVAMLLIISLICTL
15 KHTLENGGIPWYNHLTNATEQLQEMDMYAKEADSHIHELEEYEDEAEEIFLEVKLGGDAELWKSSQGK----GDDARVDNDGLTHLNIATGVAMLLIISLICTL
16 SYTLENGGIPWYNRLSNATEQLQEMDKYAKEADSHLHELEEYEDEAEEIFLEVKLGGDAELWKSSQGKGDGEGDDSRVDNDGLTHLNIATGVAMLLIISLICTL
17 KHTLENGGIPWYNRLSNATEQLQEMDMYAKEADSHIHELEEYEDEAEEIFLEVKLGGDAELWKSSQGK----GDDARVDNDGLTHLNIATGVVMLLVLSLFCTL
```

T. equiperdum

```
1  LEEYEDEAEEIFLEAKLGGDAELWKSSQGKGDGEGDDARVDNDGLTHLNIATGGFTLLVLSLICTL
2  LEEYEDEAEEIFLEVKLGGDAELWKSSQGKGDGEGDDARVDNDGLTHLNIATGGFTLLVLSLICTL
3  LEEYEDEAEEIFLEVKLGGDAELWKSSQGK----GDNTVVDNDGLTHLNIATGGFTLLVLSLICTF
4  LEEYEDEAEEIFLEAKLGGDAELWKSSQGKGDGEGDDARVDNDGLTHLNIATGGFTLLVLSLICTL
```
