# Supplementary material for: Isolation and analysis of the genetic diversity of repertoires of VSG expression site containing telomeres from Trypanosoma brucei gambiense, T. b. brucei and T. equiperdum
Source: BMC Genomics. 2008 Aug 12;9:385. doi: 10.1186/1471-2164-9-385 (PMC2533676; doi:10.1186/1471-2164-9-385)
Supplement: Additional file 5 — Sup. Figure 5. Sequence alignments of ESAG5 sequences analysed in this manuscript. [file 1471-2164-9-385-S5.pdf]

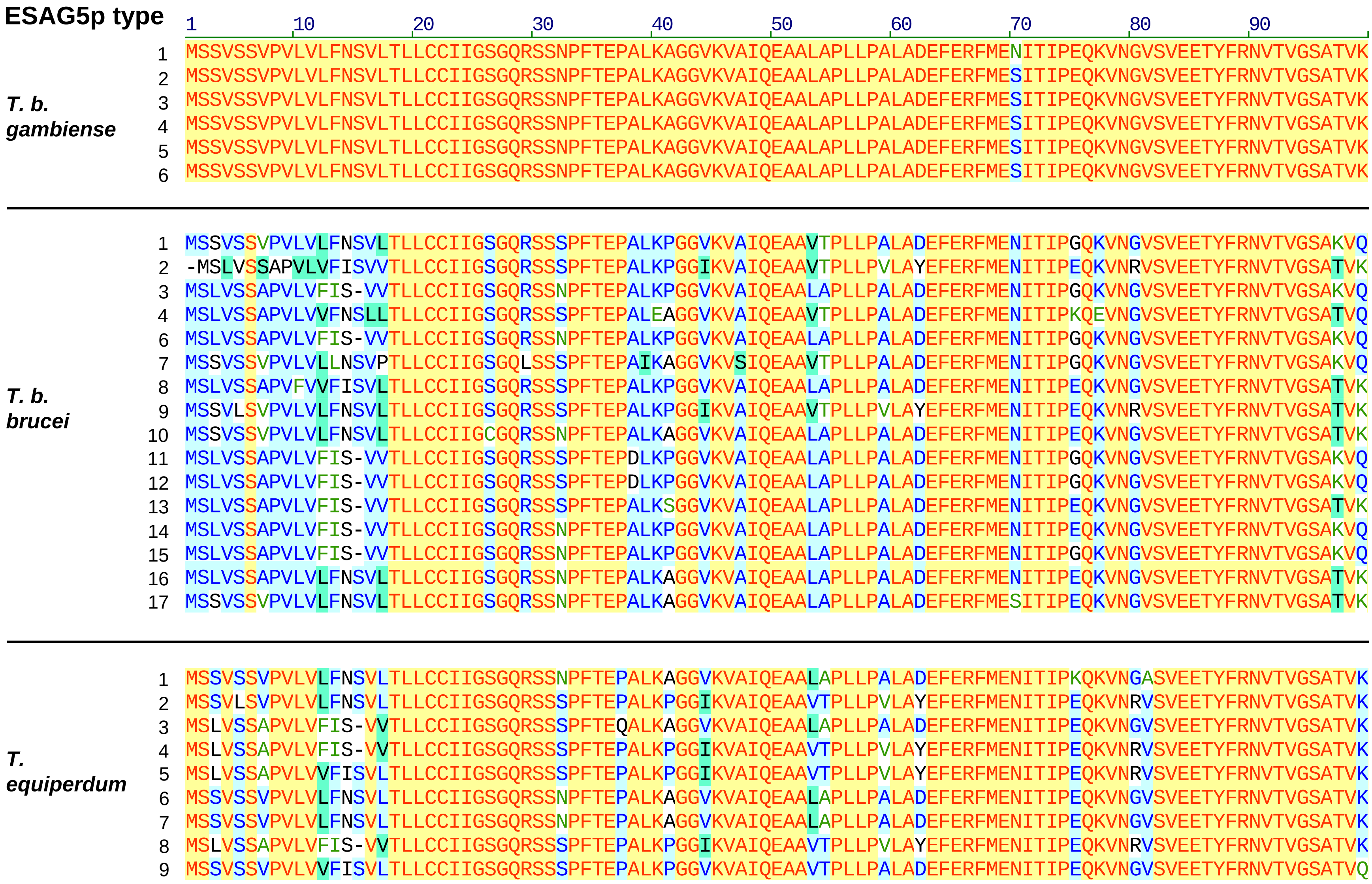
ESAG5p type
1 10 20 30 40 50 60 70 80 90
T. b. gambiense
1 MSSVSSVPVLVLFNSVLTLLCCIIGSGQRSSNPFTEPALKAGGVKVAIQEAALAPLLPALADEFERFMENITIPEQKVNGVSVEETYFRNVTVGSATVK
2 MSSVSSVPVLVLFNSVLTLLCCIIGSGQRSSNPFTEPALKAGGVKVAIQEAALAPLLPALADEFERFMESITIPEQKVNGVSVEETYFRNVTVGSATVK
3 MSSVSSVPVLVLFNSVLTLLCCIIGSGQRSSNPFTEPALKAGGVKVAIQEAALAPLLPALADEFERFMESITIPEQKVNGVSVEETYFRNVTVGSATVK
4 MSSVSSVPVLVLFNSVLTLLCCIIGSGQRSSNPFTEPALKAGGVKVAIQEAALAPLLPALADEFERFMESITIPEQKVNGVSVEETYFRNVTVGSATVK
5 MSSVSSVPVLVLFNSVLTLLCCIIGSGQRSSNPFTEPALKAGGVKVAIQEAALAPLLPALADEFERFMESITIPEQKVNGVSVEETYFRNVTVGSATVK
6 MSSVSSVPVLVLFNSVLTLLCCIIGSGQRSSNPFTEPALKAGGVKVAIQEAALAPLLPALADEFERFMESITIPEQKVNGVSVEETYFRNVTVGSATVK
T. b. brucei
1 MSSVSSVPVLVLFNSVLTLLCCIIGSGQRSSSPFTEPALKPGGVKVAIQEAAVTPLLPALADEFERFMENITIPGQKVNGVSVEETYFRNVTVGSAKVQ
2 -MSLVSSAPVLVFISVVTLLCCIIGSGQRSSSPFTEPALKPGGIKVAIQEAAVTPLLPVLAYEFERFMENITIPEQKVNRVSVEETYFRNVTVGSATVK
3 MSLVSSAPVLVFIS-VVTLLCCIIGSGQRSSNPFTEPALKPGGVKVAIQEAALAPLLPALADEFERFMENITIPGQKVNGVSVEETYFRNVTVGSAKVQ
4 MSLVSSAPVLVVFNSLLTLLCCIIGSGQRSSSPFTEPALEAGGVKVAIQEAAVTPLLPALADEFERFMENITIPKQEVNGVSVEETYFRNVTVGSATVQ
6 MSLVSSAPVLVFIS-VVTLLCCIIGSGQRSSNPFTEPALKPGGVKVAIQEAALAPLLPALADEFERFMENITIPGQKVNGVSVEETYFRNVTVGSAKVQ
7 MSSVSSVPVLVLLNSVPTLLCCIIGSGQLSSSPFTEPAIKAGGVKVSIQEAAVTPLLPALADEFERFMENITIPGQKVNGVSVEETYFRNVTVGSAKVQ
8 MSLVSSAPVFVVFISVLTLLCCIIGSGQRSSSPFTEPALKPGGVKVAIQEAALAPLLPALADEFERFMENITIPEQKVNGVSVEETYFRNVTVGSATVK
9 MSSVLSVPVLVLFNSVLTLLCCIIGSGQRSSSPFTEPALKPGGIKVAIQEAAVTPLLPVLAYEFERFMENITIPEQKVNRVSVEETYFRNVTVGSATVK
10 MSSVSSVPVLVLFNSVLTLLCCIIGCGQRSSNPFTEPALKAGGVKVAIQEAALAPLLPALADEFERFMENITIPEQKVNGVSVEETYFRNVTVGSATVK
11 MSLVSSAPVLVFIS-VVTLLCCIIGSGQRSSSPFTEPDLKPGGVKVAIQEAALAPLLPALADEFERFMENITIPGQKVNGVSVEETYFRNVTVGSAKVQ
12 MSLVSSAPVLVFIS-VVTLLCCIIGSGQRSSSPFTEPDLKPGGVKVAIQEAALAPLLPALADEFERFMENITIPGQKVNGVSVEETYFRNVTVGSAKVQ
13 MSLVSSAPVLVFIS-VVTLLCCIIGSGQRSSSPFTEPALKSGGVKVAIQEAALAPLLPALADEFERFMENITIPEQKVNGVSVEETYFRNVTVGSATVK
14 MSLVSSAPVLVFIS-VVTLLCCIIGSGQRSSNPFTEPALKPGGVKVAIQEAALAPLLPALADEFERFMENITIPEQKVNGVSVEETYFRNVTVGSAKVQ
15 MSLVSSAPVLVFIS-VVTLLCCIIGSGQRSSNPFTEPALKPGGVKVAIQEAALAPLLPALADEFERFMENITIPGQKVNGVSVEETYFRNVTVGSAKVQ
16 MSLVSSAPVLVLFNSVLTLLCCIIGSGQRSSNPFTEPALKAGGVKVAIQEAALAPLLPALADEFERFMENITIPEQKVNGVSVEETYFRNVTVGSATVK
17 MSSVSSVPVLVLFNSVLTLLCCIIGSGQRSSNPFTEPALKAGGVKVAIQEAALAPLLPALADEFERFMESITIPEQKVNGVSVEETYFRNVTVGSATVK
T. equiperdum
1 MSSVSSVPVLVLFNSVLTLLCCIIGSGQRSSNPFTEPALKAGGVKVAIQEAALAPLLPALADEFERFMENITIPKQKVNGASVEETYFRNVTVGSATVK
2 MSSVLSVPVLVLFNSVLTLLCCIIGSGQRSSSPFTEPALKPGGIKVAIQEAAVTPLLPVLAYEFERFMENITIPEQKVNRVSVEETYFRNVTVGSATVK
3 MSLVSSAPVLVFIS-VVTLLCCIIGSGQRSSSPFTEQALKAGGVKVAIQEAALAPLLPALADEFERFMENITIPEQKVNGVSVEETYFRNVTVGSATVK
4 MSLVSSAPVLVFIS-VVTLLCCIIGSGQRSSSPFTEPALKPGGIKVAIQEAAVTPLLPVLAYEFERFMENITIPEQKVNRVSVEETYFRNVTVGSATVK
5 MSLVSSAPVLVVFISVLTLLCCIIGSGQRSSSPFTEPALKPGGIKVAIQEAAVTPLLPVLAYEFERFMENITIPEQKVNRVSVEETYFRNVTVGSATVK
6 MSSVSSVPVLVLFNSVLTLLCCIIGSGQRSSNPFTEPALKAGGVKVAIQEAALAPLLPALADEFERFMENITIPEQKVNGVSVEETYFRNVTVGSATVK
7 MSSVSSVPVLVLFNSVLTLLCCIIGSGQRSSNPFTEPALKAGGVKVAIQEAALAPLLPALADEFERFMENITIPEQKVNGVSVEETYFRNVTVGSATVK
8 MSLVSSAPVLVFIS-VVTLLCCIIGSGQRSSSPFTEPALKPGGIKVAIQEAAVTPLLPVLAYEFERFMENITIPEQKVNRVSVEETYFRNVTVGSATVK
9 MSSVSSVPVLVVFISVLTLLCCIIGSGQRSSSPFTEPALKPGGVKVAIQEAAVTPLLPALADEFERFMENITIPEQKVNGVSVEETYFRNVTVGSATVQ

	ESAG5p type	100 110 120 130 140 150 160 170 180 190
T. b. gambiense	1	FWGSNKMVWNFCNVSATVPFTRFVYHSFWCYLYPCSGSAQAEIRNASVALWLDVSAGRGGLLDIHVGGSAIGNDDPLITLIGEGKSKVPKWLGGRVKDMY
	2	FWGLNKMVWNFCNVSATVPFTRFVYHSFWCYLYPCSGSAQAEIRNASVALWLDVSAGRGGLLDIHVGGSAIGNDDPLITLIGEGKSKVPKWLGGRVKDMY
	3	FWGSNKMVWNFCNVSATVPFTRFVYHSFWCYLYPCSGSAQAEIRNASVALWLDVSAGRGGLLDIHVGGSAIGNDDPLITLIGEGKSKVPKWLGGRVKDMY
	4	FWGSNKMVWNFCNVSANVPFTRFVYHSFWCYLYPCSGSAQAEIRNASVALWLDVSAGRGGLLDIHVGGSAIGNDDPLITLIGEGKSKVPKWLGGRVKDMY
	5	FWGSNKMVWNFCNVSATVPFTRFVYHSFWCYLYPCSGSAQAEIRNASVALWLDVSAGRGGLLDIHVGGSAIGNDDPLITLIGEGKSKVPKWLGGRVKDMY
	6	IWGSNKMVWNFCNVSATVPFTRFVYHSFWCYLYPCSGSAQAEIRNASVALWLDVSAGRGGLLDIHVGGSAIGNDDPLITLIGEGKSKVPKWLGGRVKDMY
T. b. brucei	1	FWGSNKMVWNFCNVSATVPFTRFVYHSFWCYLYPCSGSAQVEIRNASVALWLDVSATRGGLLDIHVGGSAIGNDDPLITLIGEGKSKVPKWLGGRVKDMY
	2	FEEPNKIILKFWNVSATVPFTRFVYHSFWCYLYPCSGSAQAEIRNGSVALWLDVSAGRGGLLDIHVGSSEIGMRDPLITLIGEGKSKMPKWLGGRVKDMY
	3	FWGSNKMVWNFCNVSATVPFTRFVYHSFWCYLYPCSGSAQAEIRNASVALWLDVSAGRGGILDIHVGGSGIGMGDPLITLIGEGKSKVPKWLGGRVKDMY
	4	FWESNKMVWNFCNVSATVPFTRFVYHSFWCYLYPCSGSAQAEIRNGSVELWLDVSAGRRGLLDIHVGGSAIGNDDPLITLIGEGKSKVPKWLGGRVKDMY
	6	FWGSNKMVWNFCNVSATVPFTRFVYHSFWCYLYPCSGSAQAEIRNASVALWLDVSAGRGGILDIHVGGSGIGMGDPLITLIGEGKSKVPKWLGGRVKDMY
	7	FWGSNKMVWNFCNVSATVPFTRFVYHSFWCYLYPCSGSAQAEIRNASVALWLDVSARRGGLLDIHVGGSGIGMGDPLITLIGEGKSKVPKWLGGRVKDMY
	8	FWGSNKMVWNFCNVSATVPFTRFVYHSFWCYLYPCSGSAQAEIRNASVALWLDVSAGRGGILGIHVGGSAIGNDDPLITLIGEGKSKVPKWLGGRVKDMY
	9	FEEPNKIILKFWNVSATVPFTRFVYHSFWCYLYPCSGSAQAEIRNGSVALWLDVSAGRGGLLDIHVGSSEIGMRDPLITLIGEGKSKMPKWLGGRVKDMY
	10	FWGSNKMVWNFCNVSATVPFTRFVYHSFWCYLYPCSGSAQAEIRNGSVELWLDVSAGRGGLLDIHVGGSAIGNDDPLITLIGEGKSKVPKWLGGRVKDMY
	11	FWGSNKMVWNFCNVSATVPFTRFVYHSFWCYMYPCSGSAQAEIRNASVALWLDVSARRGGLLDIHVGGSGIGMGDPLITLIGEGKSKVPKWLGGRVKDVY
	12	FWGSNKMVWNFCNVSATVPFTRFVYHSFWCYMYPCSGSAQAEIRNASVALWLDVSARRGGLLDIHVGGSGIGMGDPLITLIGEGKSKVPKWLGGRVKDVY
	13	FWGSNKMVWNFCNVSATVPFTRFVYHSFWCYLYPCSGSAQAEIRNASVALWLDVSAGRGGILDIHVGGSGIGMGDPLITLIGEGKSKVPKWLGGRVKDMY
	14	FWGSNKMVWNFCNVSATVPFTRFVYHSFWCYMYPCSGSAQAEIHNASVALWLDVSARRGGLLDIHVGGSGIGMGDPLITLIGEGKSKVPKWLGGRVKDVY
	15	FWGSNKMVWNFCNVSATVPFTRFVYHSFWCYLYPCSGSAQAEIRNASVALWLDVSATRGGLLDIHVGGSAIGNDDPLITLIGEGKSKVPKWLGGRVKDMY
	16	FWGSNKMVWNFCNVSATVPFTRFVYHSFWCYLYPCSGSAQAEIRNASVALWLDVSAGRGGLLDIHVGGSAIGNDDPLITLIGEGKSKVPKWLGGRVKDMY
	17	FWGSNKMVWNFCNVSATVPFTRFVYHSFWCYLYPCSGSAQAEIRNASVALWLDVSAGRGGLLDIHVGGSAIGNDDPLITLIGEGKSKVPKWLGGRVKDMY
T. equiperdum	1	FWGSNKMVWNFCNVSATVPFTRFVYHSFWCYLYPCSGSAQAEIRNASVALWLDVSAGRGGLLDIHVGGSAIGNDDPLITLIGEGKSKVPKWLGGRVKDMY
	2	FEEPNKIILKFWNVSATVPFTRFVYHSFWCYLYPCSGSAQAEIRNGSVALWLDVSAGRGGLLDIHVGSSEIGMRDPLITLIGEGKSKMPKWLGGRVKDMY
	3	FWGSNKMVWNFCNVSATVPFTRFVYHSFWCYLYPCSGSAQAEIRNASVALWLDVSAGRGGLLDIHVGGSAIGNDDPLITLIGEGKSKVPKWLGGRVKDMY
	4	FEEPNKIILKFWNVSATVPFTRFVYHSFWCYLYPCSGSAQAEIRNGSVALWLDVSAGRGGLLDIHVGSSEIGMRDPLITLIGEGKSKMPKWLGGRVKDMY
	5	FEEPNKIILKFWNVSATVPFTRFVYHSFWCYLYPCSGSAQAEIRNGSVALWLDVSAGRGGLLDIHVGSSEIGMRDPLITLIGEGKSKMPKWLGGRVKDMH
	6	FWGSNKMVWNFCNVSATVPFTRFVYHSFWCYLYPCSGSAQAEIRNASVALWLDVSAGRGGLLDIHVGGSAIGNDDPLITLIGEGKSKVPKWLGGRVKDMY
	7	FWGSNKMVWNFCNVSATVPFTRFVYHSFWCYLYPCSGSAQAEIRNASVALWLDVSAGRGGLLDIHVGGSAIGNDDPLITLIGEGKSKVPKWLGGRVKDMY
	8	FEEPNKIILKFWNVSATVPFTRFVYHSFWCYLYPCSGSAQVEIRNGSVALWLDVSAGRGGLLDIHVGSSEIGMRDPLITLIGEGKSKMPKWLGGRVKDMY
	9	FWGSNKMILKFWNVSATVPFTRFVYHSFWCYLYPCSGSAQAEIRNGSVALWLDVSAGRGGLLDITVGGSAIGMGDPLITLIGEGKSKMPKWLGGRVKDMY

ESAG5p type

```
             200       210       220       230       240       250       260       270       280       290
T. b. gambiense
 1  DKDVLPKLGHHIITAANQILAKKTKEIFHMFPIVFVNSSKIEYEQMRLELVVLADAADQLMLTEKVFSPRQPFPNWPVAVVSSFTALNNMLRLMIKSGHS
 2  DKDVLPKLGHHIITAANQILANKTKEIFHMFPIVFVNSSKIEYGQMRLELVVLADATDQLMLTEKVFSPRQPFPNWPVAVVSSFTALNNMLRLMIKSGHS
 3  DKDVLPKLGHHIITAANLILANKTKEIFHMFPIVFVNSSKIEYGQMRLELVVLADATDQLMLTEKVFSPRQPFPNWPVAVVSSFTALNNMLRLMIKSGHS
 4  DKDVLPKLGHHIITAANQILANKTKEIFHMFPIVFVNSSKIEYGQMRLELVVLADATDQLMLTEKVFSPRQPFPNWPVAVVSSFTALNNMLRLMIKSGHS
 5  DKDVLPKLGHHIITAANQILANKTKEIFHMFPIVFVNSSKIEYGQMRLELVVLADATDQLMLTEKVFSPRQPFPNWPVAVVSSFTALNNMLRLMIKSGHS
 6  DKDVLPKLGHHIITAANQILANKTKEIFHMFPIVFVNSSKIEYGQMRLELVVLADATDQLMLTEKVFSPRQPFPNWPVAVVSSFTALNNMLRLMIKSGHS

T. b. brucei
 1  DKDVLPKLGHHIITAANQILANKTKEIFHMFPIVFVNSSKIEYGQMRLELVVLPDAADQLILTEKVFSPRHPFPNFPVAVVSSFTALNNMLRLMIKRGHS
 2  DKDVLPKLGHHIITAVNQILANKAKEIFHMFPIVFVKSSKIEYGQMRLELVVLPDAADKLMLTENVFFPRQPFPNFPVAVVSSFTALNNMLRLMIKSGHL
 3  DKDVLPKLGHHIITAANQILANKTKEIFHMFPIVFVNSSKIEYGQMRLELVVLADAADQLMLTEKVFSPRQPFPNFPVAVVSSFTALNNMLRLMIKRGHS
 4  DKDVLPKLGHHIITAANQILANKTKEIFHMFPIVFVNSSKIEYGQMRLELVVLPDAADRLILTEKVFSPRHPFPNWSVAVVSSFTALNNMLRLMIKRGHS
 6  DKDVLPKLGHHIITAANQILANKTKEIFHMFPIVFVNSSKIEYGQMRLELVVLPDAADRLMLTEKVFSPRQPFPNFPVAVVSSFTALNNMLRLMIKRGHS
 7  DKDVLPKLGHHIITAANQILANKTKEIFHMFPIVFVNSSKIEYGQMRLELVVLADAADQLMLTEKVFSPRQPFPNFPVAVVSSFTALDNMLRLMIKRGHS
 8  DKDVLPKLGHHIITAVNQILANKTKEIFHMFPIVFVNSSKIEYGQMRLELVVLPDAADQLMLTEKVFSPRQPFPNFPVAVVSSFTALNNMLRLMIKRGHS
 9  DKDVLPKLGHHIITAVNQILANKAKEIFHMFPIVFVNSSKIEYGQMRLELVVLPDAADKLMLTENVFFPRQPFPNFPVAVVSSFTALNNMLRLMIKSGHL
10  DKDVLPKLGHHIITAANQILTNKTKEIFHMFPIVFVNSSKIEYGQMRLELVVFPDAADQLMLTEKVFSPLQPFPNWPVVVVSSFTALNNMLRLMIKKGHS
11  DKDVLPKLGHHIITAVNQILANKTKEIFHMFPIVFVNSSKIEYGQMRLEMVVLPDAADQLMLTEKVFSPRQPFPNFPVAVVSSFTALNNMLRLMIKRGHS
12  DKDVLPKLGHHIITAVNQILANKTKEIFHMFPIVFVNSSKIEYGQMRLEMVVLADAADQLMLTEKVFSPRQPFPNFPVAVVSSFTALNNMLRLMIKRGHS
13  DKDVLPKLGHHIITAANQILANKTKEIFHMFPIVFVNSSKIEYGQMRLELVVLPDAADQLMLTEKVFSPRHPFPNFPVAVVSSFTALNNMLRLMIKRGHS
14  DKDVLPKLGHHIITAVNQILANKTKEIFHMFPIVFVNSSKIEYGQMRLEMVVLPDAADQLMLTEKVFSPRQPFPNFPVAVVSSFTALNNMLRLMIKRGHS
15  DKDVLPKLGHHIITAANQILANKTKEIFHMFPIVFFNSSKIEYGQMRLELVVLPDAADQLMLTEKVFSPRHPFPNFPVAVVSSFTALNNMLRLMIKRGYS
16  DKDVLPKLGHHIITAANQILANKTKEIFHMFPIVFVNSSKIEYGQMRLELVVLADAADQLMLTEKVFPPRQPFPNWPVAVVSSFTALNNMLRLMIKRGHS
17  DKDVLPKLGHHIITGVNRILTNKTKEIFHMFPIVFVNSSKIEYGQMRLELVVLADAADQLMLTEKVFSPRQPFPNWPVAVVSSFTALNNMLRLMIKSGHS

T. equiperdum
 1  DKDVLPKLGHHIITGVNRILTNKTKEIFHMFPIVFVNSSKIEYGQMRLELVVLADAADQLMLTEKVFSPRQPFPNWPVAVVSSFTALNNMLRLMIKSGHS
 2  DKDVLPKLGHHIITAVNQILANKAKEIFHMFPIVFVNSSKIEYGQMRLELVVLPDAADKLMLTENVFFPRQPFPNFPVAVVSSFTALNNMLRLMIKSGHL
 3  DKDVLPKLGHHIITAVNEILANKAKEIFHMFPIVFVNSSKIEYGQMRLELVVLPDAADQLMLTENVFFPRQPFPNFPVAVVSSFTALNNMLRLMIKSGHS
 4  DKDVLPKLAHHIITAVNQILANKAKEIFHMFPIVFVNSSKIEYGQMRLELVVLPDAADKLMLTENVFFPRQPFPNFPVAVVSSFTALNNMLRLMIKSGHL
 5  DKDVLPKLAHHIITAVNQILANKAKEIFHMFPIVFVNSSKIEYGQMRLELVVLPDAADKLMLTENVFFPRQPFPNFPVAVVSSFTALNNMLRLMIKSGHL
 6  DKDVLPKLGHHIITAANQILANKTKEIFHMFPIVFVNSSKIEYGQMRLELVVLADAADQLMLTKKVFSPRQPFPNWPVAVVSSFTALNNMLRLMIKSGHS
 7  DKDVLPKLGHHIITAANQILANKTKEIFHMFPIVFVNSSKIEYGQMRLELVVLPDAADKLMLTENVFFPRQPFPNWPVAVVSSFTALNNMLRLMIKSGHS
 8  DKDVLPKLAHHIITGVNRILTNKTKEIFHMFPIVFVNSSKIEYGQMRLELVVLPDAADKLMLTENVFFPRQPFPNFPVAVVSSFTALNNMLRLMIKSGHL
 9  DKDVLPKLGHHIITGVNRILTNKTKEIFHMFPIVFVSSSKIEYGQMRLELVVLPDAADKLMLTEKVFFPRQPFPNWPVAVVSSFTALNNMLRLMIKRGHW
```

ESAG5p type

Species		300 310 320 330 340 350 360 370 380 390
T. b. gambiense	1	MVHVPFPLRYVFSSKTAQRQLDRLCFGCGSEATFELKTAPWLNSLNEKMVTVKFQDVEVTVDLLPRVGAPISLFSMLMNVSVQAAHIALIDGAAHANLDS
	2	MVHVPFPLRYVFSSKTAQRQLDRLCFGCGSEATFELKTAPWLNSLNEKMVTVKFQDVEVTVDLLPRGGAPISLFSMLMNVSVQAAHIALIDGVAHANLDS
	3	MVHVPFPLRYVFSSKTAQRQLDRLCFGCGSEATFELKTAPWLNSLNEKMVTVKFQDVEVTVDLLPRGGAPISLFSMLMNVSVQAAHIALIDGVAHANLDS
	4	MVHVPFPLRYVFSSKTAQRQLDRLCFGCGSEATFELKTAPWLNSLNEKMVTVKFQDVEVTVDLLPRGGAPISLFSMLMNVSVQAAHIALIDGVAHANLDS
	5	MVHVPFPLRYVFSSKTAQRQLDRLCFGCGSEATFELKTAPWLNSLNEKMVTVKFQDVEVTVDLLPRGGAPISLFSMLMNVSVQAAHIALIDGVAHANLDS
	6	MVHVPFPLRYVFSSKTAQRQLDRLCFGCGSEATFELKTAPWLNSLNEKMVTVKFQDVEVTVDLLPRGGAPISLFSMLMNVSVQAAHIALIDGVAHANLDS
T. b. brucei	1	VVRVPFPLRYVFSSNAAQRQLDSLCFGCASEATFELKAAPWLKYLNEKLVTVKFQDVEVTVDLLPRGGDPISLFSMLMNVSVEAVHIALIDGVAHANLDS
	2	VVRVPFPLRYVISSNAAQRQLDRLCSGCASEATFELKTAPWLKSLNKKLFTFNYRDVEVAVDLLPRGGAPISLFSMLMNVSAQAAHIALVDGATHANLDS
	3	VVRVPFPLRYVFSSNAAQRQLDSLCFGCASEATFELKAAPWLKSLNEKLVTVKFQDVEVTVDLLPRGGDPISLFSMLMNVSVETVHIALIDGVAHANLDS
	4	MVHVPFPLRYVFSSKTAQRQLDSLCFGCASEATFELKTAPWLNSLNEKLVTVKFQDVEVTVDLLPRGGAPISLFSMLMNVSVEAVHIALIDGVAHANLDS
	6	VVRVPFPLRYVFSSNAAQRQLDSLCFGCASEATFELKTAPWLKSLNEKMVTVKFQDVEVTVDLFPRGGDPISLFSMLMNVSVETVHIALIDGVAHANLDS
	7	VVRVPFPLRYVFSSNAAQRQLDSLFFGCASEATFELKTAPWLKSLNEKLVTVKFQDVEVTVDLLPRGGDPISLFSMLMNVSVETVHIALIDGVAHANLDS
	8	VVRVPFPLRYVFSSNAAQRQLDSLCFGCASEATFELKTAPWLNSVNEKMVTVKFQDVEVTVDLLPRGGAPISLFSMLMNVSVQAAHIALIDGVAHANLDS
	9	VVRVPFPLRYVISSNAAQRQLDRLCSGCASEATFELKTAPWLKSLNKKLFTFNYRDVEVAVDLLPRGGAPISLFSMLMNVSAQAAHIALVDGATHANLDS
	10	MVRVPFPLRYVFSSNAAQRQLDSLCPGCASEATFELKTAPWLNSLNEKLVTVMFQDVDFYVSMLPRGGDPISFFSMLMNVSVQAAHIALIDGVAHANLDS
	11	VVRVPFPLRYVFSSNAAQRQLDSLCFGCASEATFELKTAPWLKSLNEKLVTVKFQDVEVTVDLLPRGGDPISLFSMLMNVSVETVHIALIDGVAHANLDS
	12	VVRVPFPLRYVFSSNAAQRQLDSLCFGCASEATFELKTAPWLKSLNEKLVTVKFQDVEVTVDLLPRGGDPISLFSMLMNVSVETVHIALIDGVAHANLDS
	13	VVRVPFPLRYVFSSKTAQRQLDRLCFGCGSEATFELKTAPWLKSLNEKLVTVKFQDVEVTVDLLPRGGDPISLFSMLMNVSVETVHIALIDGVAHANLDS
	14	VVRVPFPLRYVFSSNAAQRQLDSLCFGCASEATFELKTAPWLKSLNEKLVTVKFQDVEVTVDLLPRGGDPISLFSMLMNVSVETVHIALIDGVAHANLDS
	15	VVRVPFPLRYVFSSNAAQRQLDSLCFGCASAATFELKTAPWLNSLNEKMVTVKFQDVEVTVDLLPRGGDPISLFSMLMNVSVETVHIALIDGVAHANLDS
	16	VVRVPFPLRYVFSSNAAQRQLDSLCFGCASEATFELKAAPWLKSLNEKMVTVKFQDIEVTVDLLPRGGDPISLFSMLMNVSVQAAHIALIDGVAHANLDS
	17	MVHVPFPLRYVFSSKTAQRQLDRLCFGCGSEATFELKTAPWLNSLNEKMVTVKFQDVEVTVDLLPRGGAPISLFSMLMNVSVQAAHIALIDGVAHANLDS
T. equiperdum	1	MVHVPFPLRYVFSSKTAQRQLDRLCFGCGSEATFELKTAPWLNSLNEKMVTVKFQDVEVTVDLLPRGGAPISLFSMLMNVSVQAARIALIDGVAHANLDS
	2	VVRVPFPLRYVISSNAAQRQLDRLCSGCASEATFELKTAPWLKSLNKKLFTFNYRDVEVAVDLLPRGGAPISLFSMLMNVSAQAAHIALVDGATHANLDS
	3	MVHVPFPLRYVFSSNAAQRQLDRLCSGCASEATFELKTAPWLKSLNEKMVTVKFQDVEVAVDLLPRGGAPISLFSMLMNVSAQAAHIALVDGVAHANLDS
	4	VVRVPFPLRYVISSNAAQRQLDRLCSGCASEATFELKTAPWLKSLNKKLFTFNYRDVEVAVDLLPRGGAPISLFSMLMNVSVQAAHIALVDGATHANLDS
	5	VVRVPFPLRYVISSNAAQRQLDRLCSGCASEATFELKTAPWLKSLNKKLFTFNYRDVEVAVDLLPRGGAPISLFSMLMNVSVQAAHIALVDGATHANLDS
	6	MVHVPFPLRYVFSSKTAQRQLDRLCFGCGSEATFELKTAPWLNSLNEKMVTVKFQDVEVTVDLLPRGGAPISLFSMLMNVSVQAAHIALIDGVAHANLDS
	7	MVHVPFPLRYVFSSKTAQRQLDRLCFGCGSEATFELKTAPWLNSLNEKMVTVKFQDVEVTVDLLPRGGAPISLFSMLMNVSVQAAHIALIDGVAHANLDS
	8	VVRVPFPLRYVISSNAAQRQLDRLCSGCASEATFELKTAPWLKSLNKKLFTFNYRDVEVAVDLLPRGGAPISLFSMLMNVSVQAAHIALVDGATHANLDS
	9	VVRVPFPLRYVFYSKATQRQLDSLCFGCASEATFELKTAPWLKSLNEKLFTFNYRDVEVAVDLLPRGGDPISLFSMLMNVSVEAVHIALIDGVAHANLDS

ESAG5p type

		400 410 420 430 440 450 460 470 480
T. b. gambiense	1	VDTNVSVTSSRIDGLDSSIMNTKIRDLINGMVLPLLNFKRYAFPAPFDLSGIHLNITTEGGIAGVDLVRALGSLSILPHLR
	2	VDTNVSVTSSRIDGLNSSTMNTKIRDLINGMVLPLLNFKRYAFPAPFDLSGIHLNITTEGGIAGVDLVRALGSLSILPHLR
	3	VDTNVSVTSSRIDGLNSSTMNTKIRDLINGMVLPLLNFKRYAFPAPFDLSGIHLNITTEGGIAGVDLVRALGSLSILPHLR
	4	VDTNVSVTSSRIDGLNSSTMNTKIRDLINGMVLPLLNFKRYAFPAPFDLSGIHLNITTEGGIAGVDLVRALGSLSILPHLR
	5	VDTNVSVTSSRIDGLNSSTMNTKIRDLINGMVLPLLNFKRYAFPAPFDLSGIHLNITTEGGIAGVDLVHALGSLSILPHLR
	6	VDTNVSVTSSRIDGLNSSTMNTKIRDLINGMVLPLLNFKRYAFPAPFDLSGIHLNITTEGGIAGVDLVRALGSLSILPHLR
T. b. brucei	1	VDTNVSVTSSRIDGLDSSTMNTKIRDLINGMVLPLLNFKRYAFPAPFDLSGMHLNITTEGGIAGVDLVRALGSLSILPHLR
	2	VDTNVSVTSSRIDGLDSSTMNTKIRDLINLSWINLN--VTYTFPAPFDLCTKHVNITSECYVAGFNLVRALGSLSILPHLR
	3	VDTNVSVTSSRIDGLDSSTMNTKIRDLINGMVLPLLNFKRYAFPAPFDLSGIHLNITTEGGIAGVDLVRALGSLSILPHLR
	4	VDTNVSVTSSRIDGLDSSTMNTKIRDLINGMVLPLLNFKRYAFPAPFDLSGMHLNITTEGGIAGVDLVRALGSLSILPHLR
	6	VDTNVSVTSSRIDGLDSSTMNTKIRDLINGMVLPLLNFKRYAFPAPFDLSGIHLNIITEGGIAGVDLVRALGSLSILPHLR
	7	VDTNVSVTSSRIDGLDSSTMNTKIRDLINGMVLPLLNFKRYAFPAPFDLSGIHLNITTEGGIAGVDLVRALGSLSILPHLR
	8	VDTNVSVTSSRIDGLYSSTMNTKIRDLINGMVLPLLNFKRYAFPAPFDLSGIHLNITTEGGIAGVDLVRALGSLSILPHLR
	9	VDTNVSVTSSRIDGLDSSTMNTKIRDLINLSWINLN--VTYTFPAPFDLCTKHVNITSECYVAGFNLVRALGSLSILPHLR
	10	VDAAMSVTSSRIDGLDSSIMNTKIRDLINGMVLPLLNFKRYAFPAPFDLSGIHLNITTEGGIAGVDLVRALRSLSILPHLR
	11	VDTNVSVTSSRIDGLDSSTMNTKIRDLINGMVLPLLNFKRYAFPAPFDLSGMHLNITTEGGIAGVDLVRALGSLSILPHLR
	12	VDTNVSVTSSRIDGLDSSTMNTKIRDLINGMVLPLLNFKRYAFPAPFDLSGMHLNITTEGGIAGVDLVRALGSLSILPHLR
	13	VDTNVSVTSSRIDGLDSSTMNTKIRDLINGMVLPLLNFKRYAFPAPFDLSGIHLNITTEGGIAGVDLVRALGSLGILPHLR
	14	VDTNVSVTSSRIDGLDSSTMNTKIRDLINGMVLPLLNFKRYAFPAPFDLSGMHLNITTEGGIAGVDLVRALGSLSILPHLR
	15	VDTNVSVTSSRIDGLDSSTMNTKIRDLINGMVLPLLNFKRYAFPAPFDLSGIHLNITTEGGIAGVDLVRALGSLSILPHLR
	16	VDTNVSVTSSRIDGLDSSTMNTKIRDLINGMVLPLLNFKRYAFPAPFDLSGIHLNITTEGGIAGVDLVRALGSLSILPHLR
	17	VDTNVSVTSSRIDGLDSSTMNTKIRDLINGMVLPLLNFKRYAFPAPFDLSGIHLNITTEGGIAGVDLVRALGSLSILPHLR
T. equiperdum	1	VDTNVSVTSSRIDGLDSSIMNTNIRDLINGMVLPLLNFKRYAFPAPFDLSGIHLNITSEGGIAGVDLVRALGSLSILPHLR
	2	VDTNVSVTSSRIDGLDSSTMNTKIRDLINLSWINLN--VTYTFPAPFDLCTKHVNITSECYVAGFNLVRALGSLSILPHLR
	3	VDTNVSVTSSRIDGLDSSTMNTKIRDLINGMVLPLLNFKRYAFPAPFDLSGMHLNITTEGGIAGVDLVRALGSLSILPYLR
	4	VDTNVSVTSSRIDGLDSSTMNTKIRDLINLSWINLN--VTYTFPVPFDLCTKHVNITSECYVAGFNLVRALGSLSILPHLR
	5	VDTNVSVTSSRIDGLDSSTMNTKIRDLINLSWINLN--VTYTFPVPFDLCTKHVNITSECYVAGFNLVRALGSLSILPHLR
	6	VDTNVSVTSSRIDGLDSSTMNTKIRDLINGMVLPLLNFKRYAFPAPFDLSGIHLNITSECGIAGVDLVRALGSLSILPHLR
	7	VDTNVSVTSSRIDGLDSSTMNTKIRDLINGMVLPLLNFKRYAFPAPFDLSGIHLNITSECGIAGVDLVRALGSLSILPHLR
	8	VDTNVSVTSSRIDGLDSSTMNTKIRDLINLSWINLN--VTYTFPVPFDLCTKHVNITSECYVAGFNLVRALGSLSILPHLR
	9	VDTNVSVTSSRIDGLDSSTMNTKIRDLINLSGINLN--VTYTFPAPFDLSGIHLNITSECYVAGFNLVRALGSLSILPHLR
