# Supplementary material for: Isolation and analysis of the genetic diversity of repertoires of VSG expression site containing telomeres from Trypanosoma brucei gambiense, T. b. brucei and T. equiperdum
Source: BMC Genomics. 2008 Aug 12;9:385. doi: 10.1186/1471-2164-9-385 (PMC2533676; doi:10.1186/1471-2164-9-385)
Supplement: Additional file 6 — Supplementary Table 2. Non-synonymous substitution rates for different genes located within trypanosome BESs isolated from Trypanosoma brucei gambiense DAL 972, T. b. brucei EATRO 2340, or T. equiperdum STIB 818. [file 1471-2164-9-385-S6.pdf]

Supplementary Table 2

Non-synonymous substitution rates within BESs

Locus	Subspecies	Parameters (M1a)	Parameters (M2a)	2Δ ln L	p-value
ESAG6	T.b.g.	ω0=0.000, p0=0.560	ω0=0.157, p0=0.752	3.460	0.177
		ω1=1.000, p1=0.440	ω1=1.000, p1=0.216		
			ω2=7.215, p2=0.032		
	T.b.b.	ω0=0.031, p0=0.708	ω0=0.043, p0=0.724	61.58	4.25×10^{-14}
		ω1=1.000, p1=0.292	ω1=1.000, p1=0.220		
			ω2=4.132, p2=0.056		
	T. eq.	ω0=0.000, p0=0.536	ω0=0.000, p0=0.711	6.276	0.043
		ω1=1.000, p1=0.464	ω1=1.000, p1=0.022		
			ω2=2.207, p2=0.266		
ESAG5	T.b.g.	ω0=0.691, p0=1.000	ω0=0.557, p0=0.686	-0.010	1.000
		ω1=1.000, p1=0.000	ω1=1.000, p1=0.314		
			ω2=1.010, p2=0.000		
	T.b.b.	ω0=0.000, p0=0.484	ω0=0.262, p0=0.852	71.95	2.22×10^{-16}
		ω1=1.000, p1=0.516	ω1=1.000, p1=0.000		
			ω2=4.056, p2=0.148		
	T. eq.	ω0=0.000, p0=0.441	ω0=0.000, p0=0.544	14.70	6.42×10^{-4}
		ω1=1.000, p1=0.559	ω1=1.000, p1=0.317		
			ω2=3.186, p2=0.140		
ESAG2	T.b.g.	ω0=0.021, p0=0.714	ω0=0.032, p0=0.735	10.63	0.005
		ω1=1.000, p1=0.286	ω1=1.000, p1=0.236		
			ω2=5.335, p2=0.029		
	T.b.b.	ω0=0.058, p0=0.691	ω0=0.060, p0=0.689	40.72	0.000
		ω1=1.000, p1=0.309	ω1=1.000, p1=0.289		
			ω2=5.220, p2=0.021		
	T. eq.	ω0=0.163, p0=0.848	ω0=0.217, p0=0.970	7.521	0.023
		ω1=1.000, p1=0.152	ω1=1.000, p1=0.000		
			ω2=6.592, p2=0.030		

Sup. Table 2. Non-synonymous substitution rates for different genes located within trypanosome BESs. Sequences from different BES loci from *Trypanosoma brucei gambiense* DAL 972 (T.b.g.), *T. b. brucei* EATRO 2340 (T.b.b.), or *T. equiperdum* STIB 818 (T. eq) were analysed. The dN/ dS ratio (ω) and weights (p) of discrete priors for the relative rate of non-synonymous (dN) to synonymous (dS) substitutions under models M1a (2 atoms) and M2a (3 atoms); p-value of the difference in log-likelihoods between M2a and M1a under a χ^2-distribution with two degrees of freedom. Rows with a p value of < 0.05 indicate significant support for adaptive evolution.
