# Supplementary material for: Isolation and analysis of the genetic diversity of repertoires of VSG expression site containing telomeres from Trypanosoma brucei gambiense, T. b. brucei and T. equiperdum
Source: BMC Genomics. 2008 Aug 12;9:385. doi: 10.1186/1471-2164-9-385 (PMC2533676; doi:10.1186/1471-2164-9-385)
Supplement: Additional file 7 — Supplementary Table 3. Table with the ratio of nonsynonymous-to-synonymous substitutions (ω) along ESAG6, ESAG5 and ESAG2. [file 1471-2164-9-385-S7.pdf]

Supplementary Table 3

Ratio of nonsynonymous-to-synonymous substitutions

A) ESAG6

codon	AA	Tbg	Tbr	Teq	region
**1**	M	0.358	0.074	0.262	**SP**
**2**	R	0.390	0.651	0.344	**SP**
**3**	F	0.399	0.129	0.383	**SP**
**4**	W	**2.403**	**4.105**	**2.182**	**SP**
**5**	F	0.399	0.779	0.383	**SP**
**6**	V	0.397	0.681	0.350	**SP**
**7**	L	0.429	0.173	0.431	**SP**
**8**	L	0.429	0.160	0.431	**SP**
**9**	A	0.450	0.711	0.454	**SP**
**10**	L	**2.505**	0.924	0.439	**SP**
**11**	L	0.429	0.160	0.431	**SP**
**12**	G	0.392	0.623	0.339	**SP**
**13**	K	0.377	0.088	0.302	**SP**
**14**	E	0.373	1.839	0.293	**SP**
**15**	I	0.391	1.375	0.335	**SP**
**16**	Y	0.421	**4.075**	**2.147**	**SP**
**17**	A	0.393	0.096	0.329	**SP**
**18**	-	0.568	0.198	**2.147**	
**19**	-	0.568	0.174	0.419	
**20**	Y	0.421	0.148	0.419	
**21**	E	0.373	0.546	0.293	
**22**	N	0.371	0.092	0.306	
**23**	E	0.373	1.181	**2.135**	
**24**	R	0.390	0.104	0.344	
**25**	N	0.371	0.092	0.306	
**26**	A	0.386	0.096	0.325	
**27**	L	0.431	0.159	0.431	
**28**	N	0.404	0.122	0.378	
**29**	A	0.386	0.095	0.325	
**30**	T	0.428	0.164	0.397	
**31**	A	0.450	0.159	0.461	
**32**	A	0.403	0.121	0.417	
**33**	N	0.371	0.092	0.386	
**34**	K	0.377	0.088	0.383	
**35**	V	0.412	0.109	0.422	
**36**	C	0.420	0.148	0.478	
**37**	G	**2.248**	1.619	**2.152**	
**38**	L	0.442	0.175	0.498	
**39**	S	0.440	0.160	0.491	

**40**	T	0.456	0.168	0.511
**41**	Y	0.421	0.148	0.475
**42**	L	0.430	0.157	0.490
**43**	K	0.377	0.088	0.383
**44**	G	0.392	0.099	0.413
**45**	I	0.367	0.077	0.362
**46**	A	0.397	0.107	0.424
**47**	H	0.445	0.172	0.504
**48**	R	0.388	0.099	0.409
**49**	V	0.387	0.095	**2.147**
**50**	N	0.404	0.745	0.444
**51**	S	0.421	0.834	0.473
**52**	E	0.380	0.092	0.395
**53**	S	0.384	0.105	0.416
**54**	A	0.403	0.142	0.443
**55**	V	0.397	0.106	0.422
**56**	V	0.403	0.116	**2.151**
**57**	T	0.402	0.112	0.432
**58**	E	0.373	0.086	0.374
**59**	K	0.377	0.088	0.383
**60**	L	0.442	0.175	0.498
**61**	S	0.442	0.157	0.489
**62**	D	0.366	0.088	0.376
**63**	L	0.429	0.160	0.484
**64**	K	0.377	0.089	0.383
**65**	M	1.556	0.476	0.349
**66**	R	0.388	0.621	0.409
**67**	S	0.421	0.180	0.473
**68**	I	0.431	0.185	0.484
**69**	Q	0.433	0.208	0.488
**70**	L	0.429	0.200	0.484
**71**	Q	0.451	0.201	0.488
**72**	L	0.514	0.221	0.498
**73**	S	0.415	0.197	0.489
**74**	V	0.359	1.252	**2.179**
**75**	M	0.331	0.660	0.349
**76**	R	0.427	0.223	0.521
**77**	N	0.375	0.159	0.444
**78**	R	0.359	0.132	0.409
**79**	V	0.369	0.140	0.422
**80**	P	0.414	0.210	0.499
**81**	S	0.403	0.197	0.487
**82**	G	0.422	0.204	0.506
**83**	E	0.352	0.125	0.394
**84**	K	1.807	**3.959**	0.402
**85**	D	0.338	0.118	0.376
**86**	C	0.391	0.188	0.478
**87**	K	0.349	0.119	0.383
**88**	D	0.366	0.151	0.434

**89**	I	0.413	0.183	0.460
**90**	R	0.361	0.813	0.417
**91**	T	0.362	0.133	0.410
**92**	L	0.452	0.255	0.537
**93**	L	0.401	0.200	0.484
**94**	K	0.349	0.119	0.383
**95**	T	0.362	0.133	0.410
**96**	V	0.359	0.127	0.399
**97**	L	0.401	0.200	0.484
**98**	R	0.361	0.138	0.417
**99**	N	0.344	0.124	0.386
**100**	E	0.352	0.125	0.394
**101**	F	0.371	0.166	0.448
**102**	T	0.362	0.133	0.410
**103**	F	0.407	0.207	0.496
**104**	Q	0.405	0.208	0.488
**105**	Q	0.405	0.208	0.488
**106**	E	0.352	0.125	0.394
**107**	L	0.401	0.200	0.484
**108**	E	0.352	0.125	**2.147**
**109**	E	0.345	0.115	0.374
**110**	M	0.331	0.101	**2.143**
**111**	R	0.361	0.138	0.417
**112**	N	0.375	0.159	0.444
**113**	A	0.358	**1.995**	0.401
**114**	S	0.452	0.256	0.535
**115**	A	0.369	0.140	0.424
**116**	L	0.404	0.199	0.484
**117**	A	0.358	0.127	0.401
**118**	A	0.358	0.127	0.401
**119**	A	0.374	0.152	0.443
**120**	A	0.358	0.127	0.401
**121**	A	0.374	0.152	0.443
**122**	G	0.367	0.139	0.424
**123**	L	0.401	**4.132**	**2.175**
**124**	A	0.358	0.127	0.401
**125**	A	0.374	0.168	0.447
**126**	G	0.363	0.131	0.413
**127**	R	0.359	0.132	0.409
**128**	L	0.427	0.233	0.511
**129**	E	0.345	0.115	0.374
**130**	E	0.376	0.124	0.395
**131**	W	0.411	0.071	0.499
**132**	I	0.363	0.046	0.424
**133**	F	0.371	0.050	0.448
**134**	V	0.359	0.045	0.399
**135**	F	0.371	0.050	0.448
**136**	A	0.358	0.045	0.401
**137**	Q	0.423	0.064	0.485

**138**	A	0.362	0.045	0.414	
**139**	A	1.819	0.084	**2.181**	
**140**	D	0.422	1.063	**2.140**	
**141**	R	1.573	1.007	**2.164**	
**142**	S	0.415	0.336	0.310	
**143**	S	0.415	0.060	0.310	
**144**	Q	0.405	0.366	0.304	
**145**	F	0.371	0.050	0.233	
**146**	C	0.436	0.077	0.381	
**147**	I	0.340	0.043	0.125	
**148**	S	0.399	0.053	0.269	
**149**	V	**4.967**	1.056	**2.198**	**HV**
**150**	G	0.367	0.046	0.198	**HV**
**151**	K	0.357	1.003	**2.166**	**HV**
**152**	T	**5.664**	**3.531**	**2.126**	**HV**
**153**	I	**6.761**	**4.030**	**2.185**	**HV**
**154**	P	1.920	0.882	0.268	**HV**
**155**	P	0.374	0.111	**2.122**	**HV**
**156**	E	0.376	0.099	0.142	**HV**
**157**	Q	**2.305**	1.636	**2.146**	**HV**
**158**	N	**5.477**	**3.709**	**2.120**	**HV**
**159**	N	0.375	0.750	0.227	**HV**
**160**	L	0.401	0.158	0.299	
**161**	Q	0.395	0.153	0.278	
**162**	E	0.345	0.084	0.142	
**163**	C	0.391	0.147	0.285	
**164**	F	0.371	0.127	0.233	
**165**	D	0.338	0.086	0.168	
**166**	G	0.363	0.097	0.185	
**167**	T	1.648	1.412	**2.116**	
**168**	I	0.363	0.106	0.198	
**169**	G	0.363	0.097	0.185	
**170**	P	0.414	0.167	0.326	
**171**	E	0.345	0.084	0.142	
**172**	T	0.429	0.169	0.354	
**173**	L	0.403	0.155	0.309	
**174**	Y	0.443	0.198	0.390	
**175**	K	0.349	0.087	0.149	
**176**	I	0.363	0.106	0.198	
**177**	E	0.352	0.092	0.163	
**178**	D	0.338	0.086	0.138	
**179**	S	0.415	0.155	0.310	
**180**	R	0.415	0.170	0.338	
**181**	V	0.359	0.093	0.170	
**182**	K	0.349	0.087	0.149	
**183**	E	0.352	0.092	0.163	
**184**	S	0.414	0.158	0.313	
**185**	A	0.369	0.095	0.192	
**186**	K	**1.955**	**3.213**	**2.119**	

**187**	K	1.648	1.286	**2.165**	
**188**	S	1.712	0.805	**2.164**	
**189**	L	0.401	0.158	0.299	
**190**	Q	**2.138**	0.900	**2.176**	
**191**	L	0.452	0.209	0.412	
**192**	H	0.377	0.130	0.243	
**193**	E	0.345	0.084	0.142	
**194**	A	0.369	**2.477**	0.199	
**195**	L	0.404	0.170	0.315	
**196**	S	0.415	0.155	0.310	
**197**	S	0.452	0.209	0.407	
**198**	I	0.363	0.106	**2.119**	
**199**	S	0.452	0.209	0.569	
**200**	F	0.407	0.164	0.547	
**201**	N	0.372	**4.110**	**2.157**	
**202**	S	0.393	0.137	0.533	
**203**	L	0.401	0.158	0.540	
**204**	G	0.372	0.127	0.515	
**205**	A	1.648	1.529	0.504	
**206**	E	1.345	1.192	0.472	
**207**	S	0.344	0.582	0.480	
**208**	I	0.394	0.139	0.539	
**209**	R	0.447	0.203	0.565	**Box 1**
**210**	G	0.363	0.097	0.497	**Box 1**
**211**	G	0.363	0.097	0.497	**Box 1**
**212**	N	0.344	0.091	0.480	**Box 1**
**213**	G	0.367	0.103	0.503	**Box 1**
**214**	K	1.544	**3.307**	0.490	**Box 1**
**215**	D	0.338	0.554	0.473	**Box 1**
**216**	G	0.363	0.613	0.497	
**217**	C	0.435	0.153	0.536	
**218**	N	0.351	0.091	0.480	
**219**	L	0.401	0.158	0.540	
**220**	V	0.369	0.104	0.502	
**221**	R	0.359	0.098	0.494	
**222**	T	0.375	0.099	0.495	
**223**	D	0.366	0.112	0.510	**Box 2**
**224**	T	1.772	0.718	0.480	**Box 2**
**225**	D	0.375	1.233	0.516	**Box 2**
**226**	G	0.372	0.114	0.515	**Box 2**
**227**	I	0.340	0.988	0.464	**Box 2**
**228**	L	0.404	0.160	0.540	**Box 2**
**229**	N	1.377	**3.300**	0.490	**Box 2**
**230**	G	0.367	0.103	0.503	**Box 2**
**231**	G	0.389	0.106	0.503	**Box 2**
**232**	S	0.415	0.155	0.543	**Box 2**
**233**	P	0.402	1.512	0.541	**Box 2**
**234**	T	0.362	0.647	0.495	**Box 2**
**235**	R	0.427	0.174	0.555	**Box 2**

**236**	H	0.419	0.170	0.551	**Box 2**
**237**	N	0.375	0.120	0.516	**Box 2**
**238**	L	0.404	0.208	0.540	**Box 2**
**239**	T	0.374	0.154	0.508	
**240**	W	0.411	0.230	0.549	
**241**	G	0.372	0.158	0.515	
**242**	G	0.372	0.158	0.515	
**243**	G	0.363	0.138	0.497	
**244**	V	0.371	0.147	0.502	
**245**	M	0.331	0.107	0.456	
**246**	N	0.344	0.131	0.480	
**247**	F	0.371	0.173	0.519	
**248**	G	0.363	0.138	0.497	
**249**	S	0.452	0.265	0.569	
**250**	Y	0.393	0.195	0.535	
**251**	Q	0.395	0.203	0.534	
**252**	N	0.375	0.166	0.516	
**253**	G	0.363	0.138	0.497	
**254**	S	0.356	0.146	0.499	
**255**	M	0.331	0.107	0.456	
**256**	Y	0.443	0.253	0.565	
**257**	V	0.375	0.159	0.514	
**258**	E	0.352	0.132	0.485	
**259**	G	0.372	0.156	0.507	
**260**	G	0.372	0.153	0.502	
**261**	E	0.345	0.122	0.472	
**262**	Y	0.443	0.253	0.565	
**263**	G	0.372	0.158	0.515	
**264**	D	0.338	0.125	0.473	
**265**	A	0.374	0.160	0.515	
**266**	T	0.362	0.146	0.512	
**267**	E	0.352	1.499	**2.184**	
**268**	Y	0.443	1.437	**2.159**	
**269**	G	0.372	0.158	0.515	
**270**	A	0.358	0.801	**2.155**	
**271**	V	0.359	0.134	0.488	
**272**	R	0.427	0.226	0.555	
**273**	W	0.411	0.230	0.549	
**274**	T	0.431	0.184	0.545	
**275**	E	0.345	1.266	0.472	
**276**	D	0.338	0.125	0.473	
**277**	P	0.414	0.254	0.548	
**278**	S	0.356	0.848	**2.155**	
**279**	K	0.349	0.126	0.477	
**280**	V	0.369	0.146	0.502	
**281**	S	0.393	0.188	0.533	
**282**	I	0.340	0.112	0.464	
**283**	F	0.371	0.173	0.519	
**284**	K	1.417	**2.281**	0.490	

**285**	D	0.338	0.125	0.473
**286**	V	0.423	1.282	0.552
**287**	I	0.363	0.149	0.503
**288**	R	0.463	0.281	0.575
**289**	L	0.427	0.243	0.555
**290**	F	1.640	0.173	0.519
**291**	A	0.369	0.148	0.504
**292**	R	0.454	0.277	**2.161**
**293**	F	0.407	0.215	0.547
**294**	Q	1.854	**2.188**	**2.158**
**295**	E	0.345	0.122	0.472
**296**	A	0.358	0.134	0.490
**297**	K	0.349	0.126	0.477
**298**	N	0.344	0.131	0.480
**299**	A	0.358	0.808	0.490
**300**	V	0.369	0.147	0.502
**301**	M	1.204	0.107	0.456
**302**	K	1.778	**4.122**	0.508
**303**	K	1.659	1.326	0.477
**304**	I	0.340	0.112	0.464
**305**	K	0.349	0.126	0.477
**306**	T	0.380	0.166	0.519
**307**	T	1.703	0.166	0.519
**308**	V	0.369	0.147	0.502
**309**	D	0.338	0.125	0.473
**310**	E	0.345	0.122	0.472
**311**	L	0.401	0.209	0.540
**312**	T	0.374	0.903	0.508
**313**	K	0.349	0.126	0.477
**314**	C	0.429	0.222	0.544
**315**	I	0.340	1.159	0.464
**316**	G	0.367	0.146	0.503
**317**	Q	0.405	**2.361**	0.542
**318**	K	0.357	0.131	0.492
**319**	E	0.352	0.132	0.485
**320**	A	0.374	0.940	0.515
**321**	E	0.345	0.129	0.485
**322**	L	0.452	0.264	0.570
**323**	T	0.380	0.165	0.519
**324**	N	0.344	0.804	0.480
**325**	D	0.338	0.771	0.473
**326**	Q	0.405	0.217	0.542
**327**	L	1.937	1.479	**2.159**
**328**	Y	0.443	0.202	0.565
**329**	E	0.352	0.095	0.485
**330**	E	0.345	0.087	0.472
**331**	F	0.371	0.130	0.519
**332**	I	0.404	0.140	0.491
**333**	W	0.411	0.180	0.549

**334**	E	0.352	0.095	0.485
**335**	T	0.431	0.173	0.555
**336**	I	0.340	0.078	0.464
**337**	N	**5.774**	1.785	0.516
**338**	R	0.360	0.100	**2.155**
**339**	L	0.401	0.161	0.540
**340**	E	0.352	0.095	0.485
**341**	L	0.427	0.191	0.555
**342**	S	0.415	0.158	0.543
**343**	K	0.357	0.098	0.490
**344**	R	0.359	0.100	0.494
**345**	V	0.369	0.654	0.502
**346**	S	0.356	0.106	0.499
**347**	E	0.345	0.087	0.472
**348**	Q	0.395	0.156	0.534
**349**	S	0.463	**3.327**	0.573
**350**	A	0.403	**2.568**	0.542
**351**	F	0.404	1.858	0.540
**352**	G	0.372	0.113	0.515
**353**	E	0.345	0.087	0.472
**354**	E	0.345	0.600	0.472
**355**	E	0.352	0.095	0.485
**356**	E	0.376	0.095	0.485
**357**	T	0.375	0.113	0.508
**358**	I	0.404	0.148	0.539
**359**	V	**2.102**	**1.972**	0.555
**360**	K	0.349	0.089	0.477
**361**	F	0.403	1.664	0.542
**362**	N	0.375	0.124	0.516
**363**	Y	0.393	0.149	0.535
**364**	T	0.380	0.124	0.519
**365**	A	0.358	0.097	0.490
**366**	E	0.345	0.089	0.472
**367**	P	**2.109**	0.168	0.548
**368**	V	0.424	0.166	0.550
**369**	R	0.415	0.176	0.552
**370**	G	0.363	0.100	0.497
**371**	P	0.414	0.982	0.548
**372**	F	0.407	0.167	0.547
**373**	T	0.374	0.113	0.508
**374**	V	0.363	1.156	0.497
**375**	A	0.369	0.108	0.504
**376**	G	0.367	0.106	0.503
**377**	A	**2.508**	**3.978**	0.553
**378**	N	0.375	0.742	0.516
**379**	A	0.362	1.541	0.495
**380**	A	0.359	**2.171**	0.488
**381**	A	0.358	0.097	0.490
**382**	I	0.359	1.344	0.488

**383**	H	1.822	1.412	0.542
**384**	L	**2.031**	1.715	0.544
**385**	S	0.356	0.106	0.499
**386**	V	0.375	0.740	0.514
**387**	S	0.403	0.923	0.542
**388**	T	0.431	0.173	0.555
**389**	A	0.374	0.118	0.515
**390**	A	0.358	0.097	0.490
**391**	L	0.403	0.159	0.543
**392**	C	0.391	0.150	0.536
**393**	R	0.391	**2.711**	0.536
**394**	S	0.415	1.677	0.543
**395**	A	0.375	**3.128**	**2.158**
**396**	L	0.404	0.161	0.540
**397**	L	0.401	0.161	0.540
**398**	L	0.401	0.937	**2.157**
**399**	G	0.363	0.100	0.497
**400**	V	0.369	0.108	0.502
**401**	L	0.427	1.030	0.555

B) ESAG5

codon	AA	Tbg	Tbr	Teq	region
**1**	M	0.693	0.263	0.202	**SP**
**2**	S	0.695	0.339	0.483	**SP**
**3**	S	0.695	**4.051**	**2.499**	**SP**
**4**	V	0.695	0.284	0.373	**SP**
**5**	S	0.695	0.771	1.783	**SP**
**6**	S	0.695	0.288	0.384	**SP**
**7**	V	0.695	**4.046**	**2.354**	**SP**
**8**	P	0.695	0.297	0.420	**SP**
**9**	V	0.694	0.266	0.256	**SP**
**10**	L	0.695	0.615	0.389	**SP**
**11**	V	0.694	0.273	0.310	**SP**
**12**	L	0.695	**3.519**	**2.603**	**SP**
**13**	F	0.695	0.522	0.354	**SP**
**14**	N	0.694	**4.002**	**2.141**	**SP**
**15**	S	0.695	0.315	0.443	**SP**
**16**	V	0.694	0.333	0.256	**SP**
**17**	L	0.695	**4.052**	**2.924**	**SP**
**18**	T	0.695	0.290	0.391	**SP**
**19**	L	0.694	0.269	0.296	
**20**	L	0.695	0.309	0.433	
**21**	C	0.695	0.280	0.358	
**22**	C	0.695	0.280	0.358	

**23**	I	0.694	0.274	0.328
**24**	I	0.694	0.274	0.328
**25**	G	0.695	0.300	0.417
**26**	S	0.695	0.445	0.327
**27**	G	0.695	0.281	0.357
**28**	Q	0.695	0.314	0.453
**29**	R	0.695	1.070	0.453
**30**	S	0.695	0.274	0.327
**31**	S	0.694	0.268	0.282
**32**	S	0.694	**4.010**	1.515
**33**	P	0.695	0.313	0.440
**34**	F	0.694	0.265	0.258
**35**	T	0.695	0.276	0.322
**36**	E	0.695	0.277	0.352
**37**	P	0.695	0.289	1.774
**38**	A	0.695	1.399	0.559
**39**	L	0.695	0.788	0.579
**40**	K	0.695	0.530	0.546
**41**	P	0.695	**4.051**	**2.091**
**42**	G	0.695	0.382	0.541
**43**	G	0.695	0.428	0.582
**44**	V	0.694	0.940	1.875
**45**	K	0.694	0.353	0.510
**46**	V	0.694	0.317	0.460
**47**	A	0.694	1.032	0.466
**48**	I	0.694	0.303	0.435
**49**	Q	0.695	0.454	0.605
**50**	E	0.694	0.343	0.501
**51**	A	0.694	0.357	0.513
**52**	A	0.694	0.320	0.466
**53**	V	0.694	**3.484**	**1.951**
**54**	T	0.694	**3.598**	**1.996**
**55**	P	0.695	0.449	0.598
**56**	L	0.695	0.466	0.607
**57**	L	0.695	0.447	0.600
**58**	P	0.695	0.449	0.598
**59**	A	0.695	1.651	**2.039**
**60**	L	0.694	0.338	0.494
**61**	A	0.694	0.357	0.513
**62**	D	0.694	1.076	**1.968**
**63**	E	0.694	0.343	0.501
**64**	F	0.695	0.376	0.538
**65**	E	0.695	0.369	0.537
**66**	R	0.695	0.372	0.529
**67**	F	0.694	0.315	0.460
**68**	M	0.693	0.292	0.406
**69**	E	0.694	0.343	0.501
**70**	N	0.755	0.961	0.461
**71**	I	0.694	0.360	0.519

**72**	T	0.695	0.409	0.563
**73**	I	0.694	0.303	0.435
**74**	P	0.695	0.397	0.542
**75**	G	0.694	**4.025**	**1.952**
**76**	Q	0.695	0.454	0.605
**77**	K	0.694	1.499	0.510
**78**	V	0.694	0.317	0.460
**79**	N	0.695	0.377	0.539
**80**	G	0.694	1.348	**2.009**
**81**	V	0.694	0.352	**1.966**
**82**	S	0.694	0.330	0.482
**83**	V	0.695	0.390	0.553
**84**	E	0.695	0.369	0.537
**85**	E	0.694	0.343	0.501
**86**	T	0.694	0.326	0.473
**87**	Y	0.695	0.373	0.534
**88**	F	0.694	0.315	0.460
**89**	R	0.695	0.390	0.549
**90**	N	0.695	0.377	0.539
**91**	V	0.694	0.352	0.506
**92**	T	0.695	0.375	0.519
**93**	V	0.695	0.364	0.551
**94**	G	0.695	0.438	0.592
**95**	S	0.695	0.402	0.560
**96**	A	0.694	0.347	0.513
**97**	K	0.695	1.704	0.565
**98**	V	0.694	0.317	0.460
**99**	Q	0.695	**3.727**	**2.085**
**100**	F	0.754	0.331	0.498
**101**	W	0.695	**3.834**	**2.701**
**102**	G	0.695	1.790	**2.065**
**103**	S	0.756	**2.142**	**2.117**
**104**	N	0.695	0.338	0.460
**105**	K	0.695	0.382	0.512
**106**	M	0.693	0.748	1.758
**107**	V	0.694	0.940	1.780
**108**	W	0.695	**2.029**	**2.040**
**109**	N	0.695	1.467	1.921
**110**	F	0.694	0.315	0.418
**111**	C	0.695	1.617	**2.024**
**112**	N	0.695	0.377	0.504
**113**	V	0.694	0.352	0.466
**114**	S	0.695	0.359	0.528
**115**	A	0.694	0.320	0.423
**116**	T	0.755	0.326	0.431
**117**	V	0.694	0.347	0.448
**118**	P	0.695	0.404	0.525
**119**	F	0.694	0.315	0.418
**120**	T	0.695	0.369	0.503

**121**	R	0.695	0.420	0.578
**122**	F	0.694	0.289	0.418
**123**	V	0.695	0.346	0.518
**124**	Y	0.695	0.422	0.584
**125**	H	0.695	0.376	0.547
**126**	S	0.695	0.382	0.546
**127**	F	0.694	0.289	0.418
**128**	W	0.695	0.418	0.582
**129**	C	0.695	0.341	0.508
**130**	Y	0.695	0.422	0.584
**131**	L	0.695	1.548	0.564
**132**	Y	0.695	0.334	0.499
**133**	P	0.695	0.321	0.479
**134**	C	0.695	0.425	0.586
**135**	S	0.694	0.300	0.442
**136**	G	0.695	0.341	0.506
**137**	S	0.695	0.375	0.542
**138**	A	0.695	0.353	0.526
**139**	Q	0.695	0.375	0.547
**140**	V	0.694	1.056	1.906
**141**	E	0.695	0.330	0.502
**142**	I	0.694	0.300	0.439
**143**	R	0.695	1.913	0.598
**144**	N	0.695	0.337	0.504
**145**	A	0.695	**3.598**	**2.057**
**146**	S	0.694	0.300	0.442
**147**	V	0.694	0.315	0.466
**148**	A	0.695	**3.561**	0.524
**149**	L	0.695	0.397	0.564
**150**	W	0.695	0.418	0.582
**151**	L	0.695	0.388	0.555
**152**	D	0.695	0.328	0.495
**153**	V	0.694	0.315	0.466
**154**	S	0.695	0.407	0.571
**155**	A	0.695	0.353	0.526
**156**	T	0.695	**4.056**	0.565
**157**	R	0.695	0.333	0.493
**158**	G	0.695	1.287	0.506
**159**	G	0.695	0.391	0.565
**160**	L	0.695	**4.041**	0.603
**161**	L	0.695	0.441	0.603
**162**	D	0.695	1.087	0.492
**163**	I	0.694	0.300	0.458
**164**	H	0.694	0.312	**2.586**
**165**	V	0.694	0.297	0.475
**166**	G	0.695	0.341	0.506
**167**	G	0.694	0.858	1.907
**168**	S	0.695	0.382	0.546
**169**	A	0.695	**4.054**	**2.012**

**170**	I	0.694	0.281	0.388
**171**	G	0.695	0.348	0.553
**172**	N	0.694	**4.056**	**2.396**
**173**	D	0.694	**4.056**	**2.512**
**174**	D	0.695	0.328	0.495
**175**	P	0.695	0.401	0.571
**176**	L	0.695	0.397	0.564
**177**	I	0.694	0.300	0.439
**178**	T	0.694	0.297	0.431
**179**	L	0.695	0.371	0.530
**180**	I	0.694	0.322	0.482
**181**	G	0.694	0.307	0.458
**182**	E	0.695	0.330	0.502
**183**	G	0.695	0.341	0.506
**184**	K	0.695	0.341	0.512
**185**	S	0.695	0.439	0.578
**186**	K	0.694	0.317	0.472
**187**	V	0.694	0.897	1.775
**188**	P	0.695	0.321	0.479
**189**	K	0.694	0.317	0.472
**190**	W	0.695	0.418	0.582
**191**	L	0.694	0.305	0.453
**192**	G	0.695	0.382	0.553
**193**	G	0.695	0.341	0.506
**194**	R	0.695	0.372	0.544
**195**	V	0.694	0.290	0.415
**196**	K	0.695	0.341	0.512
**197**	D	0.694	0.286	0.410
**198**	M	0.693	0.470	0.360
**199**	Y	0.695	0.305	1.944
**200**	D	0.694	0.273	0.410
**201**	K	0.694	0.293	0.472
**202**	D	0.694	0.273	0.410
**203**	V	0.694	0.292	0.466
**204**	L	0.694	0.292	0.453
**205**	P	0.695	0.363	0.569
**206**	K	0.694	0.293	0.472
**207**	L	0.694	0.285	0.453
**208**	G	0.695	0.311	**1.956**
**209**	H	0.694	0.290	0.230
**210**	H	0.694	0.290	0.230
**211**	I	0.694	0.281	0.201
**212**	I	0.694	0.270	0.156
**213**	T	0.695	0.320	0.279
**214**	A	0.695	1.040	**2.802**
**215**	A	0.694	**4.049**	1.936
**216**	N	0.694	0.275	0.182
**217**	Q	0.756	1.460	**3.074**
**218**	I	0.694	0.270	0.156

**219**	L	0.695	0.359	0.366
**220**	A	0.695	**3.289**	**2.708**
**221**	N	0.755	0.308	0.271
**222**	K	0.695	0.310	0.281
**223**	T	0.695	1.064	**2.714**
**224**	K	0.694	0.293	0.234
**225**	E	0.694	0.287	0.224
**226**	I	0.694	0.281	0.201
**227**	F	0.695	0.305	0.266
**228**	H	0.694	0.290	0.230
**229**	M	0.693	0.267	0.132
**230**	F	0.694	0.276	0.181
**231**	P	0.695	0.360	0.362
**232**	I	0.694	0.270	0.156
**233**	V	0.695	0.316	0.288
**234**	F	0.694	0.275	0.181
**235**	V	0.695	0.978	0.288
**236**	N	0.695	0.864	**2.003**
**237**	S	0.694	0.282	0.476
**238**	S	0.695	0.297	0.513
**239**	K	0.695	0.310	0.541
**240**	I	0.694	0.274	0.423
**241**	E	0.695	0.302	0.532
**242**	Y	0.695	0.305	0.529
**243**	G	0.755	0.351	0.589
**244**	Q	0.695	0.365	0.602
**245**	M	0.693	0.267	0.396
**246**	R	0.695	0.335	0.571
**247**	L	0.695	0.321	0.518
**248**	E	0.695	0.302	0.532
**249**	L	0.695	1.210	0.589
**250**	V	0.695	0.316	0.544
**251**	V	0.694	0.291	0.497
**252**	L	0.695	0.649	0.506
**253**	P	0.695	**4.056**	**2.090**
**254**	D	0.694	0.273	0.446
**255**	A	0.695	0.322	0.553
**256**	A	0.755	0.320	0.554
**257**	D	0.695	0.300	0.526
**258**	Q	0.695	**4.021**	**2.639**
**259**	L	0.695	0.359	0.597
**260**	I	0.693	1.246	0.396
**261**	L	0.694	0.285	0.486
**262**	T	0.695	0.300	0.513
**263**	E	0.694	0.287	1.938
**264**	K	0.694	0.661	**2.505**
**265**	V	0.694	0.275	0.448
**266**	F	0.694	0.275	0.453
**267**	S	0.695	**3.519**	**2.020**

**268**	P	0.695	0.326	0.554
**269**	R	0.695	1.531	0.602
**270**	H	0.695	**4.052**	0.573
**271**	P	0.695	0.306	0.511
**272**	F	0.694	0.281	0.453
**273**	P	0.695	0.378	0.595
**274**	N	0.695	0.320	0.534
**275**	F	0.695	**4.051**	**3.116**
**276**	P	0.695	0.870	0.511
**277**	V	0.694	0.283	0.448
**278**	A	0.694	0.578	0.457
**279**	V	0.694	0.283	0.448
**280**	V	0.694	0.283	0.448
**281**	S	0.695	0.311	0.513
**282**	S	0.695	0.360	0.572
**283**	F	0.694	0.281	0.453
**284**	T	0.695	0.312	0.513
**285**	A	0.694	0.284	0.457
**286**	L	0.695	0.340	0.560
**287**	N	0.694	0.548	0.454
**288**	N	0.694	0.282	0.454
**289**	M	0.693	0.271	0.396
**290**	L	0.694	0.294	0.486
**291**	R	0.695	0.330	0.545
**292**	L	0.695	0.340	0.560
**293**	M	0.693	0.271	0.396
**294**	I	0.694	0.276	0.423
**295**	K	0.695	0.323	0.541
**296**	R	0.694	**3.978**	1.913
**297**	G	0.695	0.360	0.439
**298**	H	0.695	1.331	0.431
**299**	S	0.695	1.361	**2.462**
**300**	V	0.693	**3.926**	1.522
**301**	V	0.694	0.303	0.336
**302**	R	0.694	**3.374**	1.708
**303**	V	0.694	0.320	0.366
**304**	P	0.695	0.381	0.461
**305**	F	0.694	0.281	0.281
**306**	P	0.695	0.375	0.414
**307**	L	0.695	0.374	0.455
**308**	R	0.695	0.353	0.402
**309**	Y	0.695	0.317	0.371
**310**	V	0.694	0.283	0.281
**311**	F	0.695	1.048	1.792
**312**	S	0.695	0.383	1.918
**313**	S	0.695	0.338	0.406
**314**	N	0.694	**3.986**	**2.320**
**315**	A	0.695	**4.009**	1.787
**316**	A	0.694	0.284	1.573

**317**	Q	0.695	0.354	0.431
**318**	R	0.695	0.330	0.392
**319**	Q	0.695	0.354	0.431
**320**	L	0.695	0.350	0.412
**321**	D	0.695	0.312	0.367
**322**	S	0.695	**3.971**	1.766
**323**	L	0.694	0.294	0.320
**324**	C	0.695	1.655	0.483
**325**	F	0.695	**3.944**	**2.297**
**326**	G	0.695	0.369	0.455
**327**	C	0.695	0.323	0.381
**328**	A	0.695	**3.534**	1.839
**329**	S	0.695	0.308	0.351
**330**	E	0.694	0.766	0.329
**331**	A	0.694	0.264	0.369
**332**	T	0.695	0.279	0.414
**333**	F	0.695	0.271	0.377
**334**	E	0.695	0.270	0.375
**335**	L	0.695	0.316	0.509
**336**	K	0.695	0.273	0.387
**337**	A	0.695	**3.879**	0.414
**338**	A	0.695	0.277	0.404
**339**	P	0.695	0.278	0.404
**340**	W	0.695	0.302	0.478
**341**	L	0.695	0.278	0.412
**342**	K	0.694	**3.985**	1.626
**343**	Y	0.695	0.773	0.464
**344**	L	0.695	0.860	0.502
**345**	N	0.694	0.264	0.283
**346**	E	0.694	0.325	1.749
**347**	K	0.695	0.273	0.387
**348**	L	0.693	**4.030**	1.645
**349**	V	0.694	0.300	1.597
**350**	T	0.695	0.279	0.414
**351**	V	0.694	0.300	1.597
**352**	K	0.694	1.235	1.691
**353**	F	0.694	0.293	1.653
**354**	Q	0.695	0.627	1.882
**355**	D	0.694	0.263	0.274
**356**	V	0.694	0.368	0.372
**357**	E	0.695	0.379	0.375
**358**	V	0.694	0.300	0.281
**359**	T	0.695	**3.921**	1.787
**360**	V	0.694	0.268	0.444
**361**	D	0.695	1.931	0.476
**362**	L	0.695	0.605	0.549
**363**	L	0.695	0.563	0.556
**364**	P	0.695	0.294	0.556
**365**	R	0.695	0.270	0.475

**366**	G	0.755	0.268	0.450
**367**	G	0.694	0.266	0.437
**368**	D	0.695	**1.977**	**1.974**
**369**	P	0.695	0.293	0.555
**370**	I	0.694	0.327	0.365
**371**	S	0.695	0.492	0.557
**372**	L	0.695	1.800	0.515
**373**	F	0.694	0.341	0.396
**374**	S	0.694	0.359	0.421
**375**	M	0.693	0.311	0.337
**376**	L	0.695	0.485	0.559
**377**	M	0.693	0.311	0.337
**378**	N	0.695	0.413	0.481
**379**	V	0.694	0.386	0.444
**380**	S	0.695	0.404	0.462
**381**	V	0.695	1.860	**2.554**
**382**	E	0.695	**3.758**	**1.988**
**383**	A	0.694	**3.659**	0.454
**384**	V	0.694	**3.653**	1.871
**385**	H	0.694	0.378	1.867
**386**	I	0.694	0.394	0.462
**387**	A	0.694	0.348	0.401
**388**	L	0.695	0.441	0.481
**389**	I	0.694	1.095	**2.347**
**390**	D	0.695	0.400	0.476
**391**	G	0.694	0.370	0.437
**392**	V	0.755	1.860	**1.952**
**393**	A	0.695	1.906	**1.962**
**394**	H	0.694	0.378	0.448
**395**	A	0.694	0.391	0.454
**396**	N	0.694	0.343	0.397
**397**	L	0.695	0.441	0.515
**398**	D	0.694	0.335	0.388
**399**	S	0.695	0.393	0.462
**400**	V	0.694	0.345	0.391
**401**	D	0.694	0.335	0.388
**402**	T	0.694	1.328	0.410
**403**	N	0.694	**3.864**	0.397
**404**	V	0.694	1.467	0.444
**405**	S	0.695	0.434	0.452
**406**	V	0.695	0.430	0.498
**407**	T	0.695	0.443	0.516
**408**	S	0.695	0.490	0.549
**409**	S	0.695	0.528	0.586
**410**	R	0.695	0.426	0.499
**411**	I	0.694	0.394	0.462
**412**	D	0.694	0.335	0.388
**413**	G	0.695	0.466	0.537
**414**	L	0.695	0.483	0.549

**415**	D	0.755	1.698	0.476
**416**	S	0.695	0.528	0.586
**417**	S	0.695	0.434	0.452
**418**	T	0.755	1.852	**1.958**
**419**	M	0.693	0.311	0.337
**420**	N	0.695	0.413	0.486
**421**	T	0.695	0.443	0.516
**422**	K	0.695	0.417	1.934
**423**	I	0.694	0.327	0.249
**424**	R	0.695	0.426	0.386
**425**	D	0.694	0.335	0.269
**426**	L	0.695	0.441	0.407
**427**	I	0.694	0.394	0.347
**428**	N	0.695	0.413	0.372
**429**	G	-	-	-
**430**	M	-	-	-
**431**	V	-	-	-
**432**	L	-	-	-
**433**	P	-	-	-
**434**	L	-	-	-
**435**	L	-	-	-
**436**	N	-	-	-
**437**	F	-	-	-
**438**	K	-	-	-
**439**	R	-	-	-
**440**	Y	0.695	0.408	0.366
**441**	A	0.695	1.888	1.787
**442**	F	0.694	0.341	0.276
**443**	P	0.695	0.466	0.427
**444**	A	0.694	0.324	1.537
**445**	P	0.695	0.427	0.456
**446**	F	0.695	0.360	0.372
**447**	D	0.695	0.354	0.362
**448**	L	0.695	0.385	0.407
**449**	S	0.695	1.549	1.731
**450**	G	0.695	**3.694**	**2.402**
**451**	M	0.694	**4.055**	**2.612**
**452**	H	0.695	0.397	0.426
**453**	L	0.695	1.768	1.845
**454**	N	0.694	0.314	0.278
**455**	I	0.694	0.313	0.249
**456**	T	0.695	1.572	0.409
**457**	T	0.694	1.282	1.616
**458**	E	0.695	0.356	0.370
**459**	G	0.695	1.943	**2.530**
**460**	G	0.694	**3.543**	**2.298**
**461**	I	0.694	1.195	1.673
**462**	A	0.694	0.353	0.339
**463**	G	0.695	0.416	0.450

**464**	V	0.694	1.205	1.562
**465**	D	0.695	1.325	1.710
**466**	L	0.694	0.336	0.315
**467**	V	0.695	0.386	0.369
**468**	R	0.756	0.492	0.521
**469**	A	0.694	0.324	0.283
**470**	L	0.694	0.336	0.315
**471**	G	0.695	1.686	0.398
**472**	S	0.695	0.465	0.498
**473**	L	0.695	0.408	0.429
**474**	S	0.694	1.003	0.301
**475**	I	0.694	0.329	0.299
**476**	L	0.695	0.408	0.429
**477**	P	0.695	0.346	0.343
**478**	H	0.695	0.397	1.838
**479**	L	0.695	0.408	0.429
**480**	R	0.696	0.531	0.564

C) ESAG2

**codon**	**AA**	**Tbg**	**Tbr**	**Teq**	**region**
**1**	M	0.183	0.062	0.218	**SP**
**2**	R	0.198	0.063	0.218	**SP**
**3**	C	0.199	0.999	0.220	**SP**
**4**	E	1.094	0.133	0.254	**SP**
**5**	I	0.204	0.972	0.219	**SP**
**6**	V	0.223	0.070	0.221	**SP**
**7**	I	**2.304**	1.002	**2.783**	**SP**
**8**	A	**2.237**	1.158	0.904	**SP**
**9**	V	1.130	0.096	0.220	**SP**
**10**	G	0.207	0.066	0.224	**SP**
**11**	V	**3.100**	1.200	0.767	**SP**
**12**	F	0.214	0.068	0.219	**SP**
**13**	V	**3.489**	1.091	0.276	**SP**
**14**	A	0.185	0.062	0.219	**SP**
**15**	V	0.184	0.064	0.220	**SP**
**16**	L	0.296	0.121	0.236	**SP**
**17**	S	0.230	1.005	0.355	**SP**
**18**	P	0.238	0.990	0.253	**SP**
**19**	S	0.321	0.718	0.223	**SP**
**20**	F	0.214	0.989	0.253	**SP**
**21**	Q	0.263	0.719	0.426	**SP**
**22**	S	0.162	0.801	0.802	
**23**	D	0.149	1.004	0.457	
**24**	L	0.280	0.999	0.424	

**25**	G	-	-	-
**26**	S	-	-	-
**27**	F	-	-	-
**28**	K	-	-	-
**29**	W	-	-	-
**30**	S	-	-	-
**31**	T	-	-	-
**32**	E	-	-	-
**33**	L	-	-	-
**34**	I	-	-	-
**35**	D	0.221	0.062	0.263
**36**	W	0.284	1.059	0.344
**37**	S	0.246	1.120	0.388
**38**	K	0.185	0.413	0.492
**39**	I	0.184	0.898	0.288
**40**	T	0.237	0.491	0.238
**41**	D	0.191	0.906	0.254
**42**	Y	0.295	0.682	0.267
**43**	I	0.178	0.517	0.294
**44**	G	0.149	0.990	0.350
**45**	D	0.184	1.050	0.430
**46**	G	0.162	1.033	0.366
**47**	Y	0.199	0.107	0.232
**48**	K	0.185	1.027	**2.700**
**49**	H	0.271	0.558	0.269
**50**	H	0.187	0.079	0.231
**51**	N	0.223	0.100	0.254
**52**	A	0.185	0.303	0.287
**53**	G	0.168	0.479	0.248
**54**	E	0.184	0.215	0.391
**55**	F	0.294	0.114	0.227
**56**	E	0.184	0.215	0.391
**57**	T	1.515	0.990	0.461
**58**	L	0.298	0.180	0.236
**59**	C	0.299	0.190	0.231
**60**	K	0.185	0.076	0.221
**61**	I	0.178	0.074	0.220
**62**	Y	0.295	0.183	0.235
**63**	R	0.211	0.095	0.219
**64**	I	0.190	0.086	0.220
**65**	T	0.226	0.098	0.220
**66**	Q	0.255	0.139	0.227
**67**	A	0.222	0.085	0.222
**68**	E	0.191	0.083	0.222
**69**	A	0.195	0.986	**3.476**
**70**	P	0.210	0.170	0.229
**71**	Q	0.185	0.151	0.227
**72**	P	0.160	0.118	0.220
**73**	S	0.252	0.840	0.243

**74**	F	0.136	0.095	0.220
**75**	E	1.160	0.827	0.253
**76**	N	0.079	0.064	0.221
**77**	R	0.201	0.157	0.228
**78**	E	0.109	0.217	0.222
**79**	K	0.787	0.071	0.286
**80**	E	0.116	0.077	0.222
**81**	N	**4.514**	**4.296**	**6.560**
**82**	E	0.109	0.071	0.222
**83**	I	0.103	0.069	0.220
**84**	L	**3.147**	1.005	0.218
**85**	N	0.087	0.998	0.490
**86**	K	0.110	0.074	0.254
**87**	L	0.204	0.148	0.326
**88**	E	0.109	0.071	0.255
**89**	E	0.116	0.077	0.260
**90**	M	0.106	0.068	0.250
**91**	V	0.126	0.072	0.254
**92**	R	**2.084**	0.095	0.277
**93**	E	0.109	0.073	0.255
**94**	T	1.691	1.039	0.284
**95**	E	0.117	0.074	0.260
**96**	A	0.108	0.071	0.252
**97**	V	0.415	0.131	0.407
**98**	G	0.415	0.092	0.407
**99**	A	0.825	0.865	0.252
**100**	G	0.682	0.987	0.241
**101**	G	0.269	0.360	0.299
**102**	N	0.190	0.895	0.253
**103**	K	1.039	0.958	0.319
**104**	G	1.463	0.800	0.278
**105**	S	1.233	1.465	0.326
**106**	N	0.142	0.826	0.289
**107**	K	1.216	1.015	0.258
**108**	S	1.178	0.165	0.319
**109**	A	0.960	0.920	0.285
**110**	K	0.863	0.922	0.278
**111**	S	0.993	1.058	0.340
**112**	T	0.150	0.951	0.276
**113**	T	0.149	0.089	0.276
**114**	A	0.151	0.087	0.275
**115**	Y	0.123	0.080	0.267
**116**	Q	0.177	0.542	0.312
**117**	E	0.109	0.071	0.255
**118**	I	0.127	0.079	0.260
**119**	R	**3.352**	1.010	1.183
**120**	K	1.424	0.077	0.259
**121**	L	0.195	0.167	0.338
**122**	F	1.361	0.087	0.277

**123**	E	0.109	0.071	0.255
**124**	K	0.780	0.071	1.103
**125**	A	0.147	0.087	0.275
**126**	K	0.117	0.243	0.260
**127**	A	0.111	**2.007**	0.260
**128**	L	0.223	0.817	0.338
**129**	K	0.110	0.090	0.254
**130**	E	0.116	0.100	0.260
**131**	E	0.116	0.510	0.260
**132**	I	0.116	0.458	0.251
**133**	E	0.113	0.090	0.255
**134**	V	0.150	0.562	0.275
**135**	N	0.079	0.080	0.239
**136**	R	0.121	0.102	0.257
**137**	T	0.160	0.152	0.284
**138**	R	0.117	0.489	0.259
**139**	S	0.200	0.741	0.298
**140**	L	0.217	0.218	0.334
**141**	N	0.079	0.072	0.239
**142**	A	0.158	0.144	0.283
**143**	S	0.087	0.304	0.239
**144**	R	0.863	0.632	1.499
**145**	S	0.152	0.667	1.927
**146**	A	0.108	0.092	0.252
**147**	Q	0.185	**4.666**	0.320
**148**	E	0.116	1.062	0.260
**149**	N	0.079	0.072	0.239
**150**	M	0.106	0.388	1.111
**151**	L	0.223	0.215	0.338
**152**	R	0.133	0.124	0.265
**153**	A	0.108	0.092	0.252
**154**	V	0.147	0.122	0.275
**155**	Y	0.123	0.110	0.267
**156**	G	0.190	0.114	0.299
**157**	D	0.144	0.128	0.278
**158**	A	0.108	0.092	0.252
**159**	V	0.147	0.122	0.275
**160**	D	0.080	0.072	1.114
**161**	V	0.147	0.122	0.275
**162**	A	0.158	0.146	0.283
**163**	R	0.133	0.124	0.265
**164**	N	0.079	0.072	0.239
**165**	E	0.109	0.089	0.255
**166**	N	0.079	0.072	0.239
**167**	K	0.117	0.494	0.259
**168**	T	0.111	0.106	0.253
**169**	L	0.223	0.216	0.338
**170**	E	0.109	0.091	0.255
**171**	E	0.867	1.013	0.260

**172**	A	0.207	0.197	0.314
**173**	M	0.106	0.088	0.250
**174**	R	0.133	0.118	0.265
**175**	G	0.145	0.613	0.270
**176**	N	0.146	0.131	0.278
**177**	K	0.117	0.104	0.262
**178**	S	0.230	**2.593**	0.238
**179**	L	0.223	0.166	0.240
**180**	L	0.211	0.156	0.235
**181**	F	0.219	0.164	0.236
**182**	N	0.079	0.062	0.220
**183**	S	0.087	0.508	0.218
**184**	V	1.182	0.879	0.307
**185**	D	0.080	0.066	0.221
**186**	N	1.547	0.684	1.179
**187**	A	0.158	0.092	0.221
**188**	G	1.035	0.998	0.514
**189**	T	0.992	0.813	0.344
**190**	S	0.087	0.066	0.218
**191**	C	0.146	0.163	0.228
**192**	G	0.096	0.067	0.219
**193**	S	0.150	0.105	0.224
**194**	Y	0.993	0.386	0.221
**195**	G	0.143	0.090	0.219
**196**	E	0.142	0.877	0.222
**197**	K	0.108	0.069	0.220
**198**	L	**2.115**	1.142	**3.294**
**199**	V	0.106	0.069	0.220
**200**	G	0.143	0.090	0.219
**201**	M	0.106	0.171	0.220
**202**	T	0.915	0.100	0.221
**203**	L	0.236	0.187	0.244
**204**	I	0.175	0.079	0.220
**205**	N	0.075	0.062	0.219
**206**	D	0.142	0.085	0.222
**207**	F	0.134	0.086	0.220
**208**	F	0.217	0.164	0.234
**209**	C	0.137	0.113	0.228
**210**	L	0.235	0.186	0.239
**211**	C	0.131	0.083	0.219
**212**	V	0.144	0.083	0.221
**213**	G	0.143	0.090	0.220
**214**	E	0.108	0.067	0.220
**215**	A	-	-	-
**216**	I	-	-	-
**217**	N	-	-	-
**218**	V	-	-	-
**219**	T	-	-	-
**220**	D	-	-	-

**221**	E	-	-	-
**222**	M	-	-	-
**223**	R	-	-	-
**224**	Q	-	-	-
**225**	K	-	-	-
**226**	K	-	-	-
**227**	E	-	-	-
**228**	E	-	-	-
**229**	E	-	-	-
**230**	E	-	-	-
**231**	K	-	-	-
**232**	K	-	-	-
**233**	E	-	-	-
**234**	G	-	-	-
**235**	G	-	-	-
**236**	K	-	-	-
**237**	Y	-	-	-
**238**	S	-	-	-
**239**	D	-	-	-
**240**	S	-	-	-
**241**	V	-	-	-
**242**	Y	0.068	0.076	0.220
**243**	N	0.047	0.061	0.219
**244**	G	0.660	0.164	0.258
**245**	F	**4.062**	0.900	**6.082**
**246**	N	0.061	0.061	0.222
**247**	C	0.072	0.068	0.219
**248**	P	0.104	0.084	0.227
**249**	C	0.072	0.068	0.219
**250**	R	0.597	0.162	0.247
**251**	D	0.047	0.061	0.219
**252**	E	0.744	1.004	0.222
**253**	I	0.069	0.065	0.219
**254**	R	0.069	0.068	0.219
**255**	R	0.073	0.069	0.219
**256**	P	0.132	0.123	0.228
**257**	K	0.997	0.141	0.247
**258**	N	0.325	0.385	0.218
**259**	G	0.051	0.073	0.218
**260**	S	0.395	0.061	0.218
**261**	W	0.140	0.118	0.232
**262**	T	0.142	0.094	0.232
**263**	M	0.055	0.062	0.218
**264**	M	0.055	0.062	0.218
**265**	A	0.059	0.066	0.219
**266**	E	0.062	1.134	0.221
**267**	Y	0.714	1.016	0.246
**268**	C	0.072	0.077	0.220
**269**	E	0.062	0.906	0.232

**270**	G	0.092	0.076	0.228
**271**	N	0.525	0.980	0.237
**272**	S	0.079	0.996	0.285
**273**	-	0.415	0.442	0.407
**274**	S	0.907	1.477	0.291
**275**	P	1.079	1.194	0.280
**276**	C	0.120	0.070	0.218
**277**	N	1.819	0.956	0.778
**278**	P	0.186	0.103	0.224
**279**	E	1.688	0.591	0.653
**280**	D	1.599	0.840	0.311
**281**	I	0.115	0.179	0.223
**282**	K	0.106	0.187	0.240
**283**	Y	0.209	0.089	0.223
**284**	N	0.115	0.729	0.233
**285**	H	1.108	0.994	0.962
**286**	T	0.116	0.065	0.221
**287**	E	0.757	0.444	0.357
**288**	A	0.145	0.174	0.226
**289**	W	0.195	0.234	0.250
**290**	D	0.074	0.084	0.222
**291**	V	0.135	0.147	0.224
**292**	I	0.092	0.107	0.220
**293**	S	0.077	0.095	0.218
**294**	K	0.887	1.052	0.364
**295**	A	0.097	**4.363**	0.220
**296**	C	0.186	0.213	0.239
**297**	V	0.135	0.147	0.224
**298**	Y	0.209	0.244	0.251
**299**	K	0.104	0.116	0.223
**300**	S	0.073	0.354	0.221
**301**	V	0.745	1.367	0.311
**302**	A	1.017	**4.478**	0.680
**303**	S	0.200	0.226	0.244
**304**	N	0.075	0.085	0.221
**305**	V	0.144	0.173	0.227
**306**	K	0.099	0.110	0.222
**307**	T	0.098	0.118	0.220
**308**	L	0.188	0.224	0.246
**309**	K	0.106	0.127	0.222
**310**	S	0.076	0.095	0.218
**311**	A	0.929	0.727	0.497
**312**	L	0.221	0.255	0.266
**313**	A	0.096	0.115	0.220
**314**	E	1.723	1.065	0.718
**315**	F	0.121	0.150	0.223
**316**	D	0.659	0.348	0.288
**317**	D	**5.181**	1.626	**6.446**
**318**	L	0.235	0.229	0.269

**319**	V	0.138	0.147	0.228
**320**	N	0.075	0.085	0.221
**321**	L	0.188	0.224	0.246
**322**	E	0.105	0.125	0.223
**323**	Q	0.160	0.195	0.235
**324**	D	0.076	0.084	0.222
**325**	K	0.106	0.587	0.222
**326**	Y	0.110	0.135	0.223
**327**	Q	0.160	0.195	0.235
**328**	V	0.134	0.147	0.224
**329**	R	0.752	0.517	0.360
**330**	G	0.103	0.098	0.219
**331**	I	0.092	0.498	0.219
**332**	F	1.855	1.486	0.362
**333**	G	0.080	0.098	0.218
**334**	Y	0.108	0.135	0.219
**335**	V	0.131	0.147	0.220
**336**	K	0.747	0.630	0.243
**337**	T	0.139	0.703	0.222
**338**	E	0.700	0.445	0.275
**339**	N	0.773	0.636	**3.218**
**340**	N	0.130	0.157	0.220
**341**	D	1.016	0.936	0.557
**342**	N	0.068	0.085	0.220
**343**	R	1.235	0.890	0.484
**344**	T	1.031	0.789	0.388
**345**	C	0.209	0.251	0.226
**346**	T	0.143	0.160	0.221
**347**	G	0.080	0.325	0.218
**348**	H	0.131	0.099	0.219
**349**	T	0.112	0.129	0.221
**350**	A	0.094	0.105	0.219
**351**	G	0.130	0.132	0.219
**352**	F	0.203	0.216	0.227
**353**	T	0.144	0.128	0.220
**354**	C	0.785	0.123	0.218
**355**	V	0.157	0.166	0.221
**356**	S	0.074	0.079	0.237
**357**	Y	0.206	0.218	0.318
**358**	K	1.019	1.050	0.237
**359**	H	1.126	**3.480**	0.318
**360**	T	0.144	0.150	0.274
**361**	L	0.135	0.137	0.273
**362**	E	0.102	0.102	0.259
**363**	N	0.068	0.072	0.244
**364**	G	0.080	0.081	0.240
**365**	G	0.120	0.110	0.254
**366**	I	0.089	0.087	0.249
**367**	P	0.194	0.213	0.310

**368**	W	0.192	0.207	0.319
**369**	Y	0.206	0.218	0.318
**370**	N	0.068	0.072	0.244
**371**	R	1.426	1.202	1.684
**372**	L	0.135	0.137	0.273
**373**	S	1.154	1.004	**2.061**
**374**	N	0.130	0.129	0.270
**375**	A	0.094	0.092	0.250
**376**	T	0.159	0.106	0.275
**377**	E	0.095	0.408	0.256
**378**	Q	0.162	0.181	0.309
**379**	L	0.216	0.215	0.331
**380**	Q	0.170	0.187	0.306
**381**	E	0.102	0.102	0.259
**382**	M	0.092	0.087	0.246
**383**	D	1.197	0.072	0.246
**384**	M	0.824	1.039	0.263
**385**	Y	0.108	0.109	0.262
**386**	A	0.191	0.195	0.303
**387**	K	0.099	0.091	0.254
**388**	E	0.098	0.090	0.256
**389**	A	0.148	0.096	0.250
**390**	D	0.185	0.126	0.270
**391**	S	0.125	0.078	0.239
**392**	H	0.151	0.099	0.252
**393**	L	1.076	1.016	0.273
**394**	H	0.151	0.099	0.252
**395**	E	0.146	0.089	0.256
**396**	L	0.245	0.198	0.314
**397**	E	0.154	0.084	0.259
**398**	E	0.154	0.350	0.259
**399**	Y	0.262	0.192	0.318
**400**	E	0.154	0.405	0.259
**401**	D	0.111	0.177	0.246
**402**	E	0.146	0.076	0.256
**403**	A	0.198	0.120	0.275
**404**	E	0.146	0.076	0.256
**405**	E	0.154	0.084	0.259
**406**	I	0.167	0.089	0.254
**407**	F	0.178	0.100	0.268
**408**	L	0.192	0.531	0.273
**409**	E	0.146	0.076	0.256
**410**	V	**3.980**	0.931	**5.778**
**411**	K	0.148	0.076	0.254
**412**	L	0.194	0.176	0.273
**413**	G	0.132	0.070	0.240
**414**	G	0.132	0.070	0.240
**415**	D	0.111	0.066	0.246
**416**	V	0.187	0.933	0.267

**417**	E	0.146	0.076	0.256
**418**	L	0.275	0.214	0.334
**419**	W	0.250	0.740	0.319
**420**	K	0.156	0.078	0.264
**421**	S	0.125	0.201	0.237
**422**	S	0.210	0.130	0.277
**423**	Q	0.227	0.706	0.306
**424**	G	0.132	0.070	0.240
**425**	K	0.148	0.274	0.254
**426**	G	0.132	0.196	0.240
**427**	-	**2.557**	0.999	0.355
**428**	-	1.242	1.033	0.360
**429**	-	1.816	0.994	0.372
**430**	-	0.138	0.093	0.360
**431**	D	0.111	0.172	0.246
**432**	D	1.150	1.003	0.667
**433**	A	**3.531**	**4.889**	1.310
**434**	R	**4.977**	**5.220**	**5.934**
**435**	V	0.197	0.566	0.276
**436**	D	0.779	0.962	0.246
**437**	N	0.112	0.065	0.244
**438**	D	0.111	0.066	0.246
**439**	G	0.175	0.088	0.254
**440**	L	0.192	0.124	0.273
**441**	T	0.151	0.087	0.250
**442**	H	0.151	0.090	0.252
**443**	L	0.245	0.183	0.314
**444**	N	0.112	0.071	0.244
**445**	I	0.859	0.328	0.249
**446**	A	1.132	0.133	0.275
**447**	T	0.151	0.087	0.250
**448**	G	0.171	0.079	0.254
**449**	V	0.132	0.420	0.240
**450**	A	0.178	1.063	0.268
**451**	M	0.201	0.522	0.274
**452**	L	0.262	0.211	0.325
**453**	L	0.262	0.211	0.325
**454**	I	0.147	1.036	0.253
**455**	I	0.262	**5.004**	0.325
**456**	S	0.292	0.902	0.335
**457**	L	0.192	0.124	0.273
**458**	F	0.141	**2.805**	0.249
**459**	C	0.175	0.111	0.260
**460**	T	0.190	0.517	0.267
**461**	L	0.192	0.124	1.493

Ratio of nonsynonymous-to-synonymous substitutions (ω) along ESAG6 **(A)**, ESAG5 (**B**) and ESAG2 (**C**). Rates at individual codons are estimated by the mean of the posterior distribution of rates, using the prior distribution determined by the model M2a (selection model) parameters

listed in Table 6. See text for details. Estimates with ω >2 are indicative of adaptive evolution and are highlighted in bold. The amino acid sequence shown for each locus is that of the ESAG6, ESAG5 and ESAG2 sequences from TAR clone F44 in *T. b. brucei* with gaps introduced by the alignment. Ambiguously-aligned regions were omitted from the analysis and are indicated by dashes (-). The signal peptide from each ESAG in TAR clone Tbb_F44 was inferred using SignalP3.0 [S1] and is denoted SP in the table. The ESAG6 hypervariable region (HV) (residues 149-159) and box 1 (residues 209-215) and box 2 (residues 223-238) of the transferrin-binding region were assigned as in [39, 40].

S1. Emanuelsson O, Brunak S, von Heijne G, Nielsen H: **Locating proteins in the cell using TargetP, SignalP and related tools**. *Nat Protoc* 2007, 2(4):953-971.
