# Supplementary material for: Isolation and analysis of the genetic diversity of repertoires of VSG expression site containing telomeres from Trypanosoma brucei gambiense, T. b. brucei and T. equiperdum
Source: BMC Genomics. 2008 Aug 12;9:385. doi: 10.1186/1471-2164-9-385 (PMC2533676; doi:10.1186/1471-2164-9-385)
Supplement: Additional file 8 — Sup. Figure 6. The dN/dS ratio (ω) of ESAG6, ESAG5 or ESAG2 calculated from sequence repertoires from T. b. gambiense, T. b. brucei and T. equiperdum plotted against codon number. [file 1471-2164-9-385-S8.pdf]

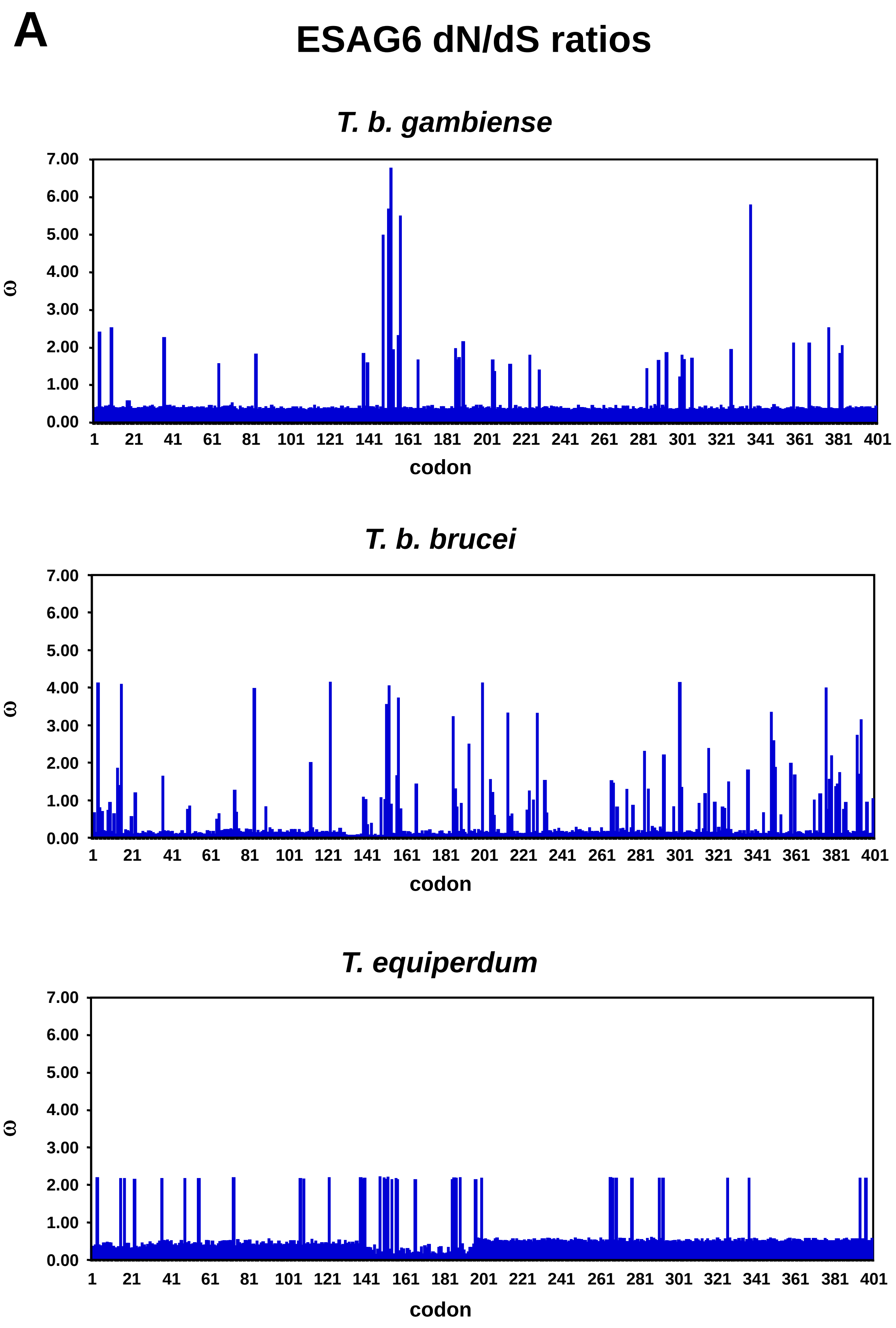
A
ESAG6 dN/dS ratios
T. b. gambiense
T. b. brucei
T. equiperdum
ω
codon
0.00
1.00
2.00
3.00
4.00
5.00
6.00
7.00
1
21
41
61
81
101
121
141
161
181
201
221
241
261
281
301
321
341
361
381
401

B

ESAG5 dN/dS ratios

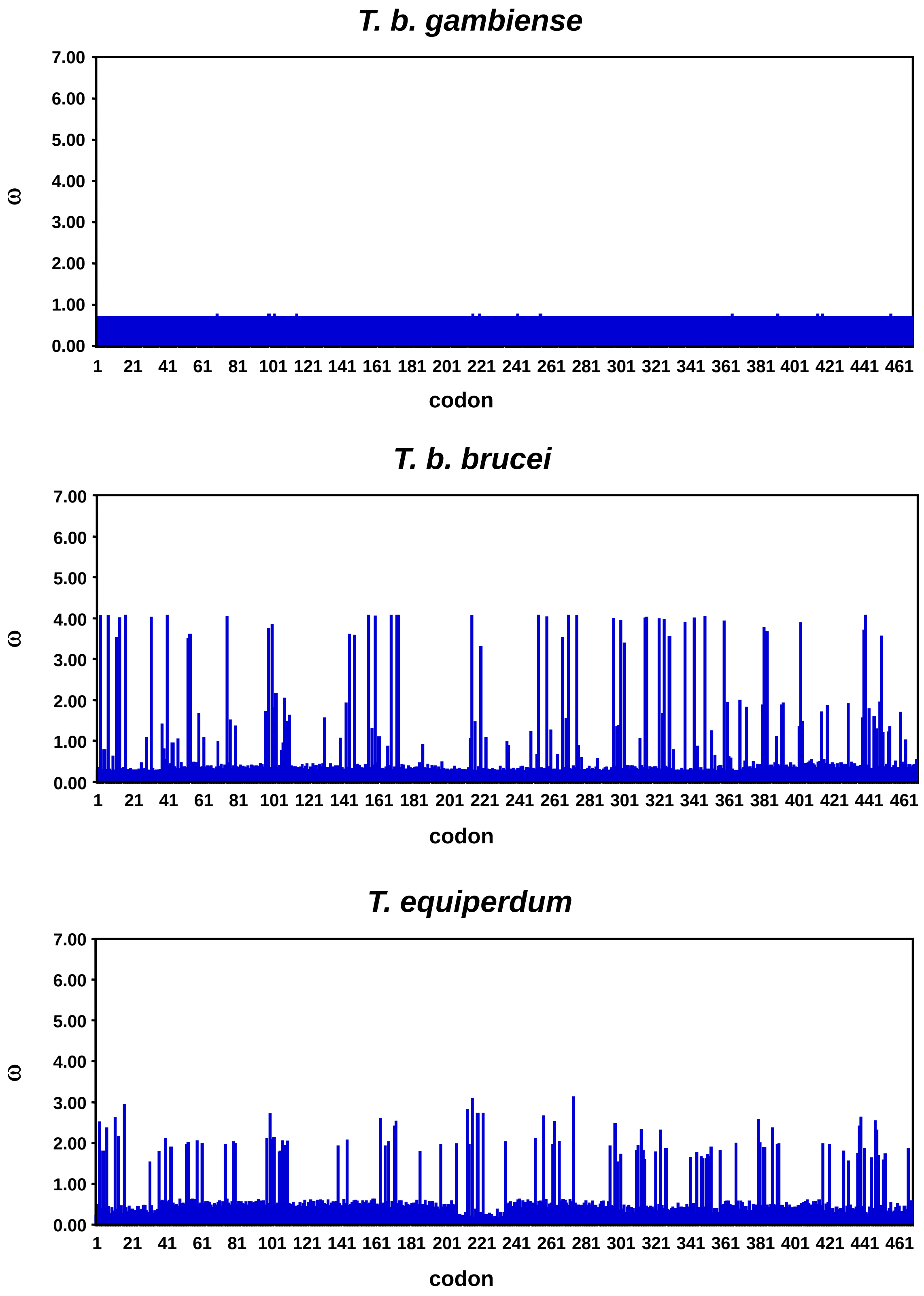

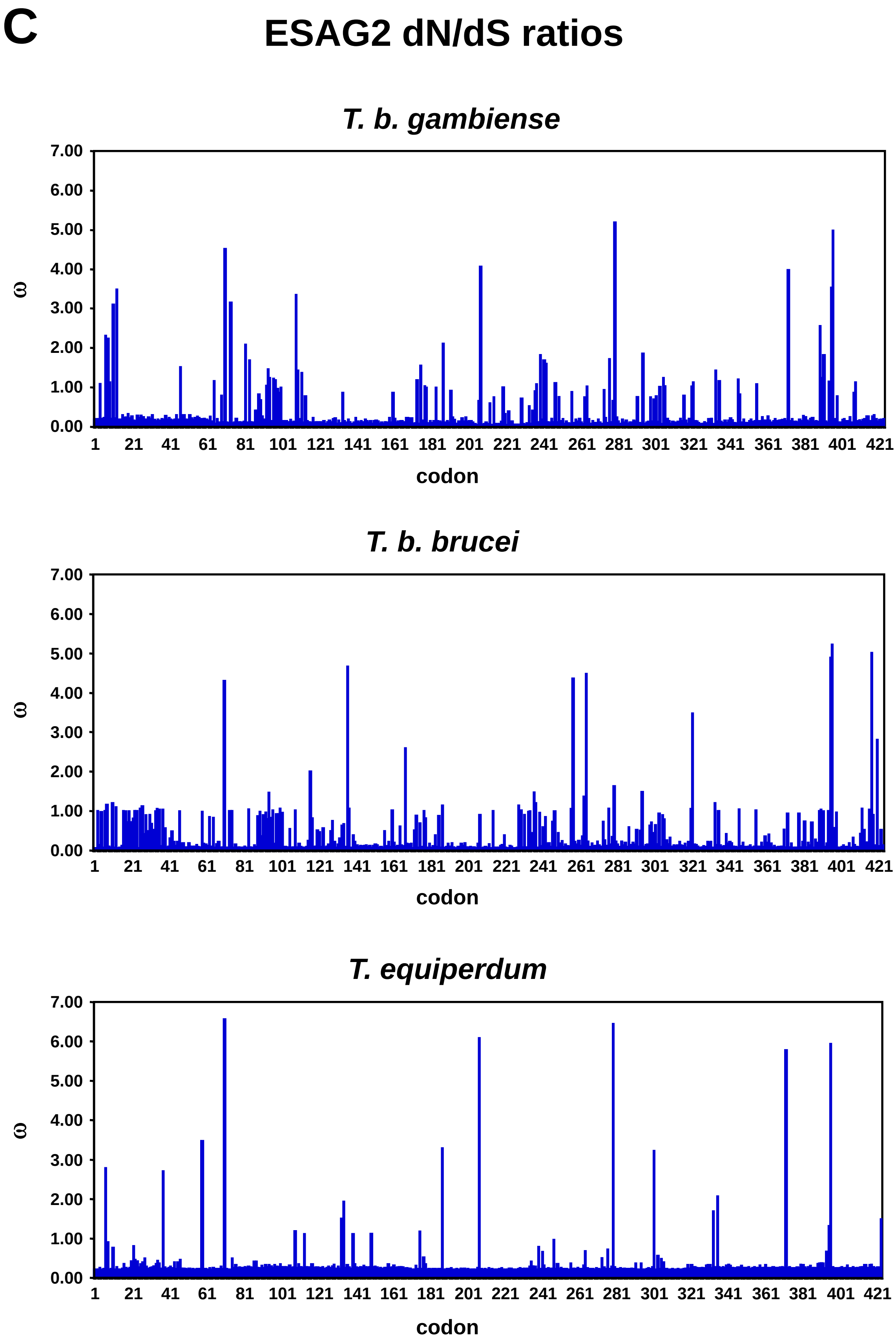
C
ESAG2 dN/dS ratios
T. b. gambiense
T. b. brucei
T. equiperdum
ω
codon
0.00
1.00
2.00
3.00
4.00
5.00
6.00
7.00
1 21 41 61 81 101 121 141 161 181 201 221 241 261 281 301 321 341 361 381 401 421
