# Supplementary material for: Isolation and analysis of the genetic diversity of repertoires of VSG expression site containing telomeres from Trypanosoma brucei gambiense, T. b. brucei and T. equiperdum
Source: BMC Genomics. 2008 Aug 12;9:385. doi: 10.1186/1471-2164-9-385 (PMC2533676; doi:10.1186/1471-2164-9-385)
Supplement: Additional file 9 — Supplementary Table 1. Table of primers used. [file 1471-2164-9-385-S9.pdf]

Supplementary Table 1

Primers used

Primer	Sequence 5'-3'
ESAG6	
ESAG6-UPSs	GCTGYYGCCGAAATRGTAATC
ESAG6-DNas	CCRCTRGAAACTTACGTTAC
ESAG6-287s	CAGTATTGAGGAATGAGTTTAC
ESAG6/7-311s	CCAGCAGGAGTTGGAGGAAATG
ESAG6-652as	CYTCCCATTTCCTCCTCG
ESAG6-796s	GGTGATGCTACAGAGTACGG
ESAG6-809as	CGTACTCTGTAGCATCACCGTATTC
ESAG6-1045as	GTTCACTCACTCTCTTTGACAG
ESAG5	
ESAG5-UPSs	CTTYAGTTGCACTTAGTGTAGC
ESAG5-DNas	GGAAAGCAAGATAGGTTTGAGTG
ESAG5-240s	CSGTGTGAGTGTAGAAGAGAC
ESAG5-578as	CGCCAAGCCACTTAGGC
ESAG5-827s	CCTGTTGCTGTTGTTTCTTC
ESAG5-1007as	GTTCGAATGTRGCCTCAGAG
ESAG2	
ESAG2-UPS1s	GGTTGCGCATGCTGTTTAC
ESAG2-UPS2s	ACAAGCTAGAAGTGTTTCT
ESAG2-DN1as	TATACCAAATAGACGCAGC
ESAG2-DN3as	CCAAATAGACGCAGCAACAAAG
ESAG2-371s	GAAACTGTTTGAGAAGGCG
ESAG2-457s	ATGTTGAGAGCTGTGTATGG
ESAG2-871s	AGTTGGACMATGATGGCTG
ESAG2-505as	TTCTTGCCACATCCACAGC
ESAG2-900as	ACTAATCACATCCCATGCC
ESAG2-970as	ACTAATCACATCCCATGC
ESAG2-1088as	TGACACATGTGAATCCCG

*Degenerate nucleotides Y= C or T, R=A or G, M=A or C, S=G or C.
